# Evidence for Earlier Stone Age ‘coastal use’: The site of Dungo IV, Benguela Province, Angola

**DOI:** 10.1371/journal.pone.0278775

**Published:** 2023-02-24

**Authors:** Isis Mesfin, Maria-Helena Benjamim, Anne-Elisabeth Lebatard, Thibaud Saos, David Pleurdeau, Jorge Matos, Matt Lotter

**Affiliations:** 1 Fyssen Foundation - Museu Nacional de Arqueologia de Benguela, Benguela, Angola; 2 UMR 7194 Histoire Naturelle de l’Homme Préhistorique – CNRS, Muséum National d’Histoire Naturelle, Université Perpignan Via Domitia – Alliance Sorbonne Université, Paris, France; 3 Palaeo-Research Institute, University of Johannesburg, Johannesburg, South Africa; 4 Aix-Marseille, CNRS, IRD, INRAE, CEREGE, Aix-en-Provence, France; 5 Instituto Superior Politecnico Jean Piaget, Bairro Nossa Senhora da Graça, Benguela, Angola; Griffith University, AUSTRALIA

## Abstract

The relationship between Earlier Stone Age (ESA) hominins and the southern African coastal environment has been poorly investigated, despite the high concentration of open-air sites in marine and fluvial terraces of the coastal plain from c. 1Ma onward during the Mid-Pleistocene Transition. Southern Africa provides some of the earliest evidence of coastal subsistence strategies since the end of the Middle Pleistocene, during the Middle Stone Age (MSA). These coastal MSA sites showcase the role of coastal environments in the emergence and development of modern human behaviors. Given the high prevalence of coastal ESA sites throughout the region, we seek to question the relationship between hominins and coastal landscapes much earlier in time. In this regard, the +100 m raised beaches of the Benguela Province, Angola, are key areas as they are well-preserved and contain a dense record of prehistoric occupation from the beginning of the Middle Pleistocene, including sites like Dungo, Mormolo, Sombreiro, Macaca and Punta das Vacas. Accordingly, this paper provides a critical review of the coastal ESA record of southern Africa and a detailed presentation of the Dungo IV site, through a qualitative technological analysis coupled with a quantitative inter-site comparison with contemporary southern African coastal plain sites. Through our detailed technological analyses, we highlight the influence of coastal lithological resources on the technical behaviors of hominin groups, and we propose the existence of a “regional adaptive strategy” in a coastal landscape more than 600 000 years ago. Finally, we argue for the integration of coastal landscapes into hominins’ territories, suggesting that adaptation to coastal environments is actually a slower process which begins with “territorialization” well before the emergence and development of *Homo sapiens*.

## Introduction

The southern African coast starts in the Democratic Republic of Congo and extends southwards all the way down to the Cape of Good Hope and northwards back up to Tanzania. An important topographic feature of this coastline is a plain which extends along its western and southern parts, called the southern African coastal plain. It extends from the shoreline to the foot of the Great Escarpment in the south and continues towards the north where it delimits the Angolan Plateau. The plain is currently shared between Angola, Namibia and South Africa, and is characterized, north to south, by the Zambezian woodland down to Lobito (Angola), the Karroo-Namib arid to semi-arid environment from Lobito all along the Namibian coast, and then the *fynbos* environment which begins in the Cape Province of South Africa [1]. Coastal archaeology is well-developed across this area, but especially in South Africa where there is a rich corpus of coastal sites that document past lifeways and early symbolic behaviors of Pleistocene coastal hunter-gatherers [2–17], and also in Namibia—especially during the Holocene [18]. However, there are gaps in our knowledge pertaining to specific areas and time periods across this plain, and it is unclear whether the adaptive strategies implemented by early humans represent a collective response that facilitated strategic local landscape and resource use.

With this paper, we first provide a review of the available data on human dispersals along the southern African coast, specifically during the Earlier Stone Age (ESA) that reflects the earliest phase of human/coastal interactions. This is followed by a detailed presentation of Dungo IV (Angola), including all historical, contextual, environmental, hydrographical, geological, chrono-stratigraphical and lithic technological details. Finally, we discuss the process of coastal use and ‘territorialization’ during the ESA, to shed light on the earliest evidence of strategic hominin adaptations.

### The emergence of coastal sites and behaviors in southern Africa: Current hypotheses and problems

The southern Cape coast of South Africa preserves evidence of coastal adaptations [3, 19–22]. Here, such adaptations are associated with the Middle Stone Age (MSA) and the emergence of ‘modern behavior’ (i.e., symbolic behaviors, hafting, bone tool technology, fishing technology etc.), reflected in a range of archaeological assemblages with diverse marine faunal remains and in particular, rich shellfish caches [13, 19, 21, 23–26]. The earliest evidence of shellfish gathering in southern Africa is preserved at Pinnacle Point 13, dated to c. 164 ka [27], and the early symbolic use of shells is also associated with the MSA, c. 75 ka, at Blombos Cave [28]. These ‘coastal behaviors’ become more prevalent with time [24], particularly during the Later Stone Age (LSA) [19, 29] that emerges during the Final Pleistocene [30].

However, different types of coastal behaviors occurred in the past, and these have formed varying signatures from dense shell middens to scarcer lithic scatters [31, 32], or evidence related to ephemeral beach activities such as algae collection or cetacean scavenging [33–35], where evidences are prone to disappearing with the tides. The invisibility of some of these coastal activities is well-known though, captured in southern African ethnographic records since the 17^th^ century [35–40].

Coastal resources include all those available to humans adjacent to the coast, directly linked to these types of environments, and they can either be vegetal-, animal- (marine and terrestrial) or mineral-based. “*Coastal adaptation*” is defined as the systematic use of coastal resources within technical and subsistence strategies [3, 22, 41]. However, if coastal adaptation implies coastal resource acquisition, coastal resource acquisition does not always depict a coastal adaptation phenomenon. Consequently, it is hard to assess a coastal adaptation phenomenon without relying on a large corpus of contemporary sites, where we can then collectively assess multiple strands of evidence to develop more holistic understandings of early human behavior. When the archaeological record reflects coastal resource exploitation, that are not the primary subsistence strategy focus, then the expressions “*coastal use*” [41] and “*coastal engagement*” [42] have instead been suggested.

Several authors have explored why numerous ESA coastal sites have been discovered, coupled with only much younger evidence for “*coastal adaptation*”, and varying arguments have been proposed to account for this. Among the arguments, coastal environments are regarded as being complex, unstable and unsafe, thus creating a cognitive barrier [16, 43–46]. Consequently, pre-sapiens hominins would have conducted a ‘passive’ colonization of coastal areas, whereas *Homo sapiens* would have conducted a more ‘strategic’ one [47] leading to a heavier reliance on coastal resources from the very end of the Middle Pleistocene [48]. Yet, great importance is assigned to aquatic, coastal and marine resources and their facilitation of biological and social development of early humans [43, 45, 49–56]. Consequently, one may ask why there is such a scarce record of aquatic resource acquisition during the ESA?

However, we must consider that most coastal activities may remain invisible to archaeologists, especially early ones in Pleistocene open-air contexts during the ESA [57]. This inequality in archaeological visibility is due to one, the type and spectrum of targeted resources and their ability to fossilize (e.g. shell, seaweeds, cetacean’s fat, oil and meat, lithic etc.), two, to site formation process—especially for the Pleistocene (cave *vs*. open-air sites), and three, to site location (e.g. on-beach activities *vs*. movement of coastal resources toward living sites). Well-preserved cave deposits along the southern Cape coastline of South Africa provide suitable excavation conditions and they provide a detailed vision of MSA subsistence and technical behaviors along the coast. In contrast, very few coastal ESA sites have been excavated and when they have been, they barely preserve fauna, their site-formation processes are complex, and/or the archaeological deposits are usually disturbed [32, 57–61]. Consequently, the contrast that exists between ESA and MSA site preservation and data resolution is highly likely to impact our current understanding, and this should be considered more carefully when we explore the proliferation of technical behaviors and strategies, especially if there is the potential for some of these activities to be more deeply rooted in time. In addition, since the beginning of the 20^th^ century authors have reported that ESA artifacts are present in coastal deposits (marine terraces, also called “raised beaches”, river terraces from “near coastal rivers” [56] and sand dunes) all over southern Africa [see review in 62], and especially along the Atlantic coast where the arid and semi-arid environments of the Karoo-Namib and the Cape *fynbos* prevail [62–66]. Good examples are provided by Klasies River and Pinnacle Point, where ESA artifacts are numerous and found scattered on the surface [32, 65, 67], in contrast with the well-stratified and -preserved MSA cave records in the same area [3, 24, 68, 69]. This confirms the antiquity of human occupation along the coast, prior to MSA coastal adaptation, but also points towards potentially different trends between MSA and ESA coastal landscape occupations [70].

Very few ESA coastal sites have been studied, but collectively these sites have the potential to clarify the earliest human dispersals within the southern African coastal plain and they may be significant for understanding the emergence and development of complex subsistence and technical behaviors associated to MSA. Indeed, the ESA sites may also highlight a more deeply-rooted emergence of coastal behaviors in southern Africa, which may move us away from the idea of ‘rapid changes’ related to the emergence of *Homo sapiens* and MSA modernity [57, 61]. This impression of rapid MSA change must be questioned while considering preservation biases and the visibility of past human activities. Lastly, the impressive coastal MSA record contrasts with the ESA open-air coastal sites, but this may be the result of a research bias at least partially driven by epistemological forces (such as the tripartite division of the Stone Age, the ‘quest’ for “modernity evidence”, and a focus on ESA karstic and hominin-bearing cave deposits *versus* terraces). The coastal MSA hypothesis may also be related to a lack of contemporary MSA data from inland southern African, which has been discussed recently by colleagues [26].

### Earlier Stone Age site distribution, chrono-cultural and paleoenvironmental contexts

The geopolitical context of southern Africa has influenced the scientific narrative and research traditions across this region, leading to biases in our knowledge accumulation [71–74]. The division of the southern African coastal plain, across geopolitical boundaries, has created differing research traditions and goals by country. It has led to inequalities in site valorization and has further hampered our vision of early coastal occupations across the broader coastal plain. In some areas, such as the Cape provinces, focus has been on understanding the emergence of coastal modern behavior, whereas other regions are helping to ‘fill the gap’ while clarifying coastal site distributions [16, 42, 61, 75]. To provide some examples, in Namibia most coastal plain research has been focused on geology, given the important diamond bearing deposit caused by the Orange River drainage system, with work subsequently being supported by diamond mining companies [76]. Numerous ESA, MSA and more recent coastal sites have been reported here, but they remain unstudied [60]–contrary to the Holocene sites [18]. In Mozambique, only a few coastal sites have been reported, such as Maputo (named “Revez Duarte” in the publication) by Barradas [77], Buzi Company and Ponta Maona reported by Barradas in Clark’s *Atlas of African Prehistory* [78]. Finally, in Angola, many surface finds have been reported along the coast with a higher density of artifacts in the Benguela Province and at Palmeirinhas, south of Luanda [79]. However, this corpus is mainly published in Portuguese or in Portuguese editions, and remains poorly disseminated and integrated into modern research.

**Fig 1:**
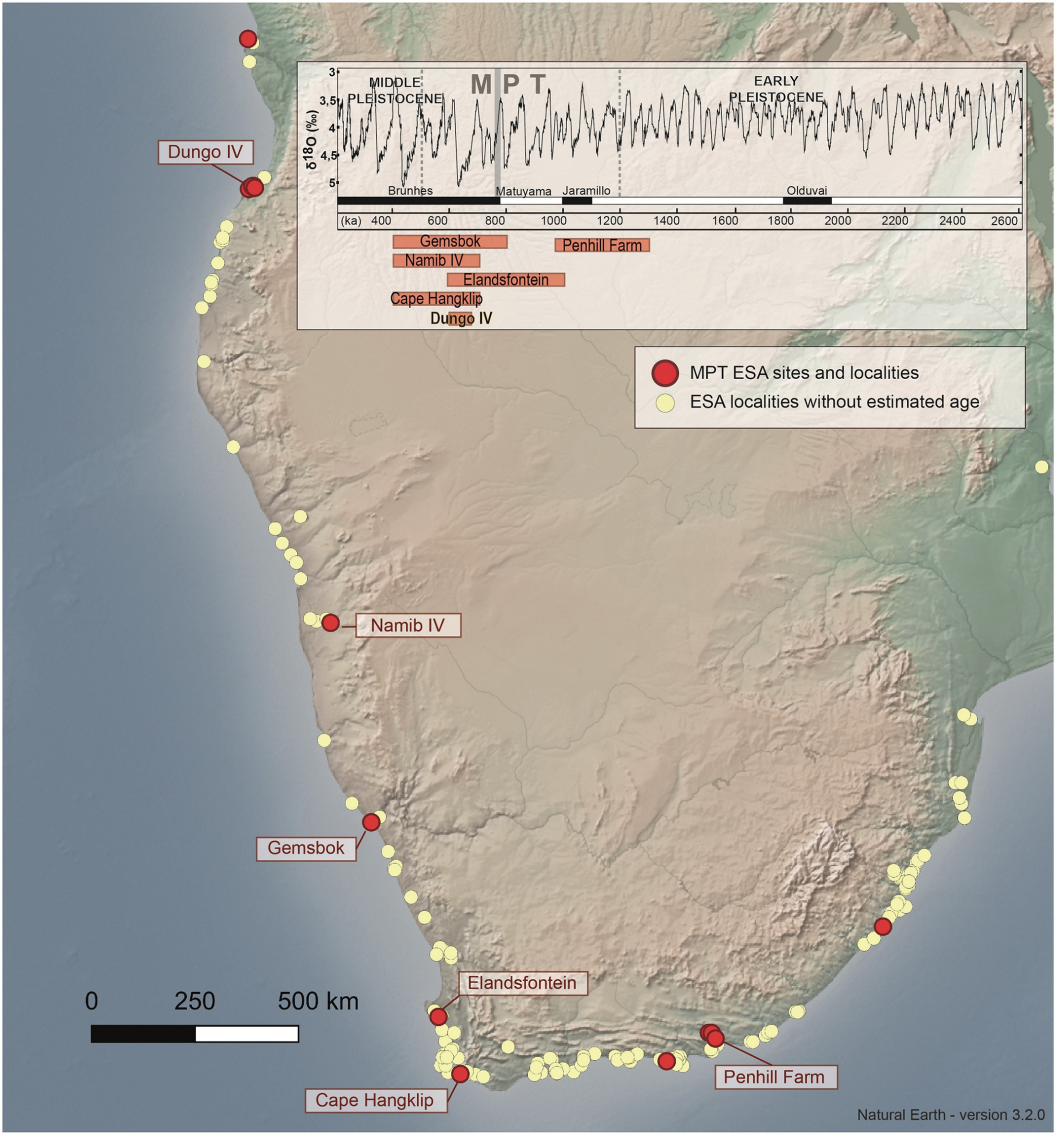
Map and chronology of coastal ESA localities and localization of sites considered in the paper. For details see S1 Table. Reprinted from Natural Earth under a CC BY license, with permission from Natural Earth, original copyright 2022.

We have built up an exhaustive list of coastal localities (excavated and surface sites) that are reported as bearing ESA evidence in the southern Africa coastal plain (Fig 1 and S1 Table). The list contains 237 localities reported from the 20^th^ century until now. Due to the antiquity of some publications, the absence of dating, the lack of nomenclature homogeneity among lithic techno-types, and the unstandardized site descriptions, we have not attempted to assign them chrono-cultural attributions (i.e., within the global ESA, e.g. “Pebble Culture”, “Oldowan”, “early/middle/later Acheulean”) and instead, we have opted to consider them as “un-diagnostic ESA” localities [80, 81]. A large part of them were reported by O. Davies while he traveled east to west, following the coast to provide a global view of the quaternary paleo-shorelines and their relation with inland river basins geomorphology [63, 64, 82–87]. One must also consider that many assemblages were gathered, stored and/or registered in museums, unpublished, as is the case for several Angolan and Namibian sites at the Museu Nacional de Arqueologia de Benguela and at the National Museum of Namibia, respectively. As a direct result of this, the authors were also able to find assemblages for Dungo XIII, Pima and Lüderitz (see S1 Table). Overall, our synthesis shows a high density of ESA localities along the coast between Luanda and the current South African-Mozambican border, and their distribution coincides with the southern African coastal plain and the extent of the preserved Pleistocene coastal deposits [88]. Naturally, this raises questions for the southern African ESA and whether there existed a coastal-specific occupation, or whether this distribution merely represents a preservation bias. However, when comparing our synthesis with the corpus provided by other coastal areas of Africa (e.g., preserved raised beaches along the Red Sea coast), the southern African ESA site density appears to be dense [89].

From a chronological perspective, despite the small number of dated sites among marine terraces (also called “raised beaches”), we regard the main coastal ESA sites as: Namib IV and Gemsbok in Namibia, and Elandsfontein, Cape Hangklip and Penhill Farm in South Africa (Fig 1 and Table 3). For Angola, only Dungo IV provided reliable dating and a representative lithic assemblage within a Middle Pleistocene raised beach. Collectively, these ESA sites are those with the most reliable lithic assemblages, locations on the coastal plain, and they all have a minimum date of 400 ka.

Most of these sites are contemporaneous with the middle and later Acheulean, from at least ca. 1 Ma onwards [90] and from a paleoclimatic perspective with the Mid-Pleistocene Transition (MPT), which spans c. 1.2 to 0.5 Ma [91–93]. It is difficult to delimit the MPT boundary, due to the gradual emergence of c. 100ka climatic cycles, but the transition begins with an increase in glacier volume between 1.25 Ma and 0.7 Ma [94, 95]. During this transition from the Early to Middle Pleistocene (boundary fixed at 0.781 Ma), the change in climatic cycle duration (from c. 40 ka to c. 100 ka) led to more pronounced global climatic changes and, more specifically, drier Glacial periods with more humid Interglacial periods [96]. The MPT is also associated with strong climatic oscillations which may have impacted human and animal dispersal [97], and southern Africa may have formed a refugium at the very beginning of the Middle Pleistocene [98]. This change may also be partly responsible for the formation of raised beach systems, and it was a major event that contributed to the extension of the coastal plain in South Africa during lowered sea levels [99, 100]. Some parts of the South African coastal plain are very narrow (where the shoreline is very close to the foot of the Great Escarpment) and it is assumed that marine transgressions may have completely submerged the coastal plain during periods of high sea level, directly impacting human occupations [100–103]. However, due to variability in seabed depth, coastal plain width and topography, assessing these occupations is complex and it can only be tackled with a combination of local approaches [104]. It is also particularly important to clarify site-scale geoarchaeological issues for sites within 20km of the coast, that remain buried in river or lacustrine deposits, as they could have been much further inland if they were occupied when the coastal plain was “open” [101] or much closer to the coast and the coastal plain was “closed” [105].

From a paleoenvironmental perspective, the current southern African Atlantic coastal plain environment, namely the Namib desert associated with the upwelling Benguela current, is believed to have been established. But, alternating climatic cycles leading to arid and semi-arid environments was common since at least the Early Pleistocene [106–108]. The upwelling Benguela may have mitigated the importance of climatic oscillations in this region, even though a progressive decrease in surface sea water temperature is recorded between c. 1.5 and 0.5 Ma [96, 109, 110] that we must correlate to aridification of coastal environments in western southern Africa [111, 112]. Consequently, hominin occupation of the coastal plain occurred during a paleoclimatic trend to aridification.

From an African Prehistory perspective, since c. 1 Ma, important changes are noted [113] in terms of technology—corresponding to the transition to the “middle Acheulean”–[90, 114, 115], subsistence behaviors [116] and mobility [117], which collectively may also indicate increasing demographic dynamics [118, 119]. In addition, southern Africa site density may already provide an indication of regional demographic changes ca. 1 Ma [120–122]. However, it remains unclear to what extent this can be linked to shifts across the MPT, and/or to the development of *Homo* across the African continent, considering that no other hominin genera are encountered from c. 1 Ma [113, 123, 124].

### An earlier emergence for coastal sites and behaviors? Existing hypotheses and questions

MSA behavioral trends along the southern African coastal plain reflect complex technology and symbolism, along with strategic food resource exploitation [3, 8, 14, 20–22, 24, 28, 125–128]. Even though ESA human behaviors on the coast remain poorly investigated and are primarily understood by describing lithic remains, they prove that humans colonized coastal areas in southern Africa well before the emergence of the MSA. These ESA hominins must therefore be considered in the broader context of changes associated with the MPT, though no correlation has yet been established. There is only sporadic detail in the literature on the presence of ESA hominins in the southern African coastal plain, for a variety of reasons, and below we summarize the following:

- Coastal raw material procurement: use of sandstone and quartzite pebbles and cobbles [85], as seen along the Eastern Cape coast, or silcrete block procurement along the coast in Namaqualand [5]. This may help to explain the scattered nature of surface artifacts as described by authors.
- Rivers as dispersal corridors: hominin migrations being linked to river courses that were conducive to progressive settlements along the lower part of river basins, eventually leading to coastal areas [57, 60, 129–131].
- Coastal refugium: the likelihood that coastal areas served as a refugium for human and animal populations [59, 100, 132–134]–especially during increased aridity inland. In the Cape Peninsula, Glacial periods are characterized by more humid climates [133, 135]. Further north, the Namib coastal desert would have been occupied by humans during semi-arid periods, which facilitated the formation of pans and savanna that may have prevailed during quaternary interglacial periods [136–139]. This hypothesis has also been formulated to explain the high-density of MSA sites in the Paleo-Agulhas Plain [20].

Questions concerning the relationship between coastal occupation and river courses remain unanswered: are these occupations specifically “coastal occupations”, or are these peripheral “riverside occupations” as seen elsewhere in southern Africa? Also, what would be the driving force behind such a coastal demographic phenomenon: coastal aridification during the MPT, the opening of the coastal plain c. 1Ma, or demographic changes during the Acheulean? Another issue is how to address the relationship between these coastal settlers and their resource exploitation patterns with the absence (or notable scarcity) of organics. Nevertheless, the distribution of sites and these different hypotheses lead us to an important finding: coastal spaces begin to form part of group “*territories*”–irrespective of whether their use is marginal or fully integrated. “*Territory*” is defined here as a space for human ideas and practices, which includes limits, designation and organization [140–144]. Consequently, “territorialization” is the process during which a space gets integrated into a group’s territory through the development of new practices associated with specific locations [143, 145–147]. Indeed, territorialization is the process linked to human settlement during which ‘territorial behaviors’ are developed [148]. ‘Territoriality’ refers to a type of human behavior, where ‘*territorial behaviors*’ concern all aspects of the relationship between human groups and the space they exploit (e.g. delimitation and division of space, denomination, landscape management, property, etc.) [148–150]. The sole fact that ESA populations settled on beaches to produce stone tools, or to supply raw materials, shows that these areas were related to territories—even though we do not have direct evidence for coastal resource foraging.

### The contribution of the Angolan coastal record

An important shortfall of the hypotheses summarized above is that the authors were working with limited spatial datasets that were unable to provide a more holistic vision of coastal ESA occupations across southern Africa. In fact, no hypotheses have considered integrating data from the Angolan coastal plain. Publication language, a lack of dissemination of Angolan data beyond its borders, and the persistence of two wars (1961–1975; 1975–2002) have all contributed to isolating research in Angola [151, 152].

There are two primary coastal ESA localities that have been excavated in Angola, Palmeirinhas by J.D. Clark and C. Ervodesa in the 1960s and 1970s [79, 132] and Dungo IV by a Franco-Angolan team led by M. Gutierrez and M.H. Benjamim since the 1990s [153–155]. The Dungo sites are located on a +100 meter raised beach south of Baía Farta in the Benguela Province. Only Dungo IV has been dated, to between 614.5 ± 9.5 ka and 662.05 ± 10.24 ka [156], providing the earliest evidence for hominin presence in the country and making it one of the only dated coastal ESA sites in southern Africa. In fact, Dungo IV is now the most northern dated and excavated site of the southern African coastal plain, and it also marks the current northern limit of the Karoo-Namib biome. Along with Elandsfontein, Gemsbok and the Lower Sundays River sites, Dungo IV is a key-site for understanding early hominin expansions and the process of coastal territorialization along the coastal plain of southern Africa, during the MPT. In addition to Dungo IV, the Benguela Province also comprises a large number of unexcavated sites, but these are assumed to share a similar stratigraphic position among the +100 meter raised beaches, among which: Mormolo, Punta das Vacas, Sombreiro, Pima, Macaca and Uche [157, 158] indicate an extended distribution of ESA sites in the area.

To provide insight on the occupation of coastal areas and hominin behaviors in the southern African coastal plain, during the ESA and prior to regional MSA coastal adaptations, this paper focuses on Dungo IV and its micro- and macro-regional context. The site is dated, it preserves a large quantity of lithic artifacts, and it forms part of a broader network of ESA coastal sites that are well-preserved among the raised beach deposits of the Benguela Province. Here, we review the history of the Benguela raised beach sites (geology and archaeology), the chrono-stratigraphic context of Dungo IV, and for the first time we present detailed results for a qualitative technological analysis of the lithic assemblage. Finally, we discuss Dungo IV in relation to the broader context of early hominin coastal occupations in southern Africa. By providing clarity on the Benguela coastal ESA sites, we believe they will shed light on territorialization during this period and consequently, will allow us to explore the initial steps that humans implemented when adapting to coastal environments and resources.

All necessary permits were obtained for the described study, which complied with all relevant regulations (Permit 01/GD/MNAB/2020 provided by the Museu Nacional de Arqueologia de Benguela—Article 8 of the Organic Statute).

## Dungo IV and the pleistocene deposits of the Benguela Province

### Environmental and hydrographical settings of the Benguela Province

The coastline of the Benguela Province extends over a steppe coastal plain dominated by shrub and herbaceous plant taxa, including *Acacia*, *Commiphora*, *Colophospermum*, *Aristida*, *Schmidtia* and *Setaria* [1, 159]. The plain extends from the town of Sumbe to northern Namibia. The prevailing climate and vegetation are conditioned by the strong dry offshore winds and the direction of the ocean currents [160]–especially driven by the Benguela Current [161]. The climate is tropical with concentrated rainfall in summer and a dry period in winter [162]. This coastline is part of the broad West African marine ecoregion that extends from Cape Blanc in Mauritania to Cape Frio in Namibia [163], but it is also integrated into the coastal kingdom of “Temperate Southern Africa” [164]. The southern coast of Angola is an important thermal boundary between tropical western Africa and southern Africa with the meeting of warm currents from the north and temperate-cold currents from the south [163].

Despite the lack of paleoenvironmental data for the Benguela Province, on the +50 meter raised beaches of Baía das Pipas, between the cities of Benguela and Namibe [163], Middle Pleistocene malacological assemblages indicate ’Senegalese Fauna’ down to southern Angola. Senegalese Fauna is a typical West African tropical malacological suite that extended into North Africa during interglacials [163]. However, this faunal assemblage is associated with a malacofauna that also reflects a more temperate climate, the presence of which would be due to upwelling induced by the Benguela Current. The conclusions of this work are in line with those of [165] for the Middle Pleistocene, whereby it has been suggested that climatic conditions close to those in the region today occurred during the Pleistocene. Similar settings have also been suggested for the coastal zone of southern Namibia during this period [60].

**Fig 2:**
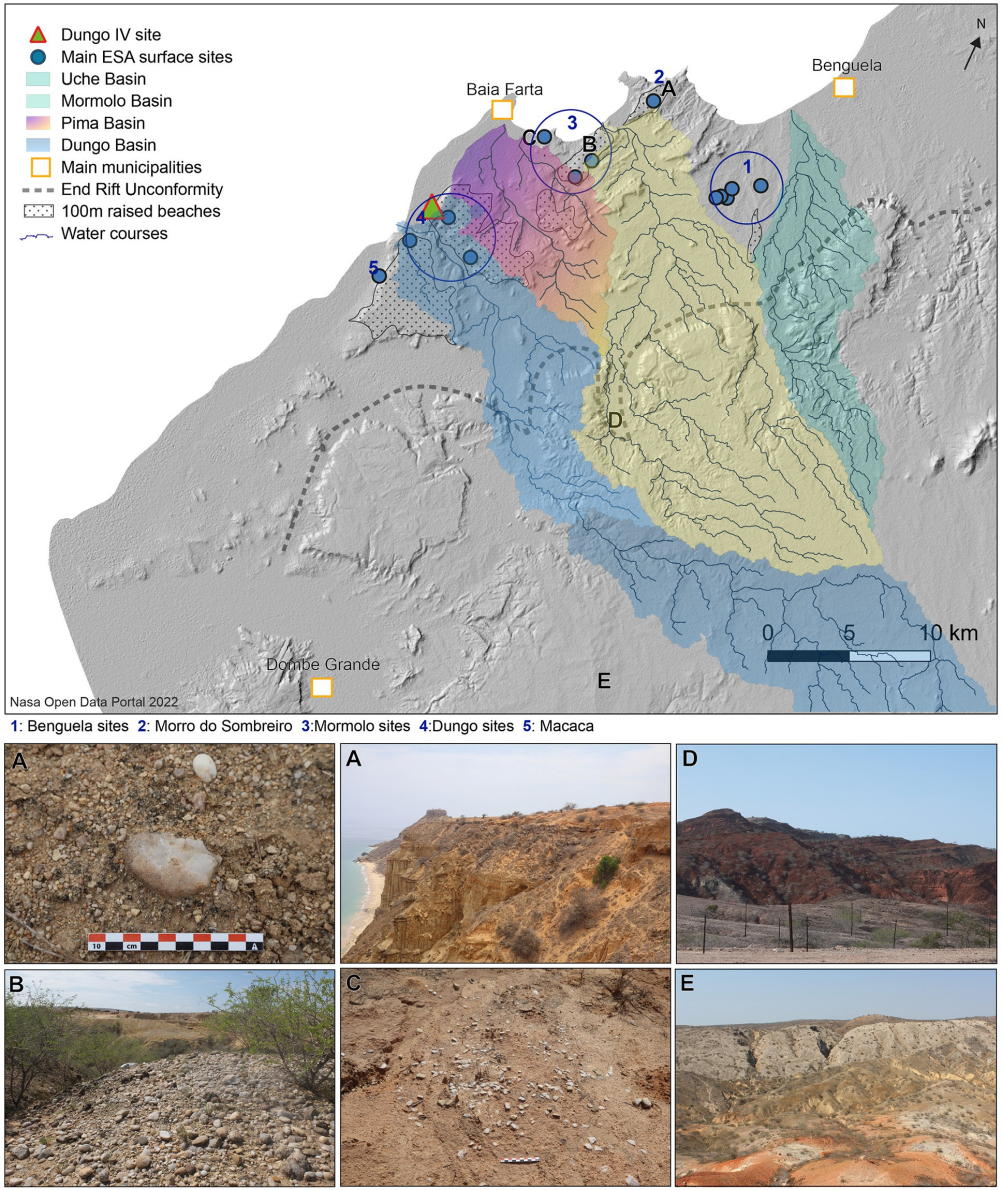
Locality of the ESA surface sites and Dungo IV on the +100 m raised beaches between Benguela and Dombe Grande, based on Museu Nacional de Arqueologia de Benguela records, surveys and test-pits *(157*,*158)*, and hydrographical settings of the areas. The photo letters refer to the letters on the map and show Morro do Sombreiro Red Sand deposits on the +100 m raised beach (A), the cobble/pebble outcrops of the +100 m raised beaches at Mormolo (B), a quartz lithic workshop on a deflated surface within the base of the Red Sand deposit of the +100 m raised beach at Ponta das Vacas (C) and the End Rift Unconformity landscapes (D and E)–Hydrographical settings have been mapped by J. Matos and photos are from I. Mesfin. Reprinted from Nasa Open Portal under a CC BY license, with permission from Nasa Open Portal, original copyright 2022.

The Benguela Province occupies a total area of 39,508 km^2^ with altitudes ranging from sea level to 2,547 m towards the east. The province is primarily encompassed by the Catumbela and Balombo river basins, and from Lobito to 60km south (Dombe Grande) there is a coastal strip with an average width of 20 km where the altitude does not rise above 280 m, forming the coastal plain that is delimited inland by the End Rift Unconformity and the foot of the Angolan Plateau. The strip is characterized by well-preserved Mid-Pleistocene to Holocene raised beaches (Fig 2), and the area of archaeological interest is located between the basins of the perennial Cavaco River (3,983 km^2^) and the Coporolo River (9,505 km^2^). Between these rivers there are small hydrographic basins with streams that only carry water when sporadic heavy rains occur (locally named “*rio secco*”). These smaller non-perennial streams barely extend inland, and from north to south they are the: Uche (103 km^2^), Mormolo (288 km^2^), Pima (78 km^2^), Dungo (389 km^2^) and Calupete (221 km^2^) rivers (Fig 2). In the terminal outflow of these basins, the topography of the terrain is unique given the presence of raised beaches, where abundant prehistoric sites are encountered whose names often relate to these non-perennial river courses [79, 157, 158].

### Geological context of the raised beaches

**Fig 3:**
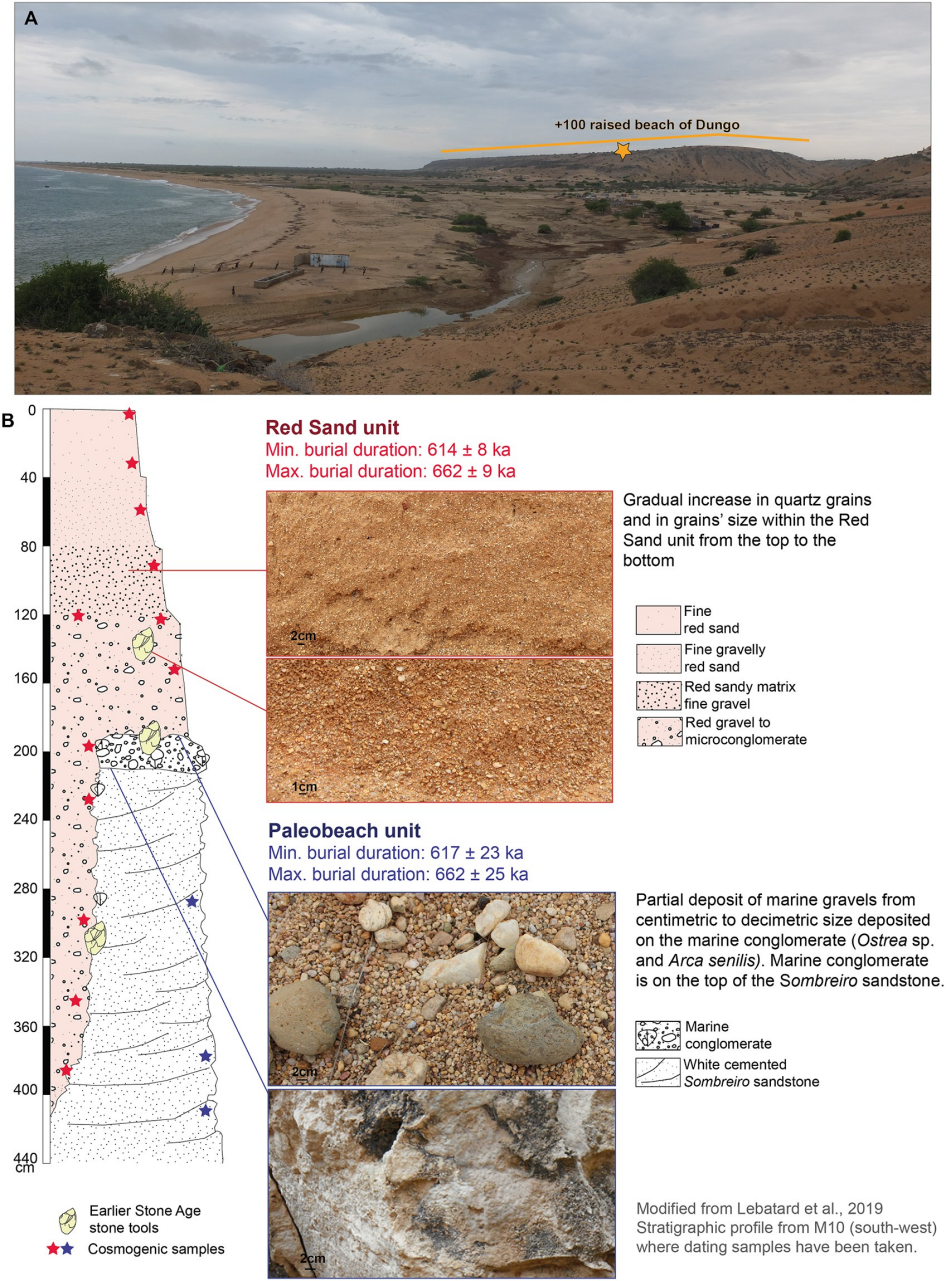
A: Stratigraphy of Dungo IV from 2018–2022 fieldwork; B: overview of the geological features of the +100m raised beach at Dungo IV; C: a layer of quartz artifacts on the top of the layer « red gravel to microconglomerate » of the Red Sand unit, identified in 2022 within the profile of a test-pit from 1994.

**Fig 4:**
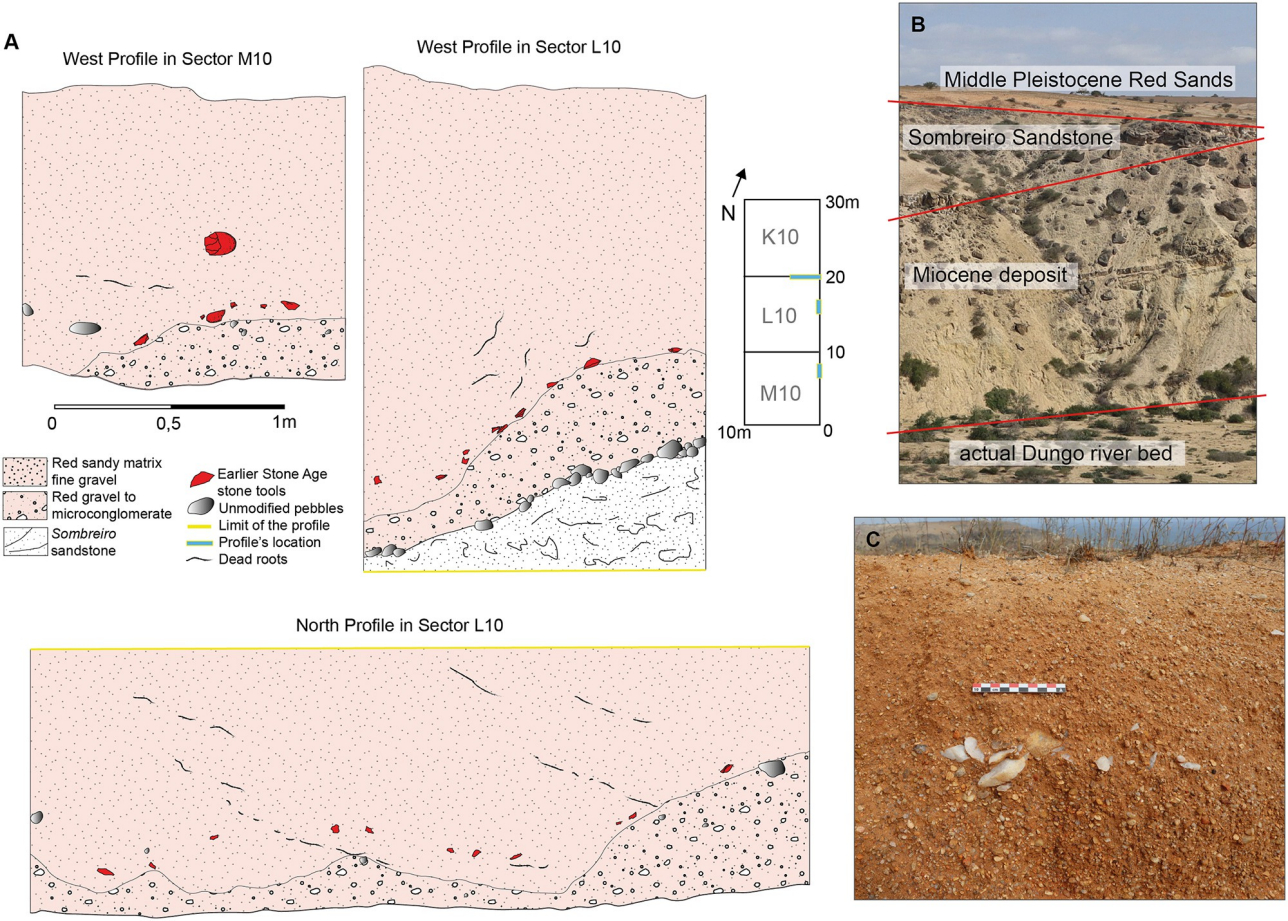
A: View of the +100 m terrace from the estuary of the Dungo river. B: Dungo IV’s sampling locations, stratigraphic log and modeled burial durations [modified from 149].

The sites discussed here have all been identified in these marine formations and they are associated with the highest Quaternary raised beaches (or marine terraces), whose altitude varies between 90 and 150 m above sea level and having been referred to as “Acheulean terraces” by others [79, 153, 157, 166, 167]. These terraces have five main geological units (Fig 3B). First, two Tertiary units are identified:

- a siltstone deposit attributed to the Miocene (23.03 Ma—5.33 Ma) [79] or possibly as old as the Paleocene (66 Ma-56 Ma) [168],
- a deposit of sandstone, in the literature called ’*Sombreiro* sandstone’ [166].
- Second are the four Pleistocene units:
- a ‘marine conglomerate’ in which many shells are observed, including *Arca senilis L*. and *Ostrea* sp. whose age is attributed to the Calabrian (1.8 Ma—0.78 Ma) [160, 166],
- a deposit of gravels with clasts ranging from 1 to 20 cm in diameter, interpreted as a fossil beach level [157] that served as a raw material outcrop for ESA hominins in the area [169].
- an overlying, thin white sand layer on top of the conglomerate, but reported only in some localities [153, 158],
- a sandy deposit, namely the ‘Red Sand unit’, that is up to 20 m thick and dated to the Middle Pleistocene (possibly of deltaic origin). The Red Sand unit is graded beginning from the soil surface with a layer of fine-grained red sand with few fine gravels to red microconglomerate (Figs 3C and 4B). The basal meters of the Red Sand unit contain the ESA artifacts [153, 156, 157, 170].

The altitude of these marine terraces is the result of vertical tectonic uplift and significant erosion of the Tertiary formations during the Quaternary, creating a stratigraphic hiatus between the end of the Miocene and the beginning of the Pleistocene [88, 160, 165, 170]. [160] attempted to date the marine terraces between the towns of Lobito and Baía Farta by running ^230^Th/^234^U analyses on mollusk shells. They obtained ages for the 8–10 m (36 ± 3 ka), 10–12 m (91 ± 6 ka), 20 m (103 ± 5 ka) and 25–30 m (71 ± 7 ka) terraces, but were unable to date the terraces beyond +100 m. However, these dates are debated and revision is needed [171]. Irrespective, these dates suggest that the low-lying marine terraces were formed throughout the Late Pleistocene (126 ka-11.7 ka BP)–a period with increased tectonic uplift [171]. Other colleagues [170] distinguished three types of marine terraces from Lobito that J.D. Clark previously correlated with terraces of southern Luanda [132], namely: “Ouljlian terraces” (10–30 m) spanning the Upper Pleistocene, “Tyrrhenian terraces” (30–50 m) spanning the late Middle Pleistocene, and the “Old terraces” also named “Acheulean Terraces” (90–150 m) or “+100 m raised beaches”, attributed to the Middle Pleistocene and which are the focus of this paper.

From a broader perspective [88], distinguish two types of marine terraces in southern Africa: those which have a vertical uplift rate of 0.01 mm per year, and those in Angola and Namibia where the uplift rate is much higher, estimated to be 0.07 mm per year. This difference resulted in higher raised beaches in Angola and more pronounced stages than in the Cape regions, for example, where the marine shelves are less subject to tectonic uplift [172]. The oldest raised beaches on the Atlantic coast of southern Africa are estimated to be younger than 1 Ma and older or contemporary with the Middle Pleistocene [88, 100, 102]. Therefore, the oldest raised beaches are prime areas to address Acheulean coastal settlements during the MPT.

### Review of Earlier Stone Age research

The richness of the Pleistocene archaeological record in the +100 m raised beaches has been reported since the 1950s by several geologists prospecting the area for resource exploitation purposes. However, it is when J.L.M. Pais Pinto created the *Museu Nacional de Arqueologia de Benguela* in 1976 that archaeological excavations were initiated at Dungo. He also prospected the coastal zone around Benguela and discovered several new sites among which several localities at the raised beaches of Mormolo, Uche, Pima, Punta das Vacas and Morro do Sombreiro. ESA artifacts have been found in varying quantities across these localities, suggesting a range of occupations. Unfortunately, the lithic collections stored at the museum remain largely unpublished but they are currently under reappraisal [173].

At Dungo, Stone Age artifacts were first reported by geologists M. Neto [174–176], S. de Carvalho [167] and M. Feio [166], and initially it was J.D. Clark [132] and C. Ervedosa [177] that conducted studies on the surface lithic scatters (the site was published then as “Baía Farta”). The first excavations focused on an undated MSA site at Dungo III, but from the late 90s and in the context of a new collaboration with Sorbonne University, new excavations began to focus on the ESA deposits [153, 154]. The same team also discovered the site of Dungo V, where a stranded cetacean (*Balaneoptera* sp.) was discovered on the margins of a palaeo-lagoon estimated to be c. 1 Ma by biochronology and >350 ka by ^234^U/^230^Th on oyster shell remains [155, 178]. The articulated carcass spread across 6m and was associated with 57 lithics rich in flakes and pebble tools, among which several presented use-wear consistent with butchery activities [34, 178–180]. Consequently, Dungo V presents most of the patterns that could be used to argue for a single carcass site [see patterns proposed by 174, 175, 181], suggesting there is early evidence for coastal resource acquisition by hominins despite that dating efforts have proven difficult.

### The Dungo IV excavation and dating program

Fieldwork at Dungo IV began in 1994 under the direction of M. Gutierrez [155]. After digging nine test pits of various sizes to understand the stratigraphy and to assess the level of site disturbance, an extensive planimetric excavation was initiated in the 10x10 m sector M10, which was subsequently extended northward to sectors L10 and K10 (Fig 3A). In total, 300 m^2^ were excavated but only the M10 and K10 sectors were excavated down to the level of the marine pebbles/cobbles and marine conglomerate, which are highly variable in depth. The excavation was carried out by following artifact inclinations in order to follow the archaeological levels. This method was chosen because it was not possible to excavate following the sedimentary sub-units of the Red Sand unit due to: one, the deposit thickness, which is not compatible with fine excavation methods (the sedimentary units are too thick and cover a long time period), and two, the difficulty in identifying transitions between the sedimentary sub-units (transition between sub-units appears gradual in many parts of the excavations). All the material was recorded manually on an orthonormal plane (x, y, z) and each piece was plotted on millimetric paper. All the original fieldwork archives are stored at the Museu Nacional de Arqueologia de Benguela. However, there was no systematic sieving of sediment. Thus, we cannot access the finest lithic fraction, which is often invisible to the naked eye. During excavations, four beds of objects were identified, forming the four archaeological levels of Dungo IV. Concurrently, four different sedimentary sub-units were distinguished among the Red Sand unit [153]. Lithic artifacts were predominantly located in the sedimentary sub-unit “red matrix sand gravel” (Fig 4).

The lithic industry found during the excavation contrasts notably with the observations made on the surface assemblages by Clark [132] and Ervedosa [177], who emphasized diversity in the Large Cutting Tools (LCTs; defined as >10cm tools made by shaping or retouch and presenting at least one cutting edge) made from large quartz flakes (such as typical handaxes and cleavers). In fact, it is a quartz pebble/cobble-based lithic industry among which LCTs appear to be rare [153]. In this context, it has been suggested that Dungo IV was a pre-Acheulean site. This techno-cultural attribution was preliminarily dated by cosmogenic nuclides (^10^Be/^26^Al), providing burial ages ranging from c. 0.7 to 2.1 Ma, but these ages were not consistent with the geological context of the site. As a new technique, cosmogenic nuclide burial dating is proving to be extremely valuable for understanding geochronological and landscape evolution issues at sites across southern Africa [183–187].

In 2019 new burial duration constraints were obtained directly on the two stratigraphic units identified at the site: the “Red Sand unit” in which lithic artifacts occur and the “Paleobeach formation” which includes both the marine conglomerate and the sandstone bed. The Red Sand unit (Fig 4B) is divided into four main sub-units based on gravel size. The samples have been taken where the sandy deposit is the deepest, namely the “*Ravina*”. The “*Ravina*” area in sector M10 is a pluri-metric deep gully cutting within the Paleobeach formation, filled by the Red Sand unit and within which many artifacts have been recorded (as “*M10-Ravina*”) by the excavation team. Consequently, in the “*Ravina”*, the stratigraphic sequence exceeds 4 m of graded bedding (Fig 4B). The cosmogenic nuclide study, using the depth-profile technique on the two sedimentary units, provided an age bracketed between 614.5 ± 9.5 ka to 662.05 ± 10.24 ka [156] (Fig 4), suggesting Dungo IV is contemporary with the later Acheulean prevailing elsewhere in southern Africa since ca.1 Ma [90]. This age bracket also points towards rapid deposition of the Red Sand unit. The burial durations were modeled based on [188, 189]. In the minimum burial duration model, no cosmogenic nuclide production was considered after the burial of the deposits, whereas for the maximum burial duration model, the opposite was assumed. The preliminary burial durations obtained in 2010 for the four dated stone tools from the “*Ravina*” were confirmed [154, 156]. The significantly different burial durations and computed pre-burial denudation rates may imply a different history for the sampled lithics and the sediments. The proposed site formation hypotheses were either that there were diverse sources for the four quartz lithics, and/or that they each reflect different periods from the Early to Middle Pleistocene [156], which may be explained by the mixing of *in situ* lithic workshop remains with those lithics carried by the river within the Red Sand unit.

### Context of the red sand unit

In 2019 and 2022, the Red Sand unit at Dungo IV was re-investigated by authors attempting to identify the exact location of the archaeological layers within the four sedimentary sub-units, and to document the four different archaeological layers previously identified by the excavation team (between 1994 and 2015) (Fig 3). The Dungo IV profile is predominated by the “red sandy matrix fine gravel” (millimetric gravel size only), from top to bottom. It overlies the “red gravel to microconglomerate” sub-unit, comprising a high quantity of centimetric gravels among which notably more are quartz. The two upper sub-units, namely the “fine red sand” and the “fine gravely red sand” are largely eroded and not present in all profiles—especially in the western part of the excavation area. The “red gravel to microconglomerate” sub-unit is highly variable in thickness and inclination, creating an irregular topography possibly explained by water disturbance and/or, the irregular topography of the underlying Paleobeach unit; such inclinations are consistent with the inclinations of the lithics. We also documented a layer of marine pebbles/cobbles deposited on the marine conglomerate, but which is irregular and discontinuous (see West Profile in Sector L in Fig 3). In some areas, sporadic pebbles and cobbles present a surface crust made of the marine conglomerate suggesting the deposit remained under water prior to a marine regression. Consequently, on some parts of the site, the Red Sand unit is directly in contact with the marine conglomerate. Due to the absence of archaeological artifacts among these pebbles/cobbles and it being of marine origin [157, 169], we consider this deposit to be part of the Paleobeach formation along with the marine conglomerate. One main archaeological layer has been identified in the exposed profiles of the different sectors and it is encountered across the transition from the “red sandy matrix fine gravel” to “red gravels to microconglomerate” sub-units (Fig 3A). Some artifacts occur in the middle part of the “red sandy matrix fine gravel” and/or in the upper part of the “red gravels to microconglomerate”, but they are much scarcer and more scattered in comparison with artifact densities in the main layer. The transition between these two Red Sand sub-units is sometimes very difficult to assess due to the gradation of the sediments. The main archaeological layer occurs from 0 cm to 20 cm before the “red gravels to microconglomerate” (Fig 3A). Fig 3C shows the position of the main archaeological layer on top of the “red gravels to microconglomerate”. These observations suggest that the main archaeological layer cannot be easily associated to one sedimentary sub-unit of the Red Sand unit, even though it is primarily encountered at the base of the “red sandy matrix fine gravel”. It is identified by a very thin bed of lithics, mainly in quartz. The archaeological layer at times being very inclined (see “West Profile in Sector L10” in Fig 3A), it impacted the altitude of the archaeological artifacts–considerably across a 30 m long excavation area. Overall, these stratigraphic and contextual observations beg the question, are there four distinct archaeological layers at Dungo IV? The exposed profiles (30 m long and 10 m width) preserve only a single archaeological layer with variable topography. In this regard, we propose two hypotheses: one, the main archaeological layer that is visible in all profiles may be bracketed by much smaller accumulations of lithics that are restricted in horizontal space, and two, the main archaeological layer has been recorded as four different layers due to its high topographical irregularity, which may have been misleading during planimetric excavations. Only new excavations can provide clearer answers to these questions.

## Method

### The Dungo IV assemblage

The entire lithic assemblage is stored at the Museu Nacional de Arqueologia de Benguela and it comprises 2572 artifacts (without sieving). Table 1 presents a techno-typological inventory per layer. We opted to study the artifacts from all four archaeological layers together due to the low number of artifacts per layer, the tight chronological bracket, and the revised stratigraphic descriptions presented above.

**Table 1:** Artifact inventory per layer and per techno-typological category.

	Layer 1	Layer 2	Layer 3	Layer 4	Total (n)	Total (%)
Flakes	270	895	502	359	2026	78.9
Cores	31	29	41	73	174	6.8
Bifacial tools (unifacial and bifacial shaping)	4	5	6	8	23	0.9
Pebble/cobble tools	20	25	28	40	113	4.4
Unmodified tools	1	2	0	10	13	0.5
Polyhedra (multifacial shaping)	3	5	2	11	21	0.8
Tested cobble/pebbles	6	38	7	71	122	4.7
Undetermined	7	48	8	15	78	3.0
Total	344	1047	594	587	2572	100.0

### Technological approach

All artifacts have been described according to raw material types and states of surface preservation or modification (e.g. patina, fresh or smooth arrises, breakage patterns, macro-use-wear, such as percussion wear); refitting analysis has also been conducted on the assemblage [190]. For every flake (retouched or not), we recorded dorsal patterns (number of scars and orientation), butt typology, distal and cross-section morphology, and measurements according to both technological and morphological axes—relying on the nomenclature provided by Inizan et al. [191]. All the shaped tools (unifacial, bifacial and multifacial, which includes the polyhedral based on [192, 193]) were measured in their morphological orientation and we recorded the number of removal ‘sequences’ [see definition in 182, 183]. We identified blank types when possible (pebble, cobble, slab, block, split, cortical flake, undiagnostic non-cortical flake etc.) and the main areas which have been shaped (distal, lateral, proximo-lateral etc.). A similar set of attributes has been recorded for pebble and cobble tools and has already been partially published [169]. Cores were measured according to their technological axis [191] and reduction intensity was evaluated through the number of flaking sequences (definition of sequence based on [194]) and the number of removals per sequence. Core blank was identified when possible and all cores were grouped according to reduction strategies (e.g. orientation of removals and management of the blank volumetric patterns), but also according to the techno-morphology of the flakes which have been detached (e.g. elongated, quadrangular, convergent-edges, plano-convex) and/or those that were established during ‘physical’ or ‘mental’ refittings [195–197]. Finally, unmodified percussive tools presenting macro-use wear patterns [198–200] were measured and weighed. In addition, tested cobbles (presenting only two removals or less) were only measured. Table 2 provides basic metrics for each of these broad techno-typological categories per layer and highlights the close metrics among each group and each layer.

**Table 2:** Metric data (minimal, mean, maximal for length, width and thickness in centimeters) for each techno-typological category, per layer. Standard deviation is provided for all layers.

		Layer 1			Layer 2			Layer 3			Layer 4			All layers	
		Min.	$\bar{\text{X}}$	Max.	Min.	$\bar{\text{X}}$	Max.	Min.	$\bar{\text{X}}$	Max.	Min.	$\bar{\text{X}}$	Max.	$\bar{\text{X}}$	s.d.
Flakes	L.	1.0	3.59	11.4	1.1	3.52	10.5	0.6	3.81	10.5	1.0	3.97	10.5	3.72	1.62
	w.	0.5	3.13	14.1	1.0	3.03	11.4	1.0	3.31	10.6	1.0	3.43	10.6	3.21	1.44
	t.	0.4	1.20	5.4	0.2	1.29	7.1	0.3	1.34	5.2	0.3	1.45	5.3	1.34	0.81
Cores	L.	2.3	6.60	14.4	2.2	5.85	11.0	3.5	6.45	11.2	3.0	7.53	17.1	6.79	2.41
	w.	3.3	6.89	15.4	1.7	5.70	12.7	2.9	5.80	11.9	2.6	8.25	13.4	7.01	2.80
	t.	1.9	5.48	10.4	1.6	4.41	9.6	1.6	4,64	10.5	1.4	7.24	16.4	5.76	2.78
Shaped tools (unifacial and bifacial)	L.	10.1	12.85	18.1	6.4	9.14	10.6	6.9	9.67	16.0	5.6	10.10	16.9	10.11	3.27
	w.	7.0	7.80	8,3	4.7	5.27	6.6	4.7	6.30	8.2	4.4	6.71	8.9	6.77	1.43
	t.	3.0	4.50	6.3	2.2	4.30	5.5	3.3	4.42	6.5	2.4	4.02	5.4	4.21	1.04
Pebble/cobble tools	L.	3.0	6.50	10.9	2.6	5.88	11.0	2.9	6.94	12.2	3.2	6.93	13.6	6.82	2.50
	w.	3.0	6.73	10.6	2.7	5.50	10.0	2.8	6.61	11.1	3.5	6.84	13.9	6.70	2.32
	t.	1.6	4.04	7.5	1.9	3.80	5.5	1.5	4.58	8.7	1.9	4.52	8.5	4.38	1.48
Unmodified tools	L.	9.8	-	-	5.8	6.05	6.3	-	-	-	7.2	10.10	16.1	9.98	2.99
	w.	9.5	-	-	4.4	4.85	5.3	-	-	-	5.6	8.62	11.2	8.15	2.18
	t.	9.0	-	-	2.7	2.90	3.1	-	-	-	4.9	7.62	10.4	6.57	2.29
Polyhedra (multifacial shaping)	L.	5.6	8.10	11.7	2.2	4.06	6.0	5.4	6.15	6.9	5.0	7.42	8.8	6.94	2.18
	w.	4.5	6.80	11.1	2.4	3.70	4.9	5.2	5.90	6.6	3.6	6.90	8.0	6.05	2.21
	t.	3.6	6.17	11.1	2.4	3.70	4.9	4.6	5.00	5.4	3.0	6.08	7.5	5.55	2.27
Tested pebbles/cobbles	L.	3.8	9.53	15.6	4.2	7.53	18.0	3.9	10.01	23.6	4.0	10.01	25.8	8.96	3.32
	w.	4.1	8.43	10.2	3.5	5.88	10.6	3.0	8.24	20.8	3.4	8.24	19.7	7.27	2.71
	t.	3.4	6.78	9.6	2.1	4.32	10.5	3.5	6.81	16.0	1.7	6.35	18.1	5.63	2.48
Undetermined	L.	2.2	3.50	5.7	1.5	3.30	7.0	2.2	3.73	6.9	1.2	3.96	11.0	3.77	2.19
	w.	1.5	2.60	4.3	0.5	2.21	4.3	1.3	2.87	4.6	1.0	2.81	5.4	2.64	1.47
	t.	0.7	1.98	4.2	0.2	1.46	4.2	1.0	1.75	2.4	0.4	1.95	4.2	1.79	1.24

In addition, each core, pebble/cobble tool, shaped tool and retouched flake has been studied in detail using systematic diacritical sketches [201, 202]. These provide a chronology for removals and their relation to the volumetric patterns of the blank, and also, detail for a techno-functional analysis. The techno-functional analysis was based on Boëda [203] and as applied by several others [204–209], which highlights the value of this approach when understanding stone tools with limited removals (e.g. pebble/cobble tools) or very short reduction sequences [210–214]. This approach is also useful when analyzing complex shaped tools [215–218]. The approach includes an evaluation of technological determinism inherent in each object, meaning, what can be achieved mechanically with the object. It is evaluated using volumetric patterns (e.g. the weight, overall shape, cross section morphology or the way the volume is distributed across the object) and the location of the cutting edges, the pointed tips or percussive surfaces. Both volumetric patterns and the location of active parts are believed to have an influence on the prehensile part to the artifact. Specific focus is put on the cutting edges to identify where, along the cutting edge, the morphology is changing (e.g. from a sinuous delineation to rectilinear delineation). The study of these patterns usually results in a flexible classification highly adapted to each site’s assemblage, but the approach makes inter-site comparisons more difficult. In this paper, we describe the tool categories using only the following techno-functional descriptive patterns:

- orientation of cutting edges in relation to the tool blank volume (tool blank volume is considered here as the “*Unité Techno-Fonctionelle transmettrice*”: *UTFt*): distal, disto-lateral, lateral, proximal regarding the longitudinal axis.
- technology and morphology of potential prehensile parts (“*Unité Techno-Fonctionelle prehensible*”: *UTFp*): for examples, cortical convex surface or a lateral back (see definition in [216]).
- technology and morphology of potentially active parts (“*Unité Techno-Fonctionelle active*”: *UTFa*): angle value of cutting edges (± 2.5°); morphology of cutting edges and points (delineation and cross-section).

This methodology is based on the idea that the three parts act simultaneously in tool use: *UTFa* is activated thanks to *UTFp* and a great role is played by the overall size, weight and morphology of *UTFt* for the “*functionality*” of the tool. In the techno-functional approach, “*functionality*” is different from “*function*” because the former refers to all the ways a tool can be used, whereas “*function*” refers to the specific use of an artifact that can only truly be known when applying use-wear analysis. All these recorded data are considered in light of the reduction sequence that the tools were created by. Consequently, with this approach, stone tools which have been manufactured in different ways may present similar “*functional potentials”*. This means that due to similarities in volumetric configurations, different tools may be used in similar ways.

### Inter-site morphometric comparisons

A supplementary corpus was also created for comparative purposes. It comprises the basic metrics (length, maximal width, and maximal thickness) of shaped tools measured by the authors, along with summarized bibliographic data from several contemporary or sub-contemporary coastal ESA sites from southern Africa. It includes shaped tools, mainly LCTs (>10cm) from Elandsfontein and Cape Hangklip (data from the online database of [207]), and artifacts from Penhill Farm, Namib IV and Gemsbok, also measured by the authors. Information regarding the sites and measurements are provided in Table 3 and S2 Table, and site locations are illustrated in Fig 1. In this paper, we also provide a traditional morphometric comparison of bifacial/unifacial shaped tools between these sites to compare differences in their shape. The basic metrics have been used to calculate the following indices for comparison: elongation (Length/width), fineness indice 1 (Length/maximal thickness) and fineness indice 2 (maximal width/maximal thickness). Differences between raw measurements and the indices are explored using ANOVA (ANalysis Of VAriance) and Tukey HSD Tests, where p-values >0.05 were considered significant (see similar application and detailed methodology described by [162, 208]). All the analyses were conducted using R Studio, version 1.4.1106 [224]. The primary aim of this inter-site comparison is to provide some insight on the differences and similarities between ESA coastal sites contemporary with the MPT in southern Africa. However, due to varying methods of technological analyses for the lithic collections [225, 228], the quantitative approach was favored for this study. We consider it to be the most efficient as it provides standardized length, width and thickness data for shaped tools, irrespective of the different lithic approaches. In contrast, comparing the reduction strategies of retouched flakes is harder due to the varying ways of reading artifacts and classifying them.

**Table 3:** Details and bibliography used for the lithic collections from Middle Pleistocene transition Earlier Stone Age sites from the southern African coastal plain: Namib IV, Gemsbok, Elandsfontein, Cape Hangklip and Penhill Farm.

Site name	Location	Number of artifacts	Number of LCTs considered in this study	Storage location	Age	Dating method	Context	Main references
Namib IV	Central Namib, Namibia	396	9	National Museum of Namibia	400–700 ka	Biochronology and ESR	Deflated surface sand dune surface	[182, 212–214]
Gemsbok	Sperrgebiet, Namibia	1166	111	National Museum of Namibia	400–800 ka	Geology, biochronology and typochronology	Mining trenches within raised beach	[59, 61, 215, 216]
Elandsfontein (use of ‘Cutting 10’ data)	Western Cape, South Africa	-	286	Iziko Museum	>780 ka	Paleomagnetism and biochronology	Excavation and surface within paleo-sand dunes	[57, 58, 207, 217–223]
Cape Hangklip	Western Cape, South Africa	-	268	Iziko Museum	400–700 ka	Geology and typochronology	Excavation and surface within raised beach	[116, 117, 207, 224–227]
Penhill Farm	Eastern Cape, South Africa	9904	42	Witwatersrand University	<1.37 Ma	^10^Be/^26^Al	Colluvial debris flow contained within an alluvial terrace	[66, 162, 228–236]

## Results

### Raw material distributions

Raw material distributions exhibit quartz-based lithic production for all techno-typological categories (Table 4). Only the non-knapped percussive tools present an important component of volcanic rocks [169], and the shaped tools present a more diverse suite of raw materials. We distinguish hyaline quartz, which is much more homogeneous in structure, from the broad quartz category, among which are classified ‘saccharoid’ and ‘milky’ quartz types [237–239].

**Table 4:** Raw material distribution per techno-typological category of Dungo IV.

	Quartz	Hyaline quartz	Quartzite	Chert	Volcanic	Undetermined	Sandstone	Total
Unretouched flakes	1225	448	141	88	32	7	9	1950
Cores	119	17	13	10	9	2	4	174
Tested cobble/pebbles	88	5	6	2	9	10	2	122
Pebble/cobble tools	74	8	11	6	9	2	3	113
Undetermined	49	17	4	5	-	3	-	78
Retouched flakes	48	16	8	5	1	-	1	79
Shaped tools	12	-	4	6	1	-	-	23
Polyhedra	18	1	2	-	-	-	-	21
Non-knapped tools	6	-	-	-	6	-	-	12
Total	1639	512	189	122	67	24	19	2572
%	63.8	19.9	7.3	4.8	2.6	0.9	0.7	100.0

### Refits and state of preservation

**Fig 5:**
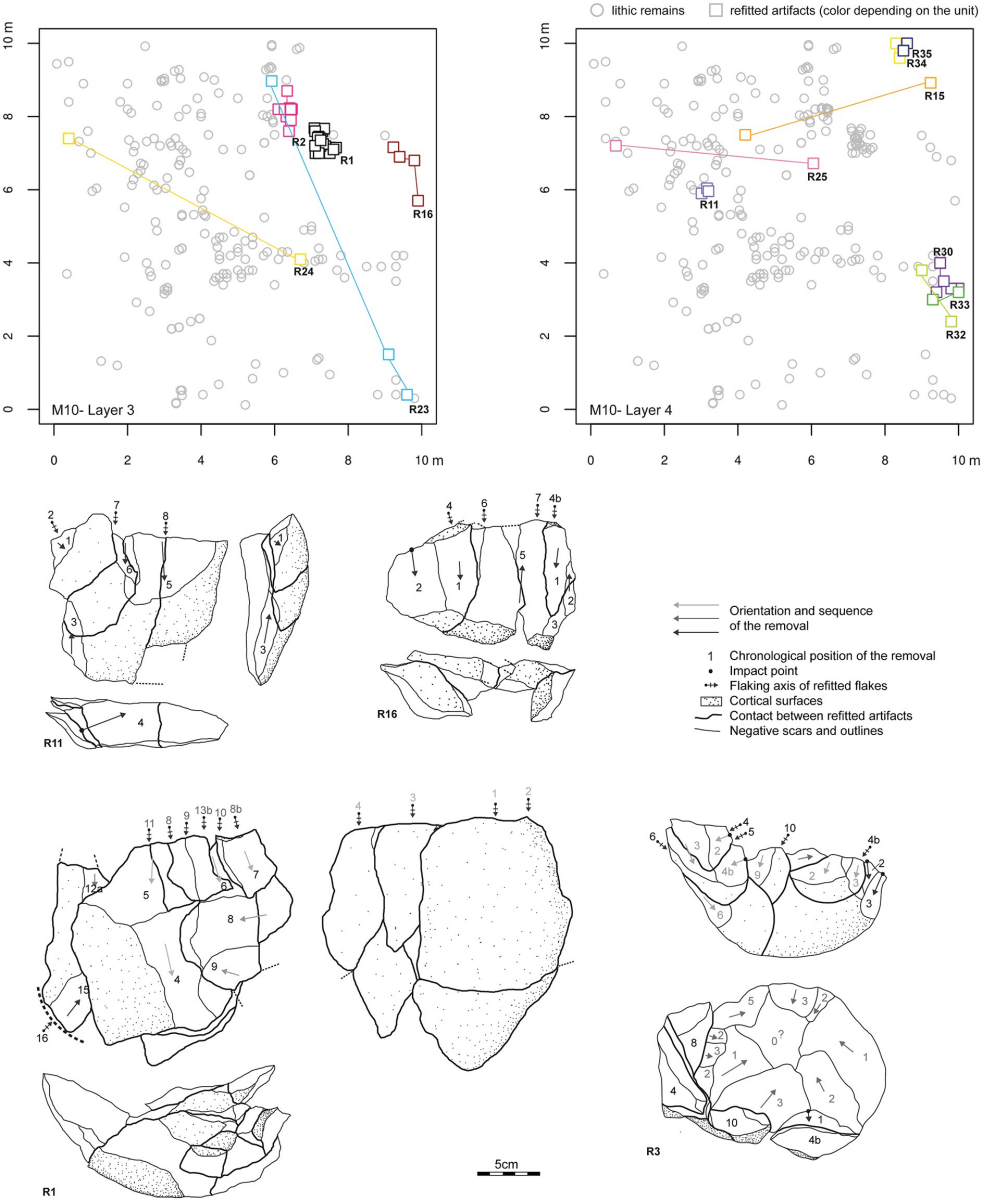
Lithic refit units R1, R3, R11 and R16 and planimetric mapping of the refittings of the sector M10 Layer 3 and Layer 4.

We refitted 188 individual artifacts that constituted 47 refit units. These included: cores and associated flakes (n = 6), two to 16 flakes without core (n = 36), tested cobble and first flake (n = 3), and naturally fragmented cobbles (n = 2). These refits were also used to describe the reduction strategies in the following section. Refitted artifacts were plotted on an orthonormal plan and several refits showed important concentrations (Fig 5), which suggested the knapping process occurred *in situ* with minor post-depositional disturbance.

However, the breakage rate is important and especially for flakes (n = 657), whereas cores (n = 4) or pebble/cobble tools (n = 3) are rarely fractured. This may be due to quartz breaking during knapping but also to trampling on-site during actual herders’ transhumance, which may have affected the finest pieces of the deposit. Indeed, we distinguished fractures which occurred during knapping, such as “Siret accident” with a fracture plan associated to the impact point of the ventral surface (n = 29), from pieces with two or more fractures (n = 66). We note that flakes present a transversal break (n = 295) or a longitudinal or sub-longitudinal breakage (n = 267). Another supporting observation for minimal assemblage disturbance is the overall freshness of the lithic arrises and cutting edges.

### Core reduction strategies

**Fig 6:**
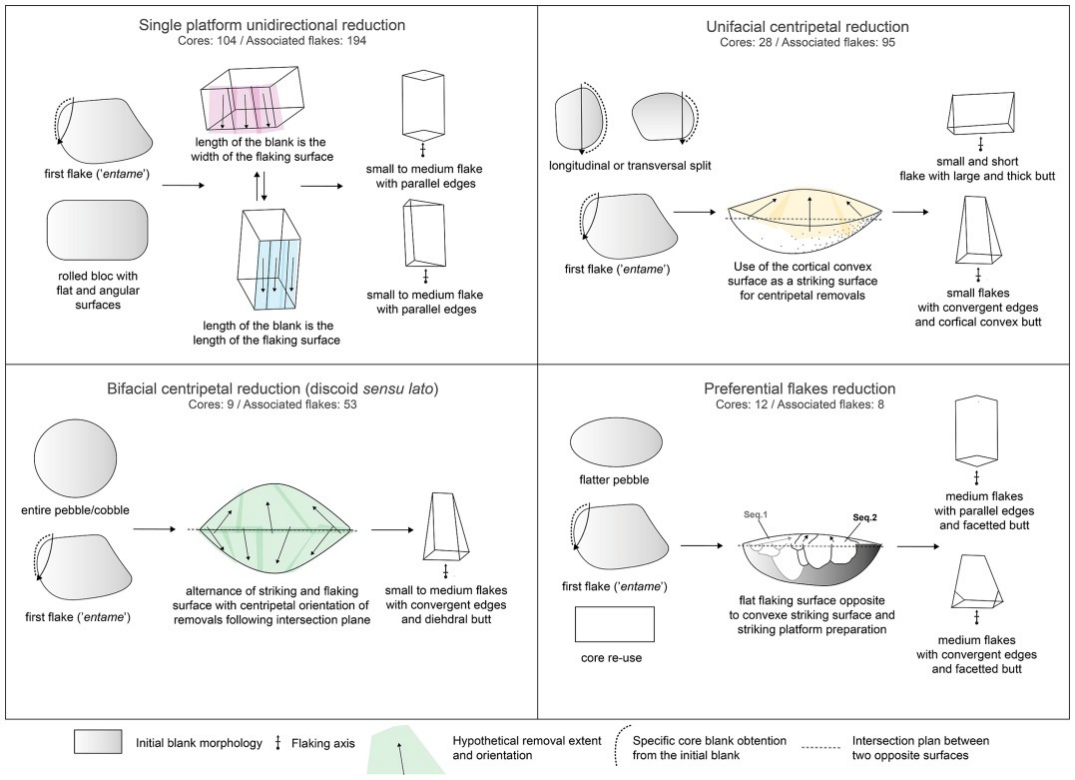
Sketches of the four main flake production strategies at Dungo IV and the techno-morphological patterns of the resulting flakes.

Here, we describe the four main reduction strategies that collectively account for 153 cores– 87.9% of the total core sample (Fig 6). The rest of the assemblage (n = 21) presents multidirectional informal cores. Their irregularity both in reduction sequence and morphology do not facilitate their inclusion in any relevant technological group.

**Fig 7:**
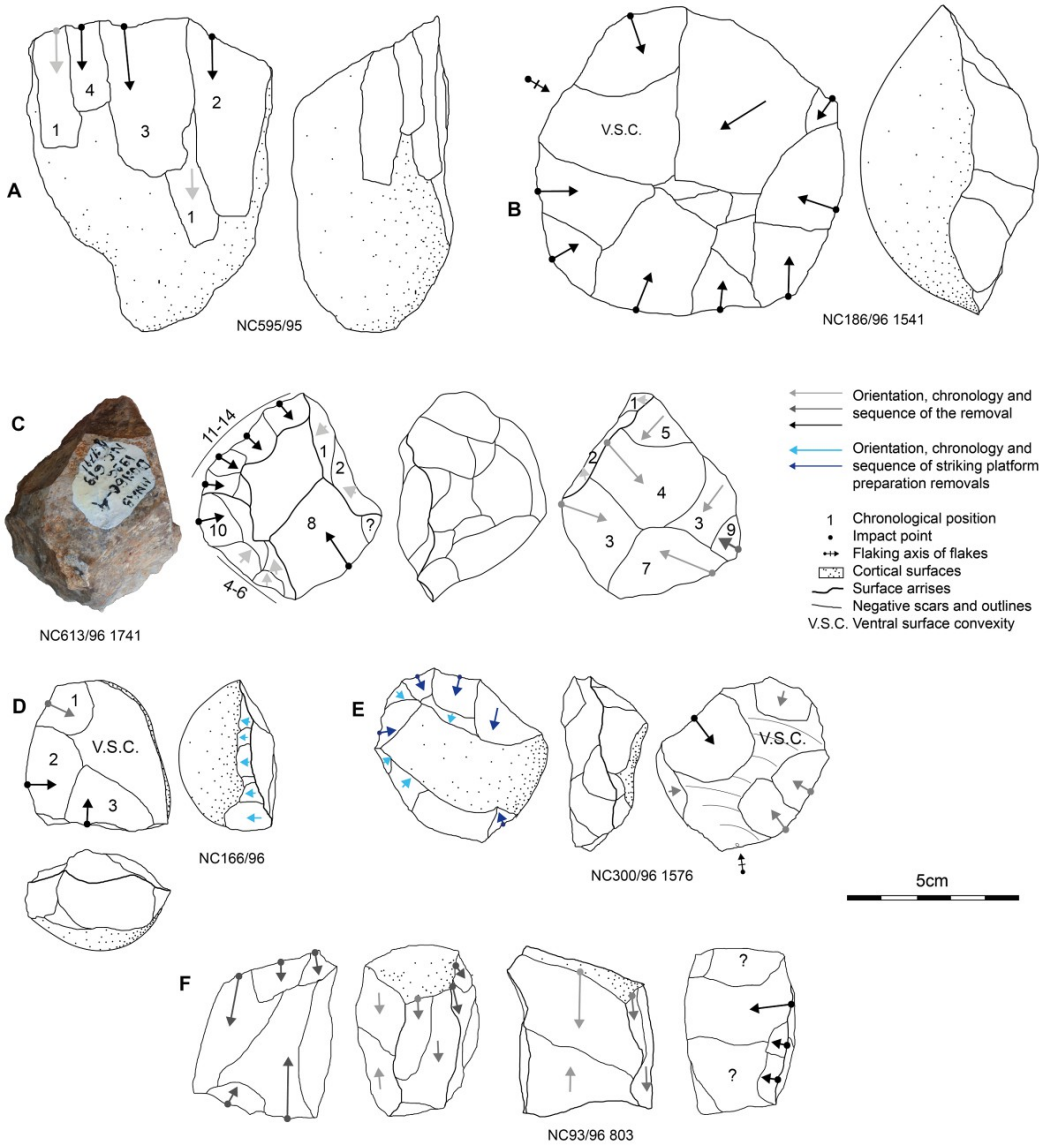
Cores from Dungo IV. A, B, D, E, F are quartz while C is chert.

The main reduction strategy was identified based on 104 cores (Fig 7A and 7F). It uses a single flat striking surface to produce quadrangular or elongated flakes via recurrent unidirectional removals. When the flake is detached from the lateral extremity of the flaking surface, knappers may obtain flakes with a cortical lateral back also called a “*débordant*” flake. All the products seem to be smaller than 10 cm and only two cores present one large flake negative (>10 cm based on [114, 240]). It also seems that these cores were exploited via short reduction sequences barely exploiting more than two contiguous surfaces. We observed an important trend in the use of rolled quartz blocks, rather than cobbles and/or pebbles, which may be related to the intention of exploiting flatter and angular cortical surfaces—the cortical surface of cobbles and pebbles being more rounded. These blocks are rarely encountered in the pebble and cobble outcrops, but on the contrary we did observe these blocks in the current Dungo IV site in the uppermost part of the river bed.

The unifacial centripetal reduction strategy (n = 28) (Fig 7B) is the second most common strategy. Blanks are predominantly produced using the split technique, across the longitudinal or transversal axis of a pebble [205, 241]. Alternatively, the first flake obtained from a knapped pebble/cobble could also serve as a suitable blank. The cortical surface is used as a convex striking surface to exploit the opposite surface. The removals may vary from short flakes to more elongated flakes depending on the flaking surface convexities, the depth of the striking platform and the overlapping of the removals. However, they will always present a cortical convex butt which accounts for the main thickness of the flake. The dorsal surface will be plain and convex (ventral surface of the core’s flake blank) or will present convergent to centripetal removals. This strategy seems to produce only small flakes (<5 cm).

The bifacial alternate flaking with centripetal modality (n = 9) is the third most common (Fig 7C). This strategy groups both the SSDA cores (“*Système de Surfaces de Débitage Alternées*” or “alternated flaking surfaces system”) [196] and discoidal *sensu lato* cores [242, 243]. SSDA cores are characterized by an alternance between flaking/striking from two opposite surfaces, which will help with recurrent flake production thanks to <90° angle maintenance. It is interesting to note that in this group, siliceous rocks are well-represented in the assemblage and when the blank is visible it represents small blocks unidentified in the vicinities of the site [169]. Small blocks of siliceous rocks may come from the primary deposit encountered at the foot of the End Rift Conformity 20 km inland [170, 244]. However, the initial blanks are mostly indeterminate as these are the most reduced cores and we can see up to four flaking sequences each with several removals. The alternating system or SSDA also allows for long continuous sequences of removals due to the automatic maintenance of favorable angles between the two opposite surfaces and which can lead to discoidal core organization. A typical flake of this production type presents convergent edges with convergent to centripetal dorsal patterns, and a plain or dihedral butt.

Finally, we identified several cores (n = 12) which present a similar volumetric configuration: two opposite asymmetrical surfaces. The flatter surface is used as a flaking surface and the more convex surface serves as the striking surface to obtain a thin flake with a linear peripheral cutting edge. The striking platform is systematically prepared by small removals (Fig 7D and 7E). These prepared cores refer to a hierarchized reduction strategy [185, 245]. The variability is contained in the blank production and preparation. Knappers used both the natural and initial blank volumetric patterns when convexities were suitable for this strategy, or they re-used a core already exploited via another strategy. Consequently, dorsal patterns and outline morphology of the flake are highly variable but the butt always presents the stigmata of striking platform preparation. Indeed, one can consider these cores (n = 12) as partially prepared cores to obtain a preferential flake.

### Techno-typological flake patterns

**Fig 8:**
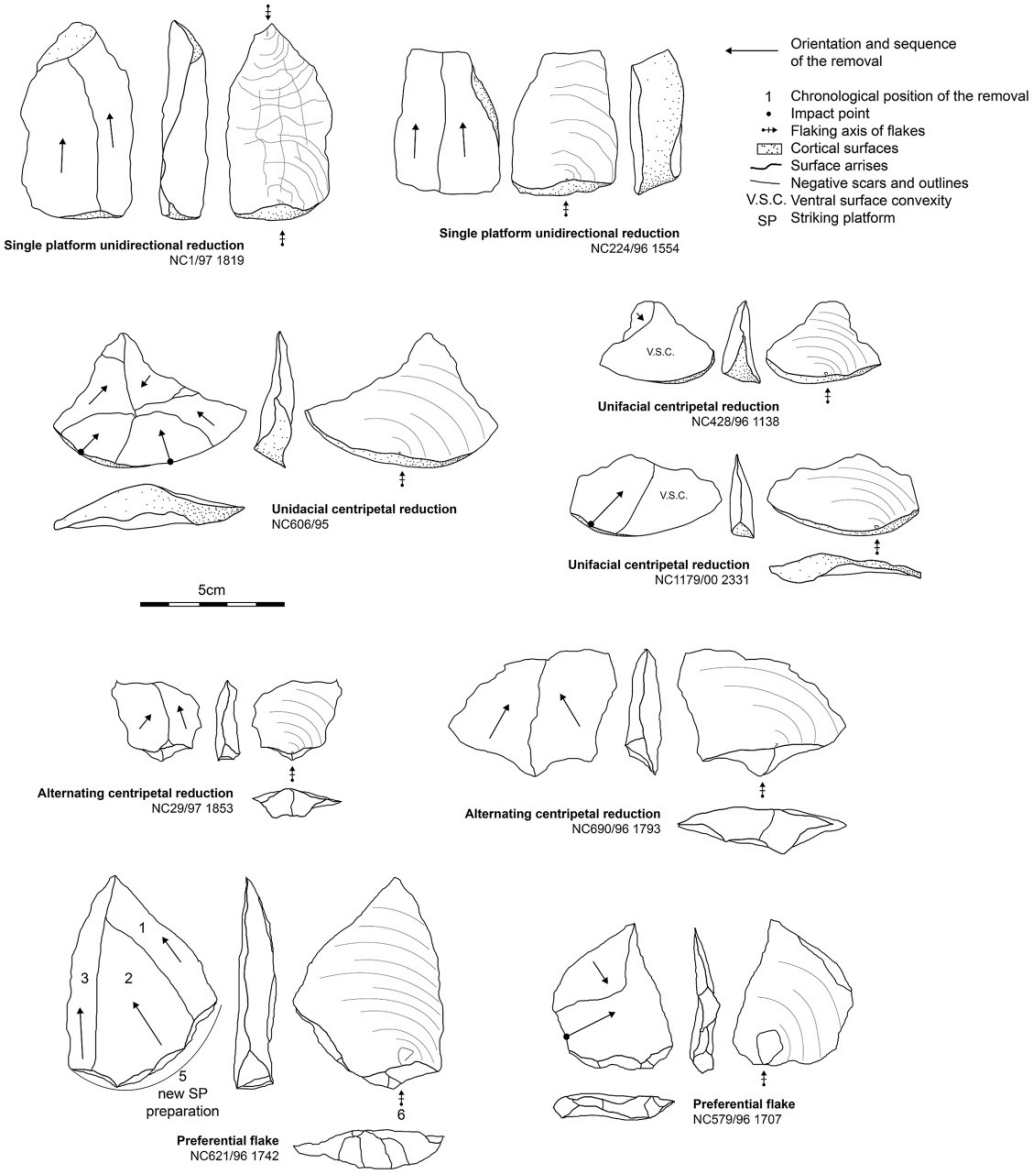
Quartz flakes from Dungo IV and their association with specific core reduction strategies.

Relying only on the entire flake assemblage and its techno-typological attributes, we observe consistency with the core assemblage described above (Fig 8).

Due to the poor core preparation and the use of whole gravels, we used Toth’s cortical flake classification ([246] which provides the reduction sequence position of flakes thanks to cortical residual surfaces (across both platforms and dorsal surfaces). We observe a high predominance of flakes presenting both cortical butts and cortical dorsal surfaces (n = 933) and flakes presenting a non-cortical butt associated with a dorsal cortical surface (n = 366). Only 658 flakes show a total absence of cortical surfaces. This fact, along with the importance of cortical or partially cortical butts, confirms the use of entire gravels as blanks and the frequent use of cortical striking surfaces coupled with a scarcity of flaking surface preparation.

When readable, the dorsal scars present mainly uni- or bi-directional orientations (n = 910), orthogonal (n = 214) and multidirectional (n = 170). The scarcity of convergent (n = 88) and centripetal (n = 49) scars is surprising, particularly given the presence of reduction strategies in the core sample that would facilitate such flakes (i.e., discoidal). This scarcity can likely be explained by the poorly overlapping removals we observed in unifacial centripetal reduction schemes, and the shortness of reduction sequences. Overall, the flake assemblage provides evidence that is consistent with the dominance of a single platform unidirectional flaking strategy as exhibited by the cores. Focusing on the butt types, we observed a predominance for entirely cortical butts (n = 890), followed by plain flat butts (n = 775), but facetted (n = 18) or dihedral (n = 111) butts are rarer.

Regarding the overall morphological patterns of the flake assemblage, we observed a primary trend for producing small flakes (<5 cm), and only 58 flakes present lengths greater than 7.5 cm, among which nine present lengths between 10 and 14 cm; only two flakes present widths between 10 and 13 cm. This scarcity of large flakes echoes the core metrics and the flake scar negatives on the cores. In addition, we also identified only two cores that presented the removal of large flakes and both belong to the single platform unidirectional reduction strategy.

### Flake tools

The 79 retouched flakes are predominated by quartz artifacts retouched in highly variable ways, measuring between 2.7 and 11.5 cm with a mean morphological length of 5.4 cm. Long retouch removals predominate and among them we observe a higher frequency of abrupt removals compared to low-angle retouch (Table 5). Unifacial retouch is most common and mixed retouch (meaning a retouched area presents unifacial retouch along with a few bifacial retouch removals as well) is also frequent. These tools are primarily retouched on the cutting edges (rectilinear = 30; convex = 25; concave = 5), but also on pointed extremities (n = 11) or denticulated edges (n = 13). We observed only one retouch sequence along a tool edge, suggesting the absence or general rarity of cutting-edge re-sharpening—the latter usually being identified by several generations of retouch. Generally, the retouched area can extend inwards along the edge from 1.5 to 5.5 cm. Trends related to artifact size, where LCTs are almost absent (3 artifacts are over 10cm long), illustrate the small to medium-size range of retouched flakes from Dungo IV. In addition, no flake tool exhibits percussive or macro-wear damage/modification, pointing toward a light tool component for retouched flakes.

**Table 5:** Distribution of retouch types (location, extent and orientation) at Dungo IV.

Retouch type		Mixed	Bifacial	Dorsal	Ventral	Total
Mixed	Abrupt	4	-	-	-	4
	Low-angle	2	-	-	-	2
Short	Mixed	5	3	2	-	10
	Abrupt	-	4	9	-	13
	Low-angle	-	3	1	-	4
Long	Mixed	3	1	-	-	4
	Abrupt	-	6	16	3	25
	Low-angle	-	4	4	5	13
Notch	Abrupt	-	-	2	2	4
Total		14	21	34	10	79

Among the 79 artifacts, we note that plano-convex tools (n = 29) and those presenting a lateral surface or lateral flat/back (n = 25) are well-represented.

**Fig 9:**
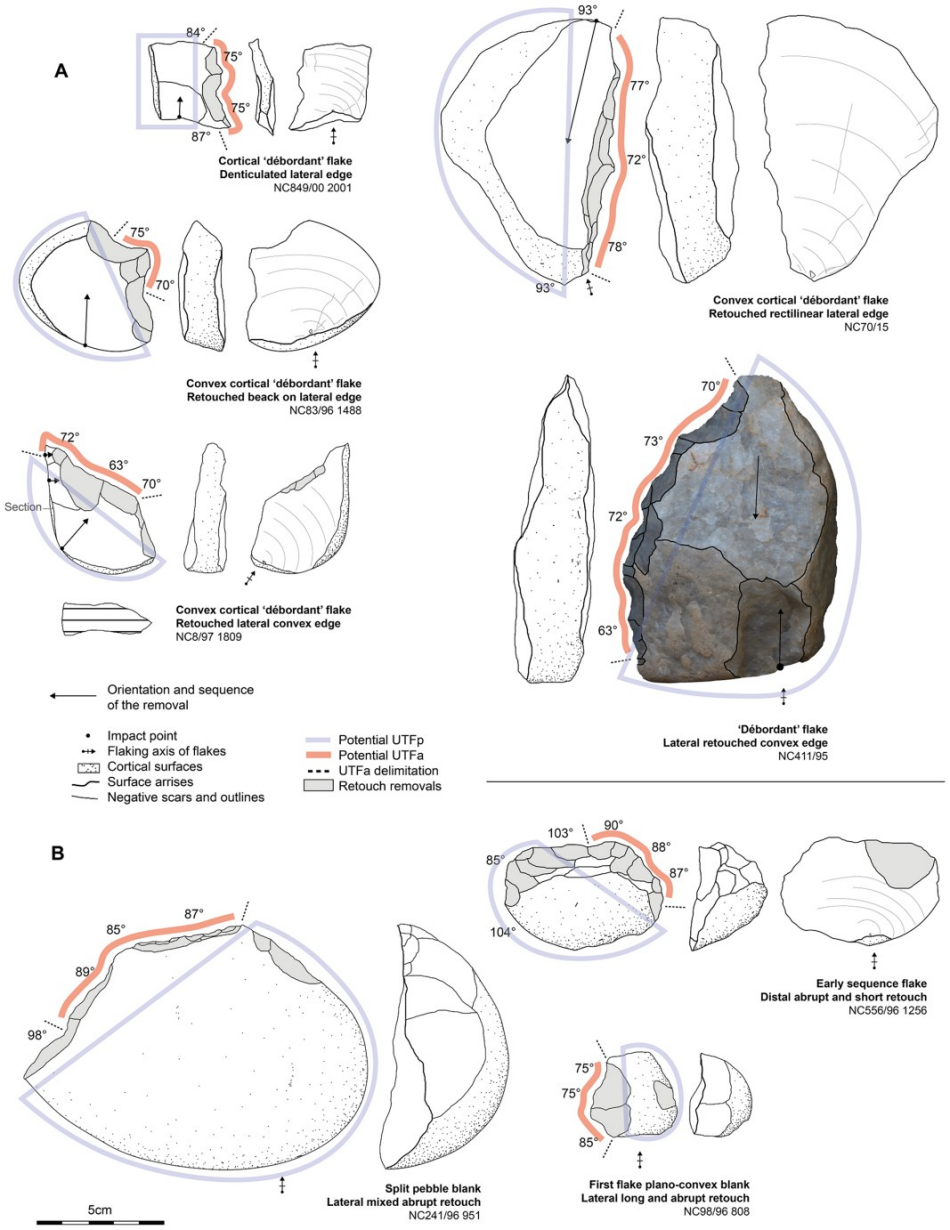
Examples of flake tools from Dungo IV. A: retouched flakes with lateral back interpreted as potentially prehensile part opposite to various retouched edges. B: plano-convex cortical flake blanks with abrupt retouch.

Plano-convex flake tool blanks are mainly cortical and so, have been obtained during early reduction sequences (n = 25). These cortical blanks are primarily obtained through the transversal split technique, and the use of this technique for the production of short plano-convex cortical blanks exemplifies the optimization of the natural cortical convexities of small quartz pebbles (Fig 9B). Other plano-convex tools (n = 4) obtained their plano-convex structure due to retouching. These tools primarily present a convex to rectilinear abruptly retouched cutting edge, varying in angle from 70° to 95°. It suggests an association between small to medium (3 to 8cm long) tool size and an overall robust structure, coupled with particularly thick cutting edges. This robust structure is due to the lithic mass being concentrated on the mesial dorsal part of the tool (within the convexity of the superior surface which act as a “mass reserve”).

Flake tools with a lateral back are obtained or modified through retouch, (n = 4) or through the initial production of ‘*débordant*’ flakes (n = 10), or from short flakes with a large and wide butt (n = 6) (Fig 9A). These are usually characterized by lateral or disto-lateral cutting or piercing type tools opposite to the lateral surface. Indeed, this lateral surface has been interpreted as a potential prehensile part—or at least, a surface which may facilitate the handling of the flake. Most of the time, this lateral surface accounts for the morphological length of the tool and varies from 2.7 to 11.4 cm—the flake tools >10 cm also have this lateral surface.

### Cobble/pebble tools

We distinguish pebbles (4–64 mm) from cobbles (64–256 mm) based on Wentworth’ scale [247]. At Dungo IV, pebbles occur more than cobbles for tool production and even though larger clasts are available, this trend seems to mimic the raw material properties in the local landscape [169]. Raw materials are primarily quartz, followed by quartzite and volcanic rocks (Table 4), and pebble/cobble tools numerically account for the main part of the toolkit. These have been interpreted as tools due to the presence of a homogeneous cutting edge (no sinuosity of the edge which would have to refer to SSDA flake extraction) associated with a series of removals (>2 removals) that intend to shape a cutting edge or a pointed extremity.

**Fig 10:**
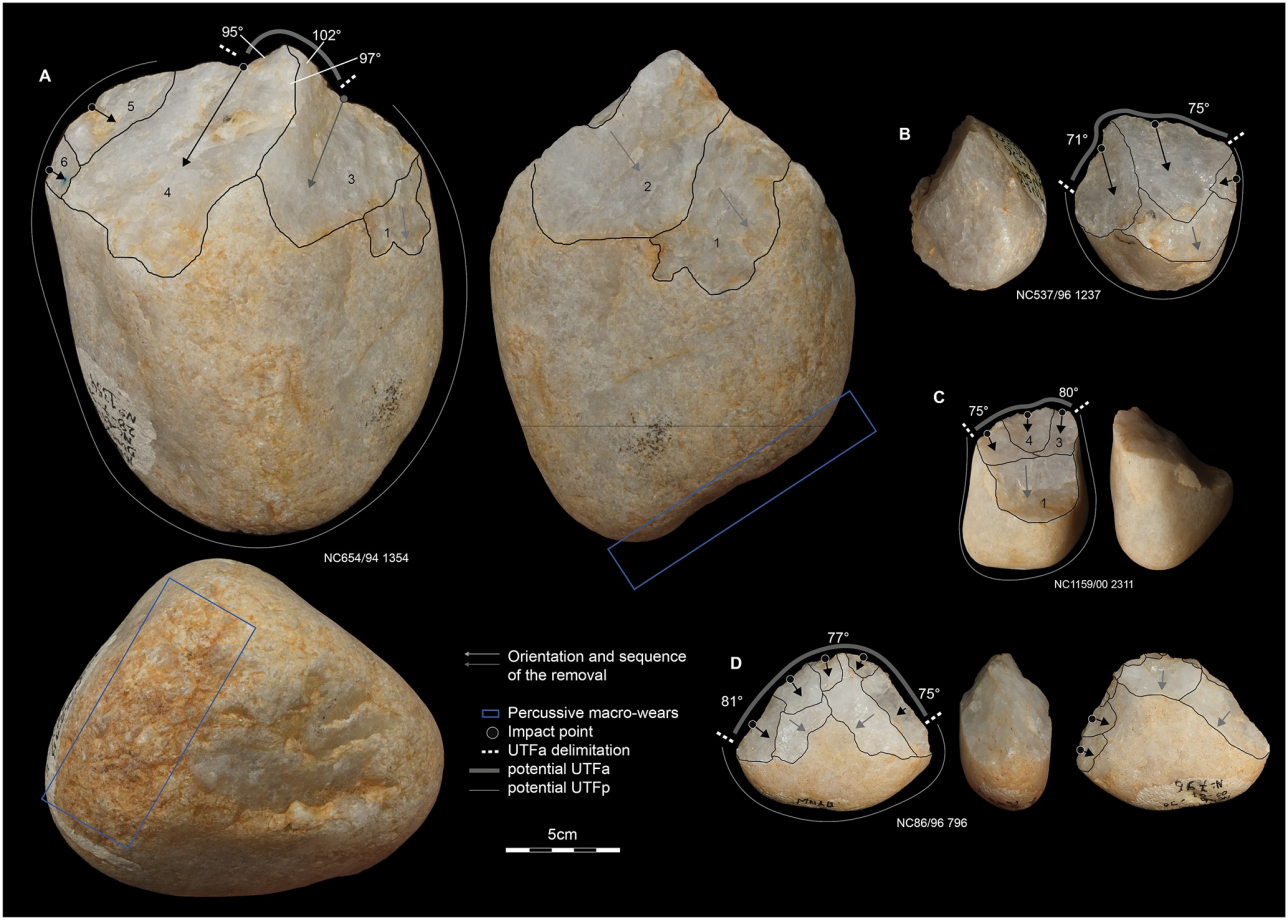
Examples of pebble/cobble tools from Dungo IV. A is a pick on a cobble presenting macro-wear patterns on the proximo-lateral cortical surface. B and C are micro-choppers whereas D is a micro-chopping-tool.

Most of them have been shaped distally or laterally using one to two generations of removals, either unifacial or bifacial [169]. This method creates a cutting edge from rounded blanks with variable angle values, ranging from 60 to 120° (mean = 88.4°; s.d. = 13.4). This reflects diversity in both cutting edge morphology and the distance between cutting edges and prehensile tool portions, but also suggests a trend toward robust (steeply-edged) cutting tools. These pebble/cobble tools are described and discussed in terms of lithology, morphometrics and technology in [169]. We present in this paper three specific patterns among this diversified cobble/pebble toolkit:

1. *Micro*-chopper/chopping tools (Fig 10B–10D) (n = 30): we consider here that producing <5 cm long tools on quartz pebbles is a characteristic feature of the assemblage. These tools only present linear, sinuous or convex cutting edges (with an absence of pointed tips). The mean angle of their cutting edges is <80° and consequently, thinner than the overall mean of cutting edges for the pebble/cobble tool component. The cutting edges of these *micro*-pebble tools may have been produced both by bifacial (chopping-tools) and unifacial removals (choppers). It proves the presence of a robust small tool component that associates naturally rounded and robust prehensile parts to a relatively thin cutting edge.
2. Cobble/pebble picks (Fig 10A) (n = 9): these mid-sized tools (5 to 10 cm long) are systematically obtained through trifacial distal shaping of a pebble with large removals, creating three adjacent surfaces. These three surfaces converge and create a distal pick trihedral to rhomboid in cross-section with robust angle values ranging from 80° to 100°. These present a higher length (mean = 7.37 cm; s.d. = 2.12) and maximal thickness (mean = 5.76; s.d. = 1.56) than the mean value of pebble/cobble tools (Table 2). This robust distal pointed part has been interpreted as the potentially active part and is associated with a robust proximal cortical rounded base which may have facilitated a powerful grip.
3. Distal platform cobble/pebble tools (n = 9): these too are mid-sized tools, presenting a distal surface obtained through centripetal removals that create a distal surface. The shaping plane is perpendicular or sub-perpendicular to the longitudinal axis of the blank and provides important angle values >90° for concavo-convex short cutting edges.

### Bifacial and unifacial shaping/reduction strategies

**Fig 11:**
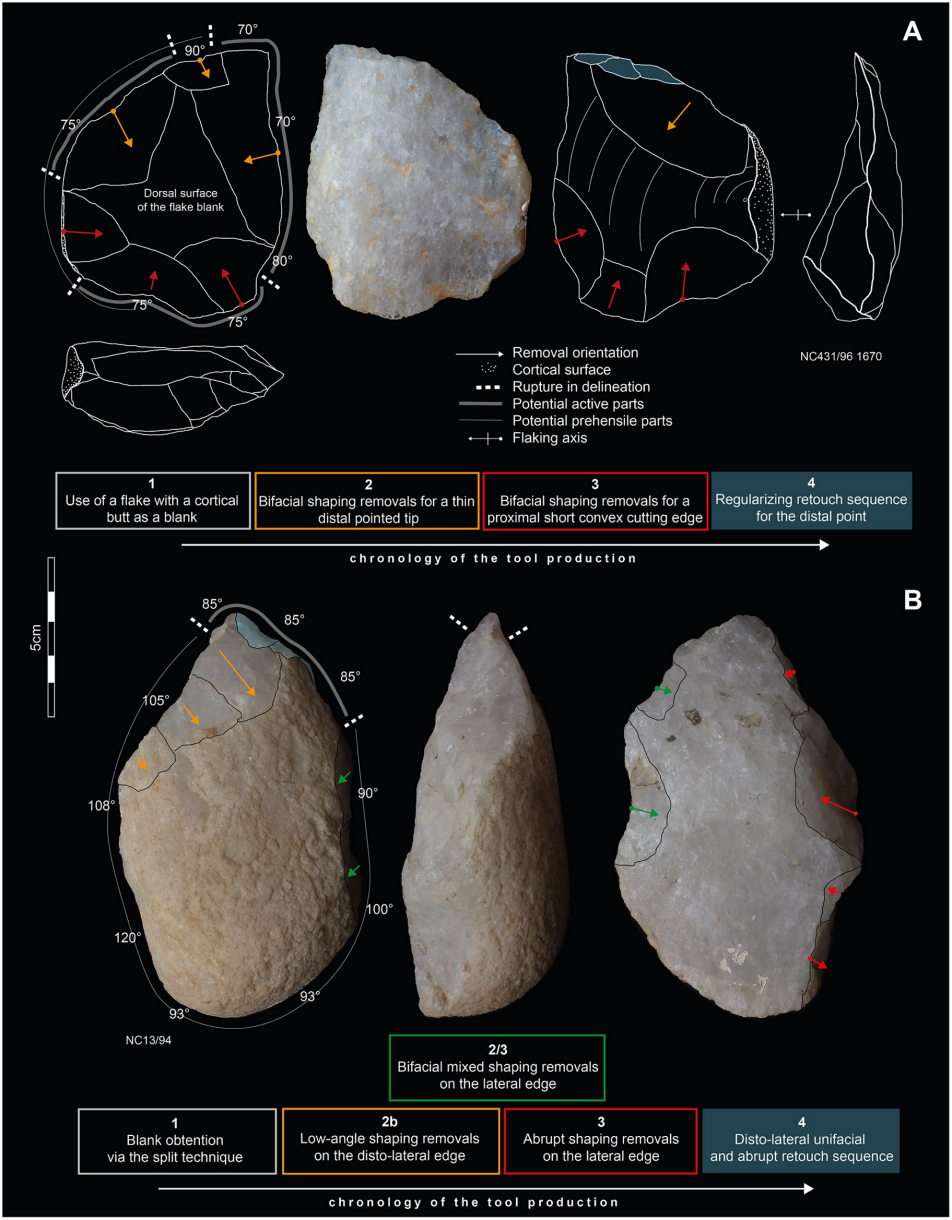
Examples of shaped flakes from strategy number three (A) and a longitudinally split cobble from strategy number two (B). The chronology of their production is explained under the photo, the colors of the box are related to the colors of the arrows or of the removals on the artifact.

From a production perspective, we distinguish three different shaping strategies based on 20 bifacially and three unifacially shaped tools (n = 23). Here, we integrate all the shaped tools, among which LCTs and much smaller shaped tools are included, and document the following strategies (see Table 2 for metrics):

- Strategy 1 (n = 9): the removal sequences continuously take into consideration the entire periphery of the blank with a bifacial (n = 6) or unifacial (n = 3) modality. This shaping echoes the reduction strategy described by Chazan for Wonderwerk Cave (South Africa) when writing “*[…]conceptually*, *the tools are treated as an integrated entity that is first shaped and then retouched*” [248] or by ourselves regarding Namib IV [249]. The shaping always follows the bifacial intersection plane and is followed by two to three final retouch sequences for regularization of the edge delineation. Artifacts vary in raw material, length and width (from 6 to 18 cm and between 4,7 to 9 cm, respectively), and they are mainly plano-convex tools. Or, they have convergent distal edges suggesting important morphometric variability.
- Strategy 2 (n = 8): it consists of “modular shaping” [249]. This strategy is applied to different types of raw material, and initial blanks and metrics are variable. Each shaping sequence shows specifically the location on the blanks and aims to create or modify a specific volumetric pattern (e.g. a distal point, a lateral surface, a rectangular proximal part etc.). The sequences are distinguished by removal chronology and poor overlapping of the sequences (Fig 11). Flaking angles can influence the volumetric pattern of the final tool.
- Strategy 3 (n = 6): all artifacts are in quartz obtained from both cobbles (n = 3) and cortical flakes (n = 3). They present a marginal partial bifacial shaping sequence which appears short and discontinuous. These are only mid-sized tools (5 to 10 cm in length) and all present convergent distal edges. Overall, the final products of this strategy are largely homogenous. The strategy aims to only regularize the initial blanks while retaining some pre-existing volumetric patterns on the blanks, for a specific final objective (Fig 11A)–namely, mid-sized pointed and thin quartz tools.

Overall, these shaped tools (n = 213) present diverse raw materials comprising siliceous rocks (n = 8), quartz (n = 10), quartzite (n = 4) and volcanic rock (n = 1)–even though quartz is particularly associated with strategy number three. Various blank types can be observed: cortical flakes (n = 7), split pebble/cobbles (n = 2), indeterminate flakes (n = 2) or entire cobbles (n = 7). These are also frequently retouched on up to three zones on the periphery. Raw material diversity is absent from the flake assemblage though they would have been easily identified, due to the predominance of quartz and refitting, and it may indicate the possible production of these tools outside of Dungo IV coupled with on-site discard behaviors.

### Multifacial tools

**Fig 12:**
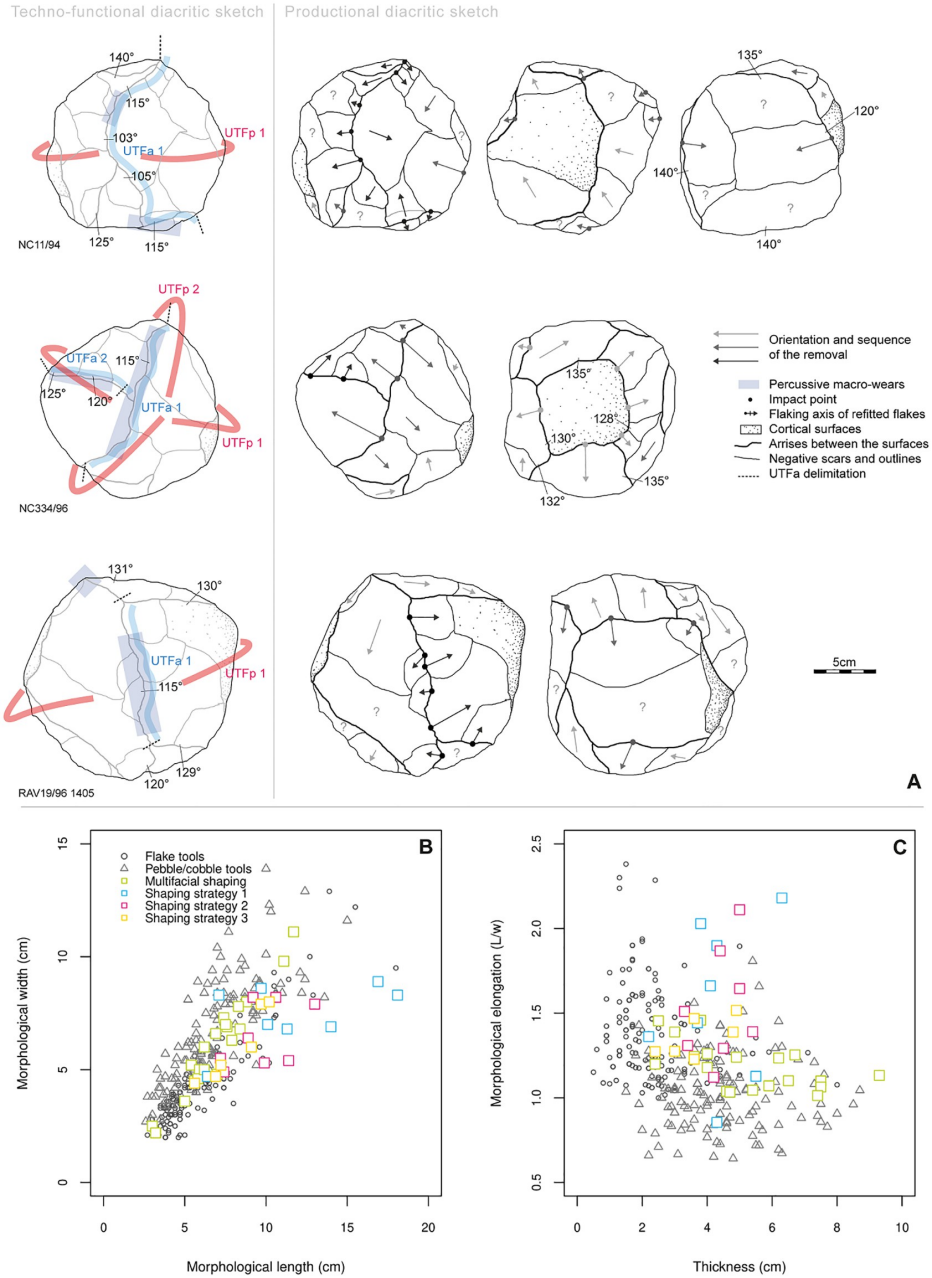
A: examples of polyhedra from Dungo IV showing the consistency between the production and techno-functional approaches. B and C are biplots of morphological length, width, thickness and elongation (Length/width) of the different tool techno-types.

Multifacial shaped tools have been separated from bifacial or unifacial shaped tools due to their specific volumetric configuration resulting from multifacial shaping (Fig 12A). Numerically, these artifacts represent an important part of shaped tools (n = 21) suggesting that multifacial shaping is as important as bifacial or unifacial shaping strategies for the knappers of Dungo IV. This volumetric configuration tends to be on mid-sized cubic to spheroid artifacts (Table 2) and is obtained through shaping using two generations of removals on an entire cobble or pebble. Typologically, it can be referred to as the “polyhedral, sub-spheroid and spheroid” tool types [193, 250]. First generation shaping tends to be multidirectional or orthogonal and provides the overall tool configuration thanks to a progressive opening of angles, whereas the second and final/ultimate generation creates a specific hemispheric crest by alternate removals (Fig 12A). This crest is always opposite to the remaining cortical surface. It presents angle values ranging from 90 to 120°, which are lower than the angles between other sub-surfaces that we could identify, and which range from 120 to 140°. Consequently, we interpret this crest as a potential UTFa—also based on previous studies [192]. On the contrary, the opposite remaining cortical surface is highly likely to have acted as a prehensile part for a powerful grip. Eleven of these tools present macro-use wear patterns described in the following sub-section. The recurrence of this multifacial shaping strategy, associated with redundant techno-functional features, suggests a consistent and well-established mental template referring to a specific macro-tool type.

### Percussive and crushing tools

**Fig 13:**
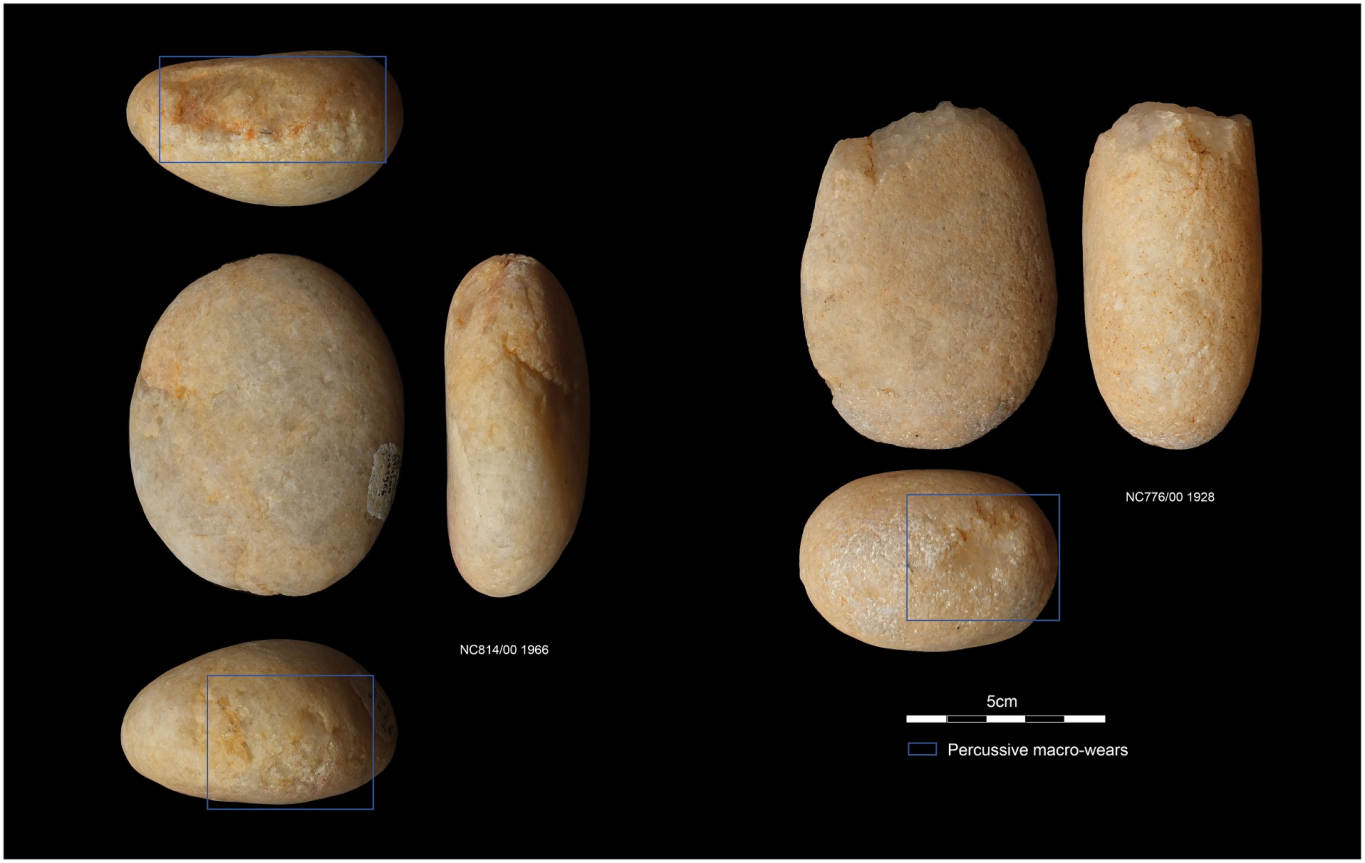
Examples of percussive macro-wear on quartz pebbles from Dungo IV.

Percussive wear patterns are present on 13.3% of the tools, which suggests that on-site percussive activities took place at Dungo IV. Percussive and crushing wear was identified on four types of blank: unmodified pebbles and cobbles (n = 17) (Fig 13), on the cortical convex parts of modified pebbles and cobbles (cobble/pebble tools) (n = 17), and on crests of polyhedra (n = 11), or on the latero-proximal part of bifacial shaped tools (n = 2). In total, 33 artifacts present macro-wear among which, 19 present a parallelepipedal to spherical morphology. We observed macro-wear primarily on crests or on cortical prominences (n = 14), and on plane to slightly convex surfaces (n = 7). The unmodified tool category comprises a set of cobbles and pebbles that retain macro-wear related to percussive or crushing activities. Regarding raw materials, magmatic rocks predominate and it has been argued that they have been specifically targeted for percussive activities while also representing some of the biggest tools for the entire assemblage [169].

### Dungo IV and the southern African coastal plain: A morphometric comparison of shaped tools

**Fig 14:**
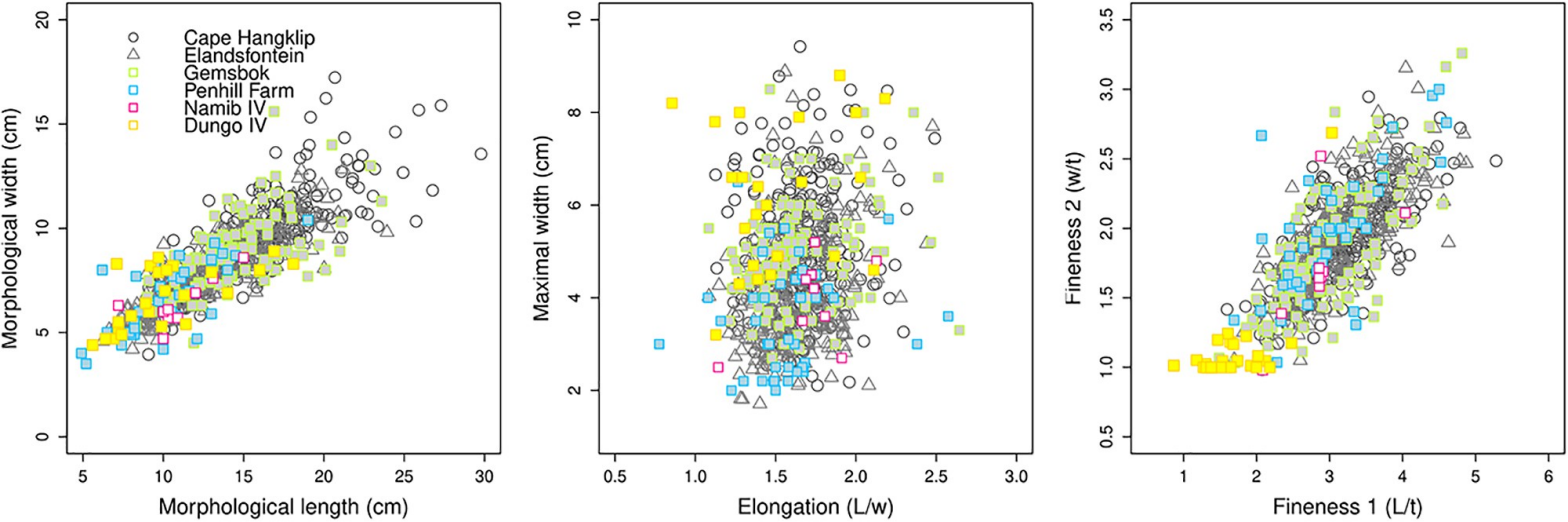
Comparative biplots of morphometric patterns for the shaped tools from Cape Hangklip, Elandsfontein, Gemsbok, Penhill Farm, Namib IV and Dungo IV. Data are available in S3 Table.

Studies comparing ESA shaped tools have shown that morphology is relevant when assessing regional inter-site differences [220, 251–255]. Consequently, here we provide supplementary data (see S2 Table) regarding bifacial and unifacial shaped tools from the previously mentioned five coastal plain sites of southern Africa, which chronologically span the Mid-Pleistocene Transition (Fig 1 and Table 3): Cape Hangklip, Elandsfontein, Gemsbok, Namib IV and Penhill Farm. Metrics and indices are based on the methodology of [169, 220] and measurements were conducted by the authors, except for Cape Hangklip and Elandsfontein that were obtained from the online database of [219]. Comparative results are presented in bivariate plots (Fig 14). Tukey HSD tests were applied to check the significance of p-values (<0.05) between the assemblages for each quantitative variable: length, width, thickness, elongation, fineness indice 1 (Length/maximal thickness) and fineness indice 2 (maximal width/maximal thickness). From this, an important distinction is visible for the Dungo IV LCTs, by fineness indices 1 and 2. This distinction suggests that robust unifacial and bifacial shaped tools occur more frequently at Dungo IV when compared with the other southern African assemblages from the coastal plain. This is confirmed by significant p-values (See S3 Table) and it agrees with the technological observations described above. Overall lengths and widths are highly variable and mimic those trends observed at Penhill Farm and Namib IV. The diversity of reduction stages at Elandsfontein has been argued to be a major factor accounting for the morphometric variability of this assemblage [251]. At Dungo IV, several stages of reduction have been observed, yet the assemblage presents homogeneous fineness indices that suggest there was a well-established mental template.

## Discussion

In this paper, we propose that Dungo IV is a raw material procurement and knapping area, but also an area of multiple activities in a coastal zone. This is supported by numerous refits, the discard of used stone tools (with percussive wear patterns), the core and flake techno-typological characteristics and consistencies, and also, on-site raw material procurement with very few imports.

Regarding the core assemblage, most could be grouped into four categories and we can see specificities among each category, suggesting well-defined and separate “*chaînes opératoires*” for flaking strategies (e.g., different blank types, different core management strategies and varying objectives). Very few cores presented the stigmata that would indicate the application of several different strategies on the same blank. It is interesting to note the absence of SSDA flaking (alternant bifacial or orthogonal) and the scarcity of multidirectional cores, whereas the latter are well-represented among southern African ESA coastal sites such as at Gemsbok [60], Cape Hangklip [122], Penhill Farm [256] and Namib IV [249]. Despite the short reduction sequences which predominate at Dungo IV, we can argue that the flaking systems are not flexible and they are aligned to several specific mental templates. The primary trend is to produce small to medium flakes (<10 cm), whereas evidence for large flake production (>10 cm) has only been observed on two cores and two flakes, suggesting that such blank production was limited at Dungo IV. In this regard, we suggest these large flake blanks form only a very marginal part of the production system for the Dungo IV groups. In contrast, the importance of small to medium size *débordant* flakes, coupled with short flake production, seems to echo the retouched flake assemblage. The organization of a lateral surface opposite to a cutting edge appears to be well-suited for tool configuration, and they were likely anticipated during flake production by obtaining cortical lateral surfaces (short flakes and *débordant* flakes). In addition, the importance of cortical convexities is also a predominant feature in flake production and these products were used for retouch.

Regarding cobble/pebble tools, even though this component primarily exhibits a generalist production strategy (various shapes, sizes and types, and locations of cutting edges), several specific pebble tool implements described here indicate the presence of more specialized operational schemes. These concern macro-tools with a trihedral extremity, surface platform pebble tools, and micro-robust pebble tools (<5 cm in length). Among the shaped tools, we could identify highly variable bifacial and unifacial shaping strategies, but consistent morphometrics that indicate a trend for producing middle sized artifacts (5–10 cm). The multifacial shaping of macro-tools, that were possibly used for percussive or pounding activities, shows a different pattern where there is more standardized production giving rise to morphometric and techno-functional homogeneity. This suggests a more specialized operational scheme for producing spherical to cubic tools.

When comparing Dungo IV with the southern African coastal plain MPT ESA sites (see detailed bibliography in Table 3), we observed specific patterns in fineness indices, in addition to a higher frequency of more robust shaped tools at Dungo IV. All these sites indicate the use of pebble/cobble blanks for flaking and shaping, but also, for use in pebble tool configuration, while also exhibiting important components of bifacially shaped tools on large flakes. Typologically, large flakes, handaxes and cleavers (forming classic ESA Large Cutting Tools) have been reported among all these sites except at Dungo IV, where cleavers are absent and large flake (>10cm) production is sporadic. Despite the appearance of the pebble/cobble tools, the Dungo IV assemblage presents well-defined operational schemes characterized by specific yet diverse roles of pebble/cobble cortical convexities. We observed the role of the cortical convexities in:

- tools with a structure which relies on these natural convexities,
- recurrent and well-differentiated flaking strategies which exploit natural convexities in different ways,
- the retouch of cortical flake blanks,
- the shaping and retouch and pebbles and cobbles.

Variability is more evident in bifacial/unifacial shaping, cobble/pebble tools reduction and retouch types. In addition, we argue that several macro-tool categories are associated with specific production schemes which take advantage of pebble/cobble convexities: picks on cobbles, polyhedra, and thick, small plano-convex cortical flake tools. This can be related to the supply of beach quartz pebbles/cobbles in a landscape where other fine-grained raw materials are available, but are more rarely selected. Given the location of Dungo IV, on a coastal pebble/cobble outcrop, we propose that local populations specifically targeted these raw materials [169] as they were suitable for the toolkit they aimed to produce. Consequently, the lithic analysis results argue for an adaptation of several reduction strategies and tool types tied to the volumetric properties of beach pebbles and cobbles, and particularly for quartz.

Based on stratigraphic correlations within the Middle Pleistocene Red Sand unit, Dungo IV is part of a broader complex of contemporary or sub-contemporary sites primarily located within several non-perennial river basins of the coastal plain [79, 132, 157, 166, 168, 169, 174, 175, 177]. These sites—among which include the localities of Mormolo, Uiche, Benguela, Macaca, Ponta das Vacas or Sombreiro—are all characterized by the use of beach quartz pebbles and cobbles during lithic production [157, 158]. Artifact production also includes a range of additional raw materials, but quartz is the most abundant on the +100 m raised beaches. This suggests the strategic and location-specific exploitation of coastal outcrops during the ESA [169]. Other raw materials form only a minor component (e.g. magmatic rocks, sandstone, quartzite and more rarely, siliceous rocks), and they likely originate from the End Rift Unconformity [170] located ca. 20 km inland from the coastal sites. A recent study [169] presents a detailed understanding of raw material availability in the landscape surrounding Dungo IV, characterizing the pebble and cobble deposits that would have been available at the time of hominin occupation. This study argues for an absence of selection based on pebble/cobble shape preferences, except for unmodified percussive tools, and it also confirmed that pebbles/cobbles were being selected in a way that mimicked the coastal lithological resources. This means that the morphometric and lithological trends evident in the Dungo IV assemblage are similar to those trends observed today in pebble/cobble outcrops of the Dungo raised beach, and the neighboring raised beach of Mormolo. At Dungo V, a similar industry has been described and it is associated with whale remains [34, 178, 179], suggesting that coastal resource use beyond mere lithological sources is possible, and subsistence strategies at the very least may have been influenced by the available resources.

From a broader perspective, an important contrast is observable between the coastal ESA sites reported along the Angolan coast—especially south of Luanda, all along the Benguela Province coast and within the Namib Province—characterized by quartz pebble industries among Middle Pleistocene raised beaches [79, 132, 157, 257], and inland Angolan ESA sites (e.g. Angolan Plateau). These latter sites are represented by a hotspot of undated ESA localities in the extreme north-east of the country, between the Congo River and its Kasai tributary in the Lunda-Norte Province, and the site of Capangombe in the Huila Province—much closer to Benguela. Collectively, this Lunda-Norte ESA corpus—though requiring important geoarchaeological and techno-typological reappraisal—is primarily characterized by shaping strategies and/or massive artifacts related to Congo Basin techno-typological trends during Pleistocene times [132, 258–260]. At Capangombe, two undated archaeological layers preserve thousands of artifacts attributed to the Final Acheulean or ESA/MSA transition [261, 262]. Capangombe presents a wide range of Levallois products, cleavers and various types of handaxes on siliceous rock types [261, 262] suggesting a very different technology from the pebble/cobble based ESA industries along the Angolan coast—which may also be older. Consequently, we argue that the Benguela corpus, including Dungo IV and the surrounding sites, provides a specific concentration of MPT ESA coastal occupations so far with no equivalent inland. This supports the geographic concentration of ESA hominin groups in the coastal plain, where there are small running rivers only. This contrasts with the situation in South Africa or in Namibia where ESA sites are continuously encountered on the coast and inland [60, 78, 121, 263, 264]. It is also interesting to mention the Orange-Senqu River basin [265, 266] in South Africa marking an important difference in hydrographical settings between the Angolan coast and the coast of South Africa and southern Namibia. When considering the richness of ESA sites in this basin, it likely played an important role in the dispersal of early hominins in southern Africa and its relation to coastal dispersals must be re-investigated.

In this paper we list specific technological patterns evident at Dungo IV: a trend for robust micro- to large macro-tools, several production strategies taking advantage of natural convexities of pebbles and cobbles in various ways, an important component of percussive tools, a specific focus on coastal outcrops, and a quartz-based industry. Altogether, these patterns highlight a local technological trajectory very different from the Acheulean of the Middle Pleistocene of southern Africa, usually rich in LCTs even when exploiting pebble/cobble outcrops [60, 114, 138, 240, 248, 251, 254, 267–269]. No similarities occur when looking at the Middle Pleistocene assemblages of Central Africa, characterized by massive shaped tools, such as at Elarmékora in Gabon [80] or in the Lunda-Norte region of Angola [132, 258]. Consequently, we propose the existence of a “local technological trajectory” at Dungo IV, whereby ESA people adapted their technology within a delimited area and during a specific period. Indeed, they developed an atypical lithic industry and took advantage of pre-existing lithological and morphometric characteristics in the local coastal marine pebble/cobble outcrops. This local technological trajectory is characterized by strategies that take advantage of natural convexities of beach pebbles and cobbles, for producing robust tools of all size categories with potentially diverse active parts (percussive surfaces, cutting edges, pointed and piercing tips). In addition, artifacts barely reach 10 cm long but they are notably thick, and the natural pebble/cobble convexities are systematically advantageous for the knappers. Robustness is present both in the potentially active and handled parts, suggesting a toolkit resistant to forceful activities (across both prehensile and active tool portions). LCTs are scarce and appear to have played a minor role in the technical system (production and use) described at both Dungo IV and Dungo V [178, 179]. Historically, LCTs have been found in surface contexts in the Dungo River Valley [79, 132, 177], but the *in situ* Dungo IV assemblage does not contain any. These LCTs may relate to other phases or types of occupation in the valley that are yet to be discovered. At Dungo IV, a major characteristic are the pebble/cobble tools, obtained through generalist and specialized operational schemes, which exhibit notable diversity both in terms of their overall morpho-structure [169] and in their potentially active parts.

These specific technical patterns are restricted spatially, occurring only in the coastal ESA sites and within the +100 m raised beaches of the Benguela Province. Though other coastal ESA occurrences have been reported within Middle Pleistocene raised beaches in Angola, this site accumulation has no echo inland. ESA inland sites present very different technological patterns. We argue for a specific coastal settlement in Dungo IV and its surroundings, where coastal lithological resources played an important role in the technical system of populations and are highly likely to have influenced both the nature of the toolkits and the production schemes and mental templates of the knappers.

This paper clearly documents the use of coastal environments and resources by hominins since at least the beginning of Middle Pleistocene in southern Africa thanks to the repeated and extended occupation of the coast for several activities, among which includes raw material procurement in marine pebble/cobble deposits. These occupations also provide proof for specific technical behaviors highly influenced and adapted to coastal lithological resources. We suggest the integration of coastal landscapes within ESA hominin territories, and we suggest that the path toward coastal adaptation is a process much more deeply rooted in time—concurring with the hypothesis proposed by Fischer et al. [57].

This paper shows that lithic resources may have played a role in coastal ‘*territorialization*’–the process during which spaces get integrated into group territories [147]. In this case, the record so far suggests that systematic coastal raw material procurement predates systematic alimentary coastal foraging, and raw materials may have acted as an attractive resource in southern Africa impacting hominin dispersals along the coast. It also confirms the hypothesis proposed in South Africa for ESA surface finds in the Namaqualand [5] and in the KwaZulu-Natal Province [85]. Both authors suggest Acheulean groups were moving to the coast to procure raw materials and this would explain why typical ESA artifacts are scattered among the Middle Pleistocene raised beaches. In southern Namibia, Corvinus [60] proposed continuous ESA occupations across 100 km of early Middle Pleistocene raised beaches, from Gemsbok to Uubvley, and correlated them to ESA sites at least 40 km inland along the Orange River. Though more data are needed (i.e., excavations and absolute dating) it is a hypothesis which echoes our observations in the Benguela Province where the ESA is also continuous along the coast. However, the primary difference is that the presence of the Orange River hydrographic network may have emphasized the connection between coastal and inland sites in southern Namibia. In the case of Dungo IV, investigating the lithic assemblage in greater detail will allow us to raise new questions about the chronological gap that exists in southern Africa between early Middle Pleistocene populations and, predictable resource foraging at the very end of the Middle Pleistocene that is associated with MSA behavioral changes [20, 21]. The latter is usually related to the development and emergence of *Homo sapiens* during the second half of the Middle Pleistocene [270, 271]. Dungo IV provides evidence that coastal resources influenced hominin behaviors since at least the beginning of the Middle Pleistocene in southern Africa, and this contributes to the trend in pushing back such behaviors and disassociating them with the younger MSA. Therefore, it challenges the impression of an abrupt emergence of behaviors, as argued for the MSA—visibility that is otherwise linked to differential preservation biases and post-depositional perturbation of sites that likely reflect different landscape patterns.

Earlier evidence of coastal territorialization could also be proposed considering the corpus of coastal Early and Middle Pleistocene Acheulean sites in Northern Africa, and particularly in Morocco [272–275]. It has even been suggested that Northern African Acheulean sites share technological affinities with sites in the Iberic Peninsula, potentially highlighting the important role of coastal paths for early hominin dispersals [276]. In addition, it has been shown that several non-human primate species are consuming fish and aquatic resources [277] among which two examples show a diet adapted to coastal shellfish resources [278, 279]. Indeed, specialized coastal foraging strategies cannot be unique only to modern humans and modern behaviors. In fact, the southern African record shows a gradual intensification of coastal activities by humans with: hominin dispersals in the coastal plain during the Early Pleistocene [59], an integration of coastal resources in ESA hominin territories at least by 600 ka (such as in the Benguela Province), the occasional consumption of marine resources from the end of the Middle Pleistocene—or even before by scavenging stranded carcasses [178]–and, an intensification of coastal resource foraging from MIS 5 [17] and particularly at the end of the Pleistocene [19]. Concurrently, similar trends occurred in other parts of the world, such as the development of marine resource foraging by Neanderthal groups from MIS5 [280–282], Early Pleistocene coastal dispersals in North Africa [273] and early Middle Pleistocene coastal raw material exploitation in the Early Paleolithic of the Britany coast in France [283–285]. Consequently we concur with our colleagues [26], who argue for a revisiting of the hypothesis claiming the southern African coastal environment acted as a pulse for the emergence and development of modern behaviors and *Homo sapiens*. The revision of this hypothesis must take into consideration both the inland contemporary record and the coastal Early and Middle Pleistocene record.

## Conclusion

Initially, the appearance of a “pebble industry” raised questions about the chrono-cultural attribution of the lithic assemblage at Dungo IV [153]. A question that was reinvigorated when new dating of the coastal site pointed toward an early Middle Pleistocene burial duration, between 614.5 ± 9.5 ka and 662.05 ± 10.24 ka [156] that suggested contemporaneity with the later Acheulean in southern Africa. Our detailed lithic analysis exhibits several patterns which allow us to associate Dungo IV to the Acheulean techno-complex, such as prepared cores and bifacial symmetry in shaping strategies. However, this evidence is scarce, marginal and poorly represented in the assemblage. We highlight different and well-defined flaking and shaping strategies reflecting precise mental templates despite short “*chaînes opératoires*”. In addition, the coastal lithological environment seems to have played a pivotal role in the technical system adopted by local populations (production strategies and toolkits). The site preserves a quartz-based industry where all sizes of rounded gravels were exploited, and where knappers systematically took advantage of the natural convexities on these coastal clasts in many different ways. Though the site falls into the later Acheulean chronological range [90, 114, 124, 240, 268, 286], the technological patterns of the Acheulean are rare and Large Cutting Tool production is very marginal, suggesting little attention was given to long and thin cutting edges amongst the larger toolkit. Instead, robust cutting edges and morpho-structures, micro- to macro-tool sizes, and shaped to simply retouched blanks, are well-represented, along with percussive wear. Our results support various on-site activities among which include knapping activities, particularly evident through refits and local raw material procurement [169]. All these patterns show how specific the ESA technology is at Dungo IV, and how coastal lithological resources have influenced operational schemes giving rise to a local technological trajectory.

From a geographic and chronological perspective, Dungo IV is associated with a wider complex of early Middle Pleistocene sites contained within Red Sand deposits overlying a Calabrian marine conglomerate on the +100 m raised beaches of the Benguela Province [160]. This marine conglomerate points toward a coastal plain opening at the very end of the Early Pleistocene in this area, as has been suggested for South Africa by Compton [100]. All the sites reported among the +100 m Middle Pleistocene raised beaches exhibit this quartz gravel use [157, 158]. These sites have no equivalent in terms of technology or chronology inland, suggesting a unique complex of ESA coastal sites. In addition, this points toward an extended and repeated use of coastal lithological resources for stone tool production, confirming the coastal fringe was well-integrated in early hominins’ landscape and mobility strategies, at least for raw material procurement. However, with the exception of Dungo V [34, 178], subsistence strategies remain unknown so it is not possible to evaluate to which extent coastal resources may have also influenced the diet of these hominins. Consequently, regarding the available data, we argue that raw material procurement likely served to help attract populations, leading to the progressive occupation of coastal areas. Subsequently, raw material procurement appears as a first step toward coastal resource acquisition and management, and it predates the more extensive and well-preserved coastal adaptations that occur from the very end of the Middle Pleistocene through to the Late Pleistocene [17].

In the broader context of the coastal southern African Stone Age, several sites dated to the Mid-Pleistocene Transition and located within the southern African coastal plain support an extended presence of hominins in coastal landscapes even though artifacts are often scattered and site formation processes are very challenging [57, 59]. This shows that Dungo IV may not be the only evidence of coastal “*territorialization*” by hominins during Acheulean times in southern Africa—it potentially occurs in other coastal areas of Africa [89, 273, 275, 287, 288].

Finally, all these southern African ESA coastal sites are still too rarely mentioned despite their high potential to provide details on: early hominin coastal dispersals (a crucial step prior to “adaptation”), paleo-demographic dynamics along watercourses, early technical behaviors in coastal contexts, and, the origins of adaptation to coastal ecological niches much earlier than what is currently preserved in the Middle Stone Age record of southern Africa.

## Supporting information

S1 Table Coastal ESA sites and localities from the coastal plain. Geographic and contextual information of ESA sites and localities reported in the southern African coastal plain since the early 20th century based on the synthesis of bibliographic data.
(DOCX)

S2 Table Morphometric data set used for intersite comparison. Length, maximal width, maximal thickness, elongation and fineness of the shaped tools from Gemsbok, Penhill Farm, Dungo IV, Namib IV, Elandsfontein and Cape Hangklip.
(DOCX)

S3 Table Tukey HSD Test results for the intersite comparison. Results are presented for each qualitative variable and for each site.
(DOCX)
