# Supplementary material for: Evidence for Earlier Stone Age ‘coastal use’: The site of Dungo IV, Benguela Province, Angola
Source: PLoS One. 2023 Feb 24;18(2):e0278775. doi: 10.1371/journal.pone.0278775 (PMC9955982; doi:10.1371/journal.pone.0278775)
Supplement: S1 Table — Geographic and contextual information of ESA sites and localities reported in the southern African coastal plain since the early 20th century based on the synthesis of bibliographic data. (DOCX) [file pone.0278775.s001.docx]

**Supplementary Information – File 1**

List of the Earlier Stone Age localities used to map the Figure 1. Latitude, Longitude, Country, Context and Age are provided when available and Reference used for each locality. Please contact authors to obtain a .csv version of the database.

The latitude and longitude have been founded using Google Earth and published data or published map and Degrees Minutes Seconds converted Decimal Degrees using the following site: <https://www.coordonnees-gps.fr/>. The variability in precision is due to the original source used and some locality names may have changed since the original publication. In the table we use the following abbreviation: SA: South Africa, ANG: Angola, MOZ: Mozambique, NAM: Namibia, SURV: survey, UNK: unknown, EXC: excavations, MIN: mining context, MPT: Mid-Pleistocene Transition (1.2-0.5 Ma), MP: Middle Pleistocene ESTIM: estimation, MNAB: Museu Nacional de Arqueologia de Benguela, NMN: National Museum of Namibia.

| Site | Latitude | Longitude | Country | Context | Age | Dating | Reference |
| --- | --- | --- | --- | --- | --- | --- | --- |
| Addo Drift | -33.554865 | 25.681956 | SA | SURV |  | NO | Breuil, 1945; Davies, 1971 |
| Alexander Bay | -28.61805 | 16.484975 | SA | UNK |  | NO | Davies, 1973; Davies & Walsh, 1955 |
| Anyskop | -32.972214 | 18.116666 | SA | UNK |  | NO | Conard, 2003; Kandel et al., 2006 |
| Atmar Farm | -33.466545 | 25.527793 | SA | EXC | 0.65 ± 0.12 Ma | YES | Granger et al., 2013; Lotter, 2016 |
| B4NW-1 | -31.3158 | 29.972 | SA | UNK | c. 300 ka | YES | Fisher et al., 2013 |
| Bats Cave | -33 | 27.9166667 | SA | UNK |  | NO | Keller, Rudner, Deacon, Davies Clark, 1967 |
| Bellville Brickfield | -33.9 | 18.6333333 | SA | UNK |  | NO | Keller, Rudner, Deacon, Davies, Clark, 1967 |
| Bernol Farm | -33.473121 | 25.605305 | SA | EXC | 1.14 ± 0.20 Ma | YES | Lotter & Kuman, 2018a |
| Bietou-Keurbooms Estuary (several localities) | -34.02 | 23.39 | SA | SURV |  | NO | Davies, 1972 |
| Blaaukrantz (Humansdorp) | -34 | 24.75 | SA | UNK |  | NO | Keller, Rudner, Deacon, Davies, Clark, 1967 |
| Bonza Bay | -32.974613 | 27.965012 | SA | UNK |  | NO | Cooke, 1941 |
| Brakfontein - Riversdale | -34.035418 | 21.244303 | SA | SURV |  | NO | Davies, 1971 |
| Cape Flats | -34.033333 | 18.5666667 | SA | UNK |  | NO | Keller, Rudner, Deacon, Davies, Clark, 1967 |
| Cape Hangklip | -34.39 | 18.83 | SA | SURV | 700-400 ka | ESTIM | Davies, 1972; McNabb et al., 2004 |
| Cape Maclear (C103) | -34.355 | 18.495303 | SA | SURV |  | NO | Davies, 1972 |
| Cape Seal | -34.10705 | 23.412093 | SA | UNK |  | NO | Keller, Rudner, Deacon, Davies, Clark, 1967 |
| Cape St, Francis | -34.20523 | 24.828525 | SA | UNK |  | NO | Keller, Rudner, Deacon, Davies, Clark, 1967 |
| Clansthal | -30.083333 | 30.9166667 | SA | UNK |  | NO | Keller, Rudner, Deacon, Davies, Clark, 1967 |
| Cliffdale | -29.916667 | 30.6666667 | SA | UNK |  | NO | Keller, Rudner, Deacon, Davies, Clark, 1967 |
| Clifton | -33.945158 | 18.378168 | SA | UNK |  | NO | Keller, Rudner, Deacon, Davies, Clark, 1967 |
| Coega Valley | -33.75 | 25.75 | SA | SURV |  | NO | Davies, 1972 |
| Constantia | -34.033333 | 18.4333333 | SA | UNK |  | NO | Keller, Rudner, Deacon, Davies, Clark, 1967 |
| De Dam | -30.833333 | 17.8666667 | SA | UNK |  | NO | Keller, Rudner, Deacon, Davies, Clark, 1967 |
| Deal Party | -33.880556 | 25.62 | SA | SURV |  | NO | Davies, 1972 |
| Doornrivier, Olifants River | -34.1 | 18.35 | SA | UNK |  | NO | Keller, Rudner, Deacon, Davies, Clark, 1967 |
| Duineplaas - Gouritz Mouth | -34.343424 | 21.882714 | SA | SURV |  | NO | Davies 1972 |
| Duinefontein | -33.657176 | 18.421577 | SA | EXC | c. 200 ka | ESTIM | Feathers, 2002 |
| Duiwenhoks | -34.356896 | 21.011951 | SA | SURV |  | NO | Davies, 1971 |
| East London | -32.993456 | 27.864454 | SA | MIN |  | NO | Macfarlane, 1935 |
| Elandsfontein | -33.108333 | 18.2416 | SA | EXC | 1-0.6 Ma | YES | Braun et al., 2013; Singer & Wymer, 1968 |
| Fish Hoek Brickfield | -34.15 | 18.4166667 | SA | UNK |  | NO | Keller, Rudner, Deacon, Davies, Clark, 1967 |
| Formoan | -34.033333 | 23.1833333 | SA | UNK |  | NO | Keller, Rudner, Deacon, Davies, Clark, 1967 |
| Garden Route Casino Road | -34.191667 | 22.0916667 | SA | construction | | NO | Thompson, 2009 |
| Geelhoutboom | -34.072555 | 24.725511 | SA | UNK |  | NO | Laidler, 1947 |
| Geelhoutboom, Humansdorp | -34 | 24.6833333 | SA | UNK |  | NO | Keller, Rudner, Deacon, Davies, Clark, 1967 |
| George | -33.933333 | 22.45 | SA | UNK |  | NO | Keller, Rudner, Deacon, Davies, Clark, 1967 |
| Great Fish River | -33.489802 | 27.114338 | SA | SURV |  | NO | Davies, 1971 |
| Groenvley, Knysna | -34.016667 | 23.05 | SA | UNK |  | NO | Keller, Rudner, Deacon, Davies, Clark, 1967 |
| Grootrivier Pass, Knysna | -34 | 23 | SA | UNK |  | NO | Keller, Rudner, Deacon, Davies, Clark, 1967 |
| Hawston | -34.383333 | 19.1333333 | SA | UNK |  | NO | Keller, Rudner, Deacon, Davies, Clark, 1967 |
| Heidelberg, Cape | -34.15 | 20.9166667 | SA | UNK |  | NO | Keller, Rudner, Deacon, Davies, Clark, 1967 |
| Hen and Chickens estate, Wynberg | -34.016667 | 18.4666667 | SA | UNK |  | NO | Keller, Rudner, Deacon, Davies, Clark, 1967 |
| Hermanus | -34.416667 | 19.2333333 | SA | UNK |  | NO | Keller, Rudner, Deacon, Davies, Clark, 1967 |
| Hibbendene | -30.740558 | 30.4582863 | SA | UNK |  | NO | Keller, Rudner, Deacon, Davies, Clark, 1967 |
| Hlimbitwa Mouth | -33.083333 | 18.3333333 | SA | UNK |  | NO | Keller, Rudner, Deacon, Davies, Clark, 1967 |
| Hluwehluwe | -28.499246 | 32.4120104 | SA | UNK |  | NO | Keller, Rudner, Deacon, Davies, Clark,1967 |
| Hopefield | -33.083333 | 18.3333333 | SA | UNK |  | NO | Keller, Rudner, Deacon, Davies, Clark, 1967 |
| Humansdorp | -34.039545 | 24.578626 | SA | SURV |  | NO | Davies, 1971 |
| Ifafa "rive nord" | -30.666667 | 30.6666667 | SA | UNK |  | NO | Keller, Rudner, Deacon, Davies, Clark, 1967 |
| Ifafa "rive sud" | -30.583333 | 30.8333333 | SA | UNK |  | NO | Keller, Rudner, Deacon, Davies, Clark, 1967 |
| Ifafa Beach II | -30.462934 | 30.648162 | SA | UNK |  | NO | Keller, Rudner, Deacon, Davies, Clark, 1967 |
| Illovo Lagoon | -30.166667 | 30.8333333 | SA | UNK |  | NO | Keller, Rudner, Deacon, Davies, Clark, 1967 |
| Impapasi Hill | -28.5 | 32.4166667 | SA | UNK |  | NO | Keller, Rudner, Deacon, Davies, Clark, 1967 |
| Instant River | -28.5 | 32.4166667 | SA | UNK |  | NO | Keller, Rudner, Deacon, Davies, Clark, 1967 |
| Instant River | -28.5 | 32.4166667 | SA | UNK |  | NO | Keller, Rudner, Deacon, Davies, Clark, 1967 |
| Isipingo Beach Road | -30.083333 | 30.8333333 | SA | UNK |  | NO | Keller, Rudner, Deacon, Davies, Clark, 1967 |
| Jakkalskop | -34.067991 | 21.296993 | SA | SURV |  | NO | Davies, 1971 |
| Kariega Valley | -33.666667 | 26.65 | SA | UNK |  | NO | Keller, Rudner, Deacon, Davies, Clark, 1967 |
| Kaross | -28.482985 | 16.649238 | SA | SURV |  | NO | Davies, 1973 |
| Kasoaga River Mouth | -33.666667 | 26.7166667 | SA | UNK |  | NO | Keller, Rudner, Deacon, Davies, Clark, 1967 |
| Keurboom River Mouth | -34.016667 | 23.3833333 | SA | UNK |  | NO | Keller, Rudner, Deacon, Davies, Clark, 1967 |
| Keurbooms Bridge | -34.006885 | 23.39416 | SA | SURV |  | NO | Davies, 1971 |
| Keurbooms Gorges | -33.942438 | 23.596104 | SA | SURV |  | NO | Davies, 1971 |
| Klasies River | -34.104507 | 24.391802 | SA | UNK | MP | ESTIM | Laidler, 1947 |
| Klawer | -31.766667 | 18.5833333 | SA | UNK |  | NO | Keller, Rudner, Deacon, Davies, Clark, 1967 |
| Klawervlei, Darling | -33.4 | 18.3333333 | SA | UNK |  | NO | Keller, Rudner, Deacon, Davies, Clark, 1967 |
| Kleinemonde | -33.53 | 27.05 | SA | SURV |  | NO | Davies, 1973 |
| Kleinzee | -29.672419 | 17.0500805 | SA | SURV |  | NO | Davies, 1973; Davies & Walsh, 1955 |
| Kleinzee Gorge | -29.644208 | 17.096825 | SA | SURV |  | NO | Davies, 1973 |
| Klip-Kop | -34.4 | 19.24 | SA | SURV |  | NO | Davies, 1973 |
| Knysna Western Head | -34.080799 | 23.068894 | SA | UNK | MP | ESTIM | Breuil, 1945; Mortelmans, 1945 |
| Koekenaap | -31.527946 | 18.296674 | SA | SURV |  | NO | Davies, 1973; Davies & Walsh, 1955 |
| Kristenbosch | -33.983333 | 18.3833333 | SA | UNK |  | NO | Keller, Rudner, Deacon, Davies, Clark 1967 |
| Krommerrivier | -34.069967 | 24.648882 | SA | SURV |  | NO | Davies, 1971 |
| Kubakaga River | -28.498869 | 32.4120747 | SA | UNK |  | NO | Keller, Rudner, Deacon, Davies, Clark 1967 |
| Langebaanweg | -32.975462 | 18.12777 | SA | UNK |  | NO | Kandel et al., 2006 |
| Langeberg, Durbanville | -33.833333 | 18.6333333 | SA | UNK |  | NO | Keller, Rudner, Deacon, Davies, Clark 1967 |
| Leeuwpont +50m | -29.916667 | 30.7333333 | SA | UNK |  | NO | Keller, Rudner, Deacon, Davies, Clark 1967 |
| Ligtenbos | -34.274266 | 21.595728 | SA | UNK |  | NO | Breuil, 1948 |
| Malmesbury | -33.466667 | 18.6666667 | SA | UNK |  | NO | Keller, Rudner, Deacon, Davies, Clark 1967 |
| Marianhill | -29.866667 | 30.9 | SA | UNK |  | NO | Keller, Rudner, Deacon, Davies, Clark 1967 |
| Maulbach - Hengelaarskroonstrand | -34.066134 | 24.2121314 | SA | SURV |  | NO | Davies, 1971 |
| Mbotyi | -31.454369 | 29.714205 | SA | SURV |  | NO | Davies, 1971 |
| Melkboom 2,5km | -31.604846 | 18.405354 | SA | SURV |  | NO | Davies, 1973 |
| Melkhoutfontein - Gourtz Estuary | -34.374041 | 21.421374 | SA | SURV |  | NO | Davies, 1971 |
| Melkhoutkraal | -34.336011 | 21.401768 | SA | SURV |  | NO | Davies, 1971 |
| Mkuzi River | -27.666667 | 32.1666667 | SA | UNK |  | NO | Keller, Rudner, Deacon, Davies, Clark 1967 |
| Mkuzi River | -27.666667 | 32.1666667 | SA | UNK |  | NO | Keller, Rudner, Deacon, Davies, Clark 1967 |
| Montagu Cave | -33.783333 | 20.1166667 | SA | UNK | MP | YES | Keller, 1973 |
| Monte Venda - Vlermuisklip | -31.566076 | 18.339436 | SA | SURV |  | NO | Davies, 1973 |
| Montrose Avenue - East London | -32.995373 | 27.922495 | SA | SURV |  | NO | Davies, 1971 |
| Mowbray (Medical School) | -33.95 | 18.4666667 | SA | UNK |  | NO | Keller, Rudner, Deacon, Davies, Clark 1967 |
| Moyeni - Old Woman's River | -33.459012 | 27.124246 | SA | SURV |  | NO | Davies, 1971 |
| Mpekweni | -33.432686 | 27.215908 | SA | SURV |  | NO | Davies, 1971 |
| Msikaba (plusieurs localites) | -31.315913 | 29.973422 | SA | SURV |  | NO | Davies, 1971 |
| Mtubatuba Beach II | -28.5 | 32.4166667 | SA | UNK |  | NO | Keller, Rudner, Deacon, Davies, Clark 1967 |
| Mtubatuba Beach II | -28.5 | 32.4166667 | SA | UNK |  | NO | Keller, Rudner, Deacon, Davies, Clark 1967 |
| Mulder's vlei, Faure | -34.033333 | 18.7666667 | SA | UNK |  | NO | Keller, Rudner, Deacon, Davies, Clark 1967 |
| Nahoon River | -32.983333 | 27.95 | SA | UNK |  | NO | Keller, Rudner, Deacon, Davies, Clark 1967 |
| Newlands | -33.983333 | 18.4833333 | SA | UNK |  | NO | Keller, Rudner, Deacon, Davies, Clark 1967 |
| Noordhoek | -34.116667 | 18.4166667 | SA | UNK |  | NO | Keller, Rudner, Deacon, Davies, Clark 1967 |
| Oyster Bay | -34.170477 | 24.647834 | SA | SURV |  | NO | Davies, 1971 |
| Palmiet River Mouth | -34.333333 | 19 | SA | UNK |  | NO | Keller, Rudner, Deacon, Davies, Clark 1967 |
| Penhill Farm | -33.595385 | 25.707599 | SA | EXC | 1.3-1 Ma | YES | Lotter, 2016; Lotter & Kuman, 2018 |
| Pienaarsrivier - Riversdale | -34.035418 | 21.244303 | SA | SURV |  | NO | Davies, 1971 |
| Pinhurst | -34.033333 | 18.4333333 | SA | UNK |  | NO | Keller, Rudner, Deacon, Davies, Clark 1967 |
| Pongela | -27.666667 | 32.3333333 | SA | UNK |  | NO | Keller, Rudner, Deacon, Davies, Clark 1967 |
| Pongola canal terrasse | -28.166667 | 32.3333333 | SA | UNK |  | NO | Keller, Rudner, Deacon, Davies, Clark 1967 |
| Pongola tributary | -28.166667 | 32.3333333 | SA | UNK |  | NO | Keller, Rudner, Deacon, Davies, Clark 1967 |
| Port Beaufort - Bree Estuary | -34.405411 | 20.824731 | SA | UNK |  | NO | Davies, 1973 |
| Port Elizabeth | -31.040833 | 30.228011 | SA | UNK |  | NO | Keller, Rudner, Deacon, Davies, Clark 1967 |
| Port Nolloth | -29.296116 | 16.882213 | SA | SURV |  | NO | Davies, 1973 |
| Port Shepton | -30.723 | 30.444 | SA | UNK | MP | ESTIM | see Hendey & Cook, 1985; Hobday & Orme, 1974 in Fisher et al., 2013 |
| Redburn, Plettenberg Bay | -34.016667 | 23.3333333 | SA | UNK |  | NO | Keller, Rudner, Deacon, Davies, Clark 1967 |
| Redhouse Station | -33.83925 | 25.5694 | SA | SURV |  | NO | Davies, 1972 |
| Riverside, Klein-Brak | -34 | 22.15 | SA | SURV |  | NO | Davies, 1972 |
| Rondebosch | -33.966667 | 18.4666667 | SA | UNK |  | NO | Keller, Rudner, Deacon, Davies, Clark 1967 |
| Sandkop Mining Area | -29.729763 | 17.061556 | SA | SURV |  | NO | Case, 2018 |
| Sandy Bay | -34.2 | 18.3 | SA | UNK |  | NO | Keller, Rudner, Deacon, Davies, Clark 1967 |
| Shisaekop | -30 | 30.8333333 | SA | UNK |  | NO | Keller, Rudner, Deacon, Davies, Clark 1967 |
| Signal Hill | -33.933333 | 18.45 | SA | UNK |  | NO | Keller, Rudner, Deacon, Davies, Clark 1967 |
| Silwereik | -31.529526 | 18.27771 | SA | SURV |  | NO | Davies, 1973 |
| Simonstown Barracks | -34.2 | 18.4333333 | SA | UNK |  | NO | Keller, Rudner, Deacon, Davies, Clark 1967 |
| Site 11 Port Edward Lagoon Head Gravel - Bridge | -31.047 | 30.22 | SA | SURV |  | NO | Davies, 1970 |
| Site 13 | NOT FOUND | NOT FOUND | SA | SURV |  | NO | Davies, 1970 |
| Site 15 | NOT FOUND | NOT FOUND | SA | SURV |  | NO | Davies, 1970 |
| Site 19 Port Edward Beacon Hill | NOT FOUND | NOT FOUND | SA | SURV |  | NO | Davies, 1970 |
| Site 23 | NOT FOUND | NOT FOUND | SA | SURV |  | NO | Davies, 1970 |
| Site 27°06 | NOT FOUND | NOT FOUND | SA | SURV |  | NO | Davies, 1970 |
| Site 28°50' | NOT FOUND | NOT FOUND | SA | SURV |  | NO | Davies, 1970 |
| Site 30°06 15m beach | NOT FOUND | NOT FOUND | SA | SURV |  | NO | Davies, 1970 |
| Site 38 | NOT FOUND | NOT FOUND | SA | SURV |  | NO | Davies, 1970 |
| Site 39 | NOT FOUND | NOT FOUND | SA | SURV |  | NO | Davies, 1970 |
| Site 46 | NOT FOUND | NOT FOUND | SA | SURV |  | NO | Davies, 1970 |
| Site 50 Estuary Umfazazaan River South | -30.53 | 30.6 | SA | SURV |  | NO | Davies, 1970 |
| Site 54 60m beach | NOT FOUND | NOT FOUND | SA | SURV |  | NO | Davies, 1970 |
| Site 59 | NOT FOUND | NOT FOUND | SA | SURV |  | NO | Davies, 1970 |
| Site 68 | NOT FOUND | NOT FOUND | SA | SURV |  | NO | Davies, 1970 |
| Site 710 | NOT FOUND | NOT FOUND | SA | SURV |  | NO | Davies, 1970 |
| Site 711 | NOT FOUND | NOT FOUND | SA | SURV |  | NO | Davies, 1970 |
| Site 714/715 Mkumi Game Reserve | NOT FOUND | NOT FOUND | SA | SURV |  | NO | Davies, 1970 |
| Site 719 Eukuseni West Dip.Station | NOT FOUND | NOT FOUND | SA | SURV |  | NO | Davies, 1970 |
| Site 720 Muniwana | NOT FOUND | NOT FOUND | SA | SURV |  | NO | Davies, 1970 |
| Site 722 Mzeneni Sizal-Mill | NOT FOUND | NOT FOUND | SA | SURV |  | NO | Davies, 1970 |
| Site 814 Hluhluuwe River Bridge Railway | -28.022 | 32.28 | SA | SURV |  | NO | Davies, 1970 |
| Site 831 Umfrosi Motel | NOT FOUND | NOT FOUND | SA | SURV |  | NO | Davies, 1970 |
| Site 910 | NOT FOUND | NOT FOUND | SA | SURV |  | NO | Davies, 1970 |
| Site 93 | NOT FOUND | NOT FOUND | SA | SURV |  | NO | Davies, 1970 |
| Site 936 Umhloti Mouth | -29.561 | 31.123 | SA | SURV |  | NO | Davies, 1970 |
| Site 94 | NOT FOUND | NOT FOUND | SA | SURV |  | NO | Davies, 1970 |
| Site 941 Redhill Brickpit 64m beach | -29.76 | 31.03 | SA | SURV |  | NO | Davies, 1970 |
| Site 942 | NOT FOUND | NOT FOUND | SA | SURV |  | NO | Davies, 1970 |
| Site 948 - Clare Road 66m beach | -29.805 | 30.975 | SA | SURV |  | NO | Davies, 1970 |
| Site 966 Dunns Grant, above Lamont | NOT FOUND | NOT FOUND | SA | SURV |  | NO | Davies, 1970 |
| Site 967 Umlaas Bridge | -29.834 | 30.524 | SA | SURV |  | NO | Davies, 1970 |
| Skoongele | -34.281878 | 21.401073 | SA | SURV |  | NO | Breuil, 1948; Davies, 1971 |
| Stanford | -34.45 | 19.45 | SA | UNK |  | NO | Keller, Rudner, Deacon, Davies, Clark 1967 |
| Stillbaai | -34.366269 | 21.434648 | SA | EXC |  | NO | Davies, 1971 |
| Swarte Koppen | -33.831384 | 25.583072 | SA | UNK |  | NO | Breuil, 1948 |
| Tygerberg Hill | -33.866667 | 18.5833333 | SA | UNK |  | NO | Keller, Rudner, Deacon, Davies, Clark 1967 |
| Umbogintwini Valley | -30.083333 | 30.9166667 | SA | UNK |  | NO | Keller, Rudner, Deacon, Davies, Clark 1967 |
| Umgababa | -30.1279 | 30.8373 | SA | EXC |  | NO | Davies, 1982 |
| Umhala's Kop | -33 | 27.9166667 | SA | UNK |  | NO | Keller, Rudner, Deacon, Davies, Clark 1967 |
| Umhlatuzana | -30 | 30.9166667 | SA | UNK |  | NO | Davies, 1954 |
| Umhlatuzana | -30 | 30.9166667 | SA | SURV |  | NO | Davies, 1970 |
| Umhlatuzana | -30 | 30.9166667 | SA | UNK |  | NO | Davies, 1954 |
| Umlaaas Bridge | -29.741751 | 30.509801 | SA | SURV |  | NO | Davies, 1970 |
| Umlaas | -29.5 | 31.1666667 | SA | UNK |  | NO | Keller, Rudner, Deacon, Davies, Clark 1967 |
| Umlozi Reserve, Umbogintwini +190 m | -30 | 30.9166667 | SA | UNK |  | NO | Keller, Rudner, Deacon, Davies, Clark 1967 |
| Umvoti Reserve "Near Mouth" | -29.384363 | 31.3351275 | SA | UNK |  | NO | Keller, Rudner, Deacon, Davies, Clark 1967 |
| Uvongo Valley | -30.831865 | 30.38487 | SA | UNK |  | NO | Keller, Rudner, Deacon, Davies, Clark 1967 |
| Vanrhynsdorp | -31.651534 | 18.575403 | SA | SURV |  | NO | Davies, 1973 |
| Victoria Park - Mossel Bay | -34.07668 | 22.150364 | SA | SURV |  | NO | Davies, 1971 |
| Viswater | -31.690009 | 18.187846 | SA | SURV |  | NO | Davies, 1973 |
| Vondeling | -34.242031 | 20.880435 | SA | SURV |  | NO | Davies, 1971 |
| Wallekraal | -30.364129 | 17.502678 | SA | SURV |  | NO | Davies, 1973 |
| Witsand | -34.016667 | 18.35 | SA | UNK |  | NO | Keller, Rudner, Deacon, Davies, Clark 1967 |
| Wittedrift | -34.016667 | 23.3333333 | SA | UNK |  | NO | Keller, Rudner, Deacon, Davies, Clark 1967 |
| Wynberg | -34.016667 | 18.4666667 | SA | UNK |  | NO | Keller, Rudner, Deacon, Davies, Clark 1967 |
| Barra do Cuanza | -9.328898 | 13.147219 | ANG | UNK |  | NO | Clark, 1966; Ervedosa, 1980 |
| Giraul | -15.083333 | 12.15 | ANG | UNK |  | NO | de Almeida, Camarate in Clark, 1967 |
| Benguela 2 | -12.652203 | 13.326995 | ANG | UNK |  | NO | Pinto, 1988 |
| Benguela 2 | -12.64693 | 13.329816 | ANG | UNK |  | NO | Pinto, 1988 |
| Benguela 3 | -12.651205 | 13.323546 | ANG | UNK |  | NO | Pinto, 1988 |
| Benguela 4 | -12.652031 | 13.320843 | ANG | UNK |  | NO | Pinto, 1988 |
| Benguela 5 | -12.645193 | 13.34615 | ANG | UNK |  | NO | Pinto, 1988 |
| Canjongo | -14.092216 | 12.362765 | ANG | UNK |  | NO | Ramos, 1982 |
| Dungo IV | -12.667252 | 13.152996 | ANG | EXC |  | YES | Clark, 1966; Ervedosa, 1967; Gutierrez & Benjamim, 2019; Pinto, 1992 |
| Dungo V | -12.663718 | 13.166743 | ANG | EXC |  | YES | Gutierrez et al., 2001 |
| Dungo XII | -12.673415 | 13.151208 | ANG | EXC |  | ESTIM | Unpublished Record of the MNAB |
| Dungo XIII | -12.680028 | 13.181135 | ANG | UNK |  | ESTIM | Gutierrez et al., 2018 |
| Limagens | -13.687244 | 12.532963 | ANG | UNK |  | NO | Clark, 1966 |
| Lobito Sul | -12.388715 | 13.551352 | ANG | UNK |  | NO | Ramos, 1982 |
| Macaca | -12.696288 | 13.130261 | ANG | SURV | MPT | ESTIM | Feio, 1960 |
| Moçamedes/Namibe (32km toward Lucira) | -14.037179 | 12.427425 | ANG | UNK |  | NO | de Almeida, Camarate, Clark 1967 |
| Moçamedes/Namibe (40km toward Porto Alexandre) | -15.5 | 12.0833333 | ANG | UNK |  | NO | de Almeida, Camarate, Clark 1967 |
| Mormolo 1 | -12.6403 | 13.241063 | ANG | UNK | MPT | ESTIM | Pinto, 1988 |
| Mormolo 2 | -12.6404 | 13.241064 | ANG | UNK | MPT | ESTIM | Pinto, 1988 |
| Mormolo 3 | -12.631113 | 13.250494 | ANG | UNK | MPT | ESTIM | Pinto, 1988 |
| Mormolo 4 | -12.615878 | 13.259222 | ANG | UNK | MPT | ESTIM | Pinto, 1988 |
| Mormolo 5 | -12.617543 | 13.223679 | ANG | UNK | MPT | ESTIM | Pinto, 1988 |
| Morro do Sombreiro | -12.5825 | 13.299883 | ANG | UNK |  | NO | Ervedosa, 1980; Neto, 1956 |
| Octavio's Hand | -15.5 | 12.0833333 | ANG | UNK |  | NO | de Almeida, Camarate, Clark 1967 |
| Palmeirinhas | -8.719546 | 13.107422 | ANG | EXC | MPT | ESTIM | Clark, 1966 |
| Pima | -12.662 | 13.289 | ANG | SURV | MPT | ESTIM | Unpublished Record of the MNAB |
| Ponta do Mexilhao | -15.189786 | 12.128134 | ANG | UNK |  | NO | Gonçalves dos Santos, 1971 |
| Ponta do Sombreiro | -12.5825 | 13.299883 | ANG | UNK |  | NO | Neto, 1956; Soares de Carvalho, 1960 |
| Ponta Giraul -Mocamedes | -15.135 | 12.1119 | ANG | UNK |  | NO | Ramos, 1982 |
| Ponta Negra | -14.633253 | 12.293149 | ANG | UNK |  | NO | Gonçalves dos Santos, 1971 |
| Porto Alexandre (39km vers Moçamedes) | -15.5 | 12.0833333 | ANG | UNK |  | NO | de Almeida, Camarate, Clark 1967 |
| Porto Alexandre (Tomboa) | -15.797005 | 11.862507 | ANG | UNK |  | NO | Clark, 1966 |
| Punta das Vacas | -12.590879 | 13.279541 | ANG | UNK |  | NO | Clark, 1966 |
| Punta do Mexilhao | -15.189786 | 12.128134 | ANG | UNK |  | NO | Gonçalves dos Santos, 1971 |
| Punta Giraul | -15.135 | 12.1119 | ANG | UNK |  | NO | Ramos, 1982 |
| Punta Negra | -14.633253 | 12.293149 | ANG | UNK |  | NO | Gonçalves dos Santos, 1971 |
| Rua Bito Godins (Av, Lenine) | -8.820125 | 13.235739 | ANG | MIN |  | NO | Clark, 1966 |
| Sao Nicolau C | -13.972469 | 12.423881 | ANG | UNK |  | NO | Allchin, 1964; Ramos, 1982 |
| Uiche | -31.047 | 30.22 | ANG | SURV | MPT | ESTIM | Unpublished Record of the MNAB |
| Buzi Company | -19.883333 | 34.5 | MOZ | UNK |  | NO | Barradas in Clark 1967 |
| Ponta Maona | -26.133333 | 32.5833333 | MOZ | UNK |  | NO | Barradas in Clark 1967 |
| Revez Duarte | -26.032487 | 32.398418 | MOZ | UNK |  | NO | Barradas, 1965 |
| Cunene Mouth | -17.19 | 11.92 | NAM | UNK |  | NO | Kinahan comm. pers. |
| Gemsbok (several localities) | -28.606256 | 16.429395 | NAM | MIN | 400-700 ka | ESTIM | Corvinus, 1983, 1985 |
| Lüderitz | -26.649254 | 15.161015 | NAM | MIN |  | NO | Unpublished Record of the NMN |
| Mowe Bay | -19.373445 | 12.708545 | NAM | SURV |  | NO | Hardaker, 2005 |
| Namib IV | -23.783333 | 15.3333333 | NAM | SURV | 400-700 ka | ESTIM | Shackley, 1980 |
| Narabeb | -23.743843 | 14.949333 | NAM | SURV |  | NO | Shackley, 1984 |
| Narabeb West | -23.683333 | 14.7833333 | NAM | SURV |  | NO | Shackley, 1984 |
| Omaruru Mouth | -22.095369 | 14.258904 | NAM | UNK |  | NO | Korn & Martin, 1957 |
| Orabis River, Brandberg | -21.133333 | 14.5166667 | NAM | UNK |  | NO | MacCalman, Viereck, Scherz, Sydow, Rudner, Fock, Clark 1967 |
| Orange Mouth | -28.591842 | 16.446596 | NAM | MIN |  | NO | Davies, 1973 |
| SWA 56 | -21.438889 | 13.838889 | NAM | SURV |  | NO | Davies, 1973 |
| SWA30 | -22.28611 | 14.411111 | NAM | SURV |  | NO | Davies, 1973 |
| SWA49 | -21.801562 | 14.028072 | NAM | SURV |  | NO | Davies, 1973 |
| Swakop Mouth | -22.691005 | 14.525518 | NAM | UNK |  | NO | Korn & Martin, 1957 |
| Tsondab Route | -23.69 | 15.22 | NAM | SURV |  | NO | Shackley, 1984 |
| Uubvley | -28.157206 | 15.907714 | NAM | MIN |  | NO | Corvinus, 1983 |

**References**

Allchin, B. (1964). A preliminary survey of stone age sites of the Serra-Abaixo (South West Angola). *Estudos Sobre Pré-Historia Do Utramar Português*, *2*(50), 81–99.

Barradas, L. (1965). Cronologia da Beira Mar do Sul de Mocambique. *Mem. Inst. Cient. Mozamb*, *7*, 23–35.

Braun, D. R., Levin, N. E., Stynder, D., Herries, A. I. R., Archer, W., Forrest, F., Roberts, D. L., Bishop, L. C., Matthews, T., Lehmann, S. B., Pickering, R., & Fitzsimmons, K. E. (2013). Mid-Pleistocene Hominin occupation at Elandsfontein, Western Cape, South Africa. *Quaternary Science Reviews*, *82*, 145–166. https://doi.org/10.1016/j.quascirev.2013.09.027

Breuil, H. (1945). The old palaeolithic age in relation to quaternary sea-leaves along the southern coast of Africa. *South African Journal of Science*, *41*(07), 361–374.

Breuil, H. (1948). *MS. report on Sundays River Survey*. University of Witwatersrand; File B 17/7.

Case, S. (2018). *Archaeological impact assessment proposed abalone holding and processing facility on the Farm Rooiklippe, near Kleinzee, Northern Cape—Report prepared for Anchor Environmental Consultants* (p. 28). ACRM.

Clark, J. D. (1966). *The distribution of prehistoric culture in Angola*. Companhia de Diamantes de Angola (Diamang).

Clark, J. D. (1967). *Atlas of African prehistory*. University of Chicago Press.

Conard, N. J. (2003). Handaxes on the landscape and the reconstruction of Paleolithic settlement patterns. *Erkenntnisjäger: Kultur Und Umwelt Des Frühen Menschen. Festschrift Für Dietrich Mania*, *57*, 123–144.

Cooke, H. B. S. (1941). *A preliminary survey of the Quaternary period in southern Africa.* Union of South Africa Bureau of Archaeology.

Corvinus, G. (1983). *The raised beaches of the west coast of South West Africa/Namibia: An interpretation of their archaeological and palaeontological data* (München, Vol. 5). C.H. Beck.

Corvinus, G. (1985). An Acheulian industry within raised beach complex of the CDM concession area, S.W. Africa (Namibia). *Quartär*, 183–189.

Davies, O. (1954). The Beaches at Umhlatuzana, near Durban. *The South African Archaeological Bulletin*, *9*(34), 60–63. JSTOR. https://doi.org/10.2307/3887092

Davies, O. (1970). Pleistocene beaches of Natal. *Annals of the Natal Museum*, *20*(2), 403–442.

Davies, O. (1971). Pleistocene shorelines in the southern and south-eastern Cape Province (Part 1). *Annals of the Natal Museum*, *21*(1), 183–223. https://doi.org/10.10520/AJA03040798_676

Davies, O. (1972). Pleistocene shorelines in the southern and south-eastern Cape Province (Part 2). *Annales of the Natal Museum*, 225–279.

Davies, O. (1973). Pleistocene shorelines in the Western Cape and South-West Africa. *Annals of the Natal Museum*, *21*(3), 719–765. https://doi.org/10.10520/AJA03040798_644

Davies, O. (1982). The Palaeolithic sequence at Umgababa ilmenite diggings. *Annals of the Natal Museum*, *25*(1), 41–59.

Davies, O., & Walsh, C. (1955). Raised beaches and associated stone-age material in Namaqualand. *South African Journal of Science*, *51*(9), 277.

Ervedosa, C. (1967). *A estação paleolítica da Baía Farta (Angola)*. Imprensa Portuguesa.

Ervedosa, C. (1980). *Arqueologia angolana* (Ministeiro da Cultura Angolana). Edições 70.

Feathers, J. K. (2002). Luminescence Dating in Less Than Ideal Conditions: Case Studies from Klasies River Main Site and Duinefontein, South Africa. *Journal of Archaeological Science*, *29*(2), 177–194. https://doi.org/10.1006/jasc.2001.0685

Feio, M. (1960). As praias levantadas da regiao do Lobito e da Baia Farta. *Revisao Da Junta de Investigacao Do Ultramar Gaxia de Orto*, *8*(2), 357–370.

Fisher, E. C., Albert, R.-M., Botha, G., Cawthra, H. C., Esteban, I., Harris, J., Jacobs, Z., Jerardino, A. M. S., Marean, C. W., Neumann, F. H., Pargeter, J., Poupart, M., & Venter, J. (2013). *Archaeological Reconnaissance for Middle Stone Age Sites Along the Pondoland Coast, South Africa*. https://doi.org/10.4207/PA.2013.ART82

Gonçalves dos Santos, V. (1971). Morfologia descritiva de choppers, chopping-tools e seixos afeiçoados da Ponta Negra (Moçâmedes—Angola). *O Arqueologo Portugês*, *3*, 7–17.

Granger, D. E., Gibbon, R. J., Kuman, K., Lotter, M. G., & Erlanger, E. (2013). Isochron burial dating method and application to an Earlier Stone Age chronosequence on the Sundays River, South Africa. *Poster Presented at the Association of Southern African Professional Archaeologists Biennial Conference, Gaborone, Botswana*.

Gutierrez, M., & Benjamim, M. H. (2019). *Recherches Archéologiques à Baia Farta (Benguela-Angola) / Pesquisas Arqueológicicas na Baia Farta (Benguela-Angola)* (L’Harmattan).

Gutierrez, M., Fançony, R., & Benjamim, M. H. (2018). Découverte d’un important site archéologique à Baia Farta (Angola). *Afrique: Archéologie & Arts*, *14*, 105–106.

Gutierrez, M., Guérin, C., Benjamim, M. H., & Piedade da Jesus, M. (2001). Exploitation d’un grand cétacé au Paléolithique ancien: Le site de Dungo V à Baia Farta (Benguela, Angola). *Comptes Rendus de l’Académie Des Sciences - Series IIA - Earth and Planetary Science*, *332*(5), 357–362. https://doi.org/10.1016/S1251-8050(01)01518-X

Hardaker, T. (2005). The Namibia Palaeolithic Field Research Project (Nampal) 2002. *The South African Archaeological Bulletin*, *60*(181), 20–23. JSTOR.

Kandel, A. W., Walker, S. J., & Conard, N. J. (2006). Near-coastal settlement dynamics at the Anyskop Blowout, an archaeological locality at Langebaanweg, South Africa. *African Natural History*, *2*, 186.

Keller, C. M. (1973). *Montagu Cave in prehistory: A descriptive analysis* (University of California Anthropology). Records.

Korn, H., & Martin, H. (1957). The Pleistocene in South West Africa. *Proceedings of the 3rd Pan-African Congress on Prehistory*, 14–22.

Laidler, P. W. (1947). The Evolution of Middle Palaeolithic Technique at Geelhout, Near Kareedouw, in the Southern Cape. *Transactions of the Royal Society of South Africa*, *31*(3), 283–313. https://doi.org/10.1080/00359194709518941

Lotter, M. G. (2016). *The archaeology of the lower Sundays River Valley, Eastern Cape Province, South Africa: An assessment of Earlier Stone Age alluvial terrace sites* [PhD Thesis]. University of Witswatersrand.

Lotter, M. G., & Kuman, K. (2018a). Atmar and Bernol farms: New Acheulean sites in the lower Sundays River Valley, Eastern Cape province, South Africa. *South African Archaeological Bulletin*, *73*(207), 64.

Lotter, M. G., & Kuman, K. (2018b). The Acheulean in South Africa, with announcement of a new site (Penhill Farm) in the lower Sundays River Valley, Eastern Cape Province, South Africa. *Quaternary International*, *480*, 43–65.

Macfarlane, D. P. (1935). Evidence in support of a pre-Chellean culture from the East London Clays. *South African Journal of Science*, *32*(07), 537–550.

McNabb, J., Binyon, F., & Hazelwood, L. (2004). The Large Cutting Tools from the South African Acheulean and the Question of Social Traditions. *Current Anthropology*, *45*(5), 653–677. JSTOR. https://doi.org/10.1086/423973

Mortelmans, G. (1945). Plages soulevées à industries lithiques de la région de Keurbooms River, District de Knysna. *South African Journal of Science*, *61*, 375–396.

Neto, M. (1956). *Nota acerca da estratigrafia da Baia Farta*. Serviços de Geologia e Minas.

Pinto, L. J. M. (1988). Le Musée National d’Archéologie de Benguela (Angola): Bilan des premiers travaux: 1979-1987. *Nsi*, *3*, 5–14.

Pinto, L. J. M. (1992). Arqueologia da faixa sedimentar de Benguela. A Idade da pedra e do ferro. Contributo para o seu estudo. *Leba. Estudos de Pré-História e Arqueología*, *7*, 203–220.

Ramos, M. (1982). Le Paléolithique du Sud-ouest de l’Angola, vue d’ensemble. *Leba. Estudos de Pré-Historia e Arquelogia Lisboa*, *5*, 43–52.

Shackley, M. (1980). An Acheulean industry with Elephas recki fauna from Namib IV, South West Africa (Namibia). *Nature*, *284*(5754), 340–341. https://doi.org/10.1038/284340a0

Shackley, M. (1984). Off-site distribution and artifact visibility in the central Namib Desert, South West Africa / Namibia. *Cimbebasia, Series B*, *4*(5), 55–88.

Singer, R., & Wymer, J. (1968). Archaeological investigations at the Saldanha skull site in South Africa. *The South African Archaeological Bulletin*, *23*(91), 63–74.

Soares de Carvalho, G. (1960). Alguna problemas dos terracos quater- narias de litoral de Angola. *Boletim Do Serviço de Geologia e Minas de Angola*, *2*, 5–15.

Thompson, E. (2009). Acheulean artifact accumulation and early hominin land use, Garden Route Casino Road, Pinnacle Point, South Africa. *Geoarchaeology: An International Journal*, *24*(4), 402–428.
