# Supplementary material for: Evidence for Earlier Stone Age ‘coastal use’: The site of Dungo IV, Benguela Province, Angola
Source: PLoS One. 2023 Feb 24;18(2):e0278775. doi: 10.1371/journal.pone.0278775 (PMC9955982; doi:10.1371/journal.pone.0278775)
Supplement: S2 Table — Length, maximal width, maximal thickness, elongation and fineness of the shaped tools from Gemsbok, Penhill Farm, Dungo IV, Namib IV, Elandsfontein and Cape Hangklip. (DOCX) [file pone.0278775.s002.docx]

**Supplementary Information – File 2**

This table provide the entire set of metrical data and indices result used for the inter-site comparison of shaped tools from Gemsbok, Penhill Farm, Dungo IV, Namib IV, Elandsfontein and Cape Hangklip. All the pieces have been measured by authors except the oens from Elandsfontein and Cape Hangklip. For both sites the data have been provided by Marshall et al., 2002. <https://archaeologydataservice.ac.uk/archives/view/bifaces/overview.cfm>

| Site | Length | Maximal Width | Maximal Thickness | Length/Width | Width/Thickness | Length/Thickness |
| --- | --- | --- | --- | --- | --- | --- |
| Gemsbok | 13.7 | 9.7 | 5.5 | 1.412371134 | 1.763636364 | 2.490909091 |
| Gemsbok | 19.7 | 9.2 | 6.1 | 2.141304348 | 1.508196721 | 3.229508197 |
| Gemsbok | 13.2 | 10.6 | 4.8 | 1.245283019 | 2.208333333 | 2.75 |
| Gemsbok | 6.7 | 4.8 | 4.5 | 1.395833333 | 1.066666667 | 1.488888889 |
| Gemsbok | 15 | 8.7 | 3.5 | 1.724137931 | 2.485714286 | 4.285714286 |
| Gemsbok | 23.6 | 11.3 | 6.5 | 2.088495575 | 1.738461538 | 3.630769231 |
| Gemsbok | 19 | 7.7 | 5.2 | 2.467532468 | 1.480769231 | 3.653846154 |
| Gemsbok | 14.3 | 7.7 | 5 | 1.857142857 | 1.54 | 2.86 |
| Gemsbok | 13.3 | 9.5 | 4.4 | 1.4 | 2.159090909 | 3.022727273 |
| Gemsbok | 15.5 | 9 | 7 | 1.722222222 | 1.285714286 | 2.214285714 |
| Gemsbok | 14 | 8.3 | 5.4 | 1.686746988 | 1.537037037 | 2.592592593 |
| Gemsbok | 13.5 | 9 | 6.9 | 1.5 | 1.304347826 | 1.956521739 |
| Gemsbok | 13.1 | 8.3 | 3.5 | 1.578313253 | 2.371428571 | 3.742857143 |
| Gemsbok | 8.3 | 5.3 | 3.7 | 1.566037736 | 1.432432432 | 2.243243243 |
| Gemsbok | 16.9 | 8.7 | 5.6 | 1.942528736 | 1.553571429 | 3.017857143 |
| Gemsbok | 16.2 | 11.4 | 5.2 | 1.421052632 | 2.192307692 | 3.115384615 |
| Gemsbok | 16.3 | 7.5 | 5 | 2.173333333 | 1.5 | 3.26 |
| Gemsbok | 13 | 7.4 | 5 | 1.756756757 | 1.48 | 2.6 |
| Gemsbok | 9 | 6.7 | 4.5 | 1.343283582 | 1.488888889 | 2 |
| Gemsbok | 11.1 | 6.7 | 5.3 | 1.656716418 | 1.264150943 | 2.094339623 |
| Gemsbok | 14.2 | 9 | 5.6 | 1.577777778 | 1.607142857 | 2.535714286 |
| Gemsbok | 13.5 | 8.5 | 5.5 | 1.588235294 | 1.545454545 | 2.454545455 |
| Gemsbok | 14 | 11.4 | 5 | 1.228070175 | 2.28 | 2.8 |
| Gemsbok | 14.5 | 11 | 6.6 | 1.318181818 | 1.666666667 | 2.196969697 |
| Gemsbok | 17.8 | 11.5 | 5 | 1.547826087 | 2.3 | 3.56 |
| Gemsbok | 21.1 | 10.3 | 8 | 2.048543689 | 1.2875 | 2.6375 |
| Gemsbok | 13.5 | 9.1 | 4 | 1.483516484 | 2.275 | 3.375 |
| Gemsbok | 17 | 10.7 | 6.7 | 1.588785047 | 1.597014925 | 2.537313433 |
| Gemsbok | 14.7 | 9 | 5.1 | 1.633333333 | 1.764705882 | 2.882352941 |
| Gemsbok | 14 | 8.8 | 5.4 | 1.590909091 | 1.62962963 | 2.592592593 |
| Gemsbok | 13 | 8.6 | 5.1 | 1.511627907 | 1.68627451 | 2.549019608 |
| Gemsbok | 11 | 7.9 | 4.6 | 1.392405063 | 1.717391304 | 2.391304348 |
| Gemsbok | 11.8 | 6.3 | 3 | 1.873015873 | 2.1 | 3.933333333 |
| Gemsbok | 13.1 | 8.2 | 3 | 1.597560976 | 2.733333333 | 4.366666667 |
| Gemsbok | 13.1 | 9.5 | 5 | 1.378947368 | 1.9 | 2.62 |
| Gemsbok | 14 | 9.6 | 5 | 1.458333333 | 1.92 | 2.8 |
| Gemsbok | 17 | 8 | 5 | 2.125 | 1.6 | 3.4 |
| Gemsbok | 15.2 | 9.8 | 6.1 | 1.551020408 | 1.606557377 | 2.491803279 |
| Gemsbok | 10.7 | 7.4 | 5.5 | 1.445945946 | 1.345454545 | 1.945454545 |
| Gemsbok | 15.4 | 10 | 4.5 | 1.54 | 2.222222222 | 3.422222222 |
| Gemsbok | 18.5 | 9.3 | 5.1 | 1.989247312 | 1.823529412 | 3.62745098 |
| Gemsbok | 22.9 | 13 | 5.8 | 1.761538462 | 2.24137931 | 3.948275862 |
| Gemsbok | 13 | 8.4 | 4.9 | 1.547619048 | 1.714285714 | 2.653061224 |
| Gemsbok | 17 | 8.7 | 7 | 1.954022989 | 1.242857143 | 2.428571429 |
| Gemsbok | 16.5 | 8.5 | 4.7 | 1.941176471 | 1.808510638 | 3.510638298 |
| Gemsbok | 20.1 | 8 | 6.6 | 2.5125 | 1.212121212 | 3.045454545 |
| Gemsbok | 17 | 12.5 | 5.6 | 1.36 | 2.232142857 | 3.035714286 |
| Gemsbok | 14.3 | 9.1 | 5.2 | 1.571428571 | 1.75 | 2.75 |
| Gemsbok | 17 | 10.6 | 5 | 1.603773585 | 2.12 | 3.4 |
| Gemsbok | 17.9 | 11 | 5.2 | 1.627272727 | 2.115384615 | 3.442307692 |
| Gemsbok | 9.1 | 7.5 | 3.5 | 1.213333333 | 2.142857143 | 2.6 |
| Gemsbok | 10.6 | 6.7 | 3.5 | 1.582089552 | 1.914285714 | 3.028571429 |
| Gemsbok | 14.8 | 11.1 | 4.3 | 1.333333333 | 2.581395349 | 3.441860465 |
| Gemsbok | 10.7 | 8.5 | 4.6 | 1.258823529 | 1.847826087 | 2.326086957 |
| Gemsbok | 15.7 | 10.2 | 6 | 1.539215686 | 1.7 | 2.616666667 |
| Gemsbok | 11.5 | 8 | 3.5 | 1.4375 | 2.285714286 | 3.285714286 |
| Gemsbok | 14.5 | 9.7 | 4.7 | 1.494845361 | 2.063829787 | 3.085106383 |
| Gemsbok | 12.3 | 9.6 | 4.2 | 1.28125 | 2.285714286 | 2.928571429 |
| Gemsbok | 15.4 | 10 | 5 | 1.54 | 2 | 3.08 |
| Gemsbok | 18.2 | 8.7 | 4 | 2.091954023 | 2.175 | 4.55 |
| Gemsbok | 11.7 | 8.1 | 4.7 | 1.444444444 | 1.723404255 | 2.489361702 |
| Gemsbok | 16.9 | 15.6 | 5.5 | 1.083333333 | 2.836363636 | 3.072727273 |
| Gemsbok | 16.5 | 8.2 | 4 | 2.012195122 | 2.05 | 4.125 |
| Gemsbok | 13 | 8.8 | 2.7 | 1.477272727 | 3.259259259 | 4.814814815 |
| Gemsbok | 18 | 10.5 | 4.5 | 1.714285714 | 2.333333333 | 4 |
| Gemsbok | 16 | 10.2 | 5.6 | 1.568627451 | 1.821428571 | 2.857142857 |
| Gemsbok | 14.2 | 7.7 | 3.7 | 1.844155844 | 2.081081081 | 3.837837838 |
| Gemsbok | 10.2 | 6.7 | 3.4 | 1.52238806 | 1.970588235 | 3 |
| Gemsbok | 13.5 | 7.3 | 4.7 | 1.849315068 | 1.553191489 | 2.872340426 |
| Gemsbok | 9.3 | 6 | 3 | 1.55 | 2 | 3.1 |
| Gemsbok | 14.1 | 8.9 | 5.7 | 1.584269663 | 1.561403509 | 2.473684211 |
| Gemsbok | 20.5 | 14 | 8.5 | 1.464285714 | 1.647058824 | 2.411764706 |
| Gemsbok | 14 | 7.5 | 6.5 | 1.866666667 | 1.153846154 | 2.153846154 |
| Gemsbok | 16.1 | 12.2 | 4.4 | 1.319672131 | 2.772727273 | 3.659090909 |
| Gemsbok | 10.7 | 8.7 | 4.2 | 1.229885057 | 2.071428571 | 2.547619048 |
| Gemsbok | 13 | 8.3 | 4.7 | 1.56626506 | 1.765957447 | 2.765957447 |
| Gemsbok | 11.9 | 4.5 | 3.3 | 2.644444444 | 1.363636364 | 3.606060606 |
| Gemsbok | 12.5 | 9.5 | 5.1 | 1.315789474 | 1.862745098 | 2.450980392 |
| Gemsbok | 16.5 | 10.2 | 5.7 | 1.617647059 | 1.789473684 | 2.894736842 |
| Gemsbok | 15.1 | 8 | 3.9 | 1.8875 | 2.051282051 | 3.871794872 |
| Gemsbok | 15 | 9 | 5.2 | 1.666666667 | 1.730769231 | 2.884615385 |
| Gemsbok | 12.4 | 6.3 | 3.9 | 1.968253968 | 1.615384615 | 3.179487179 |
| Gemsbok | 17 | 11.7 | 3.7 | 1.452991453 | 3.162162162 | 4.594594595 |
| Gemsbok | 12.2 | 7.4 | 2.9 | 1.648648649 | 2.551724138 | 4.206896552 |
| Gemsbok | 12.9 | 7.5 | 6 | 1.72 | 1.25 | 2.15 |
| Gemsbok | 12.3 | 7.3 | 3.8 | 1.684931507 | 1.921052632 | 3.236842105 |
| Gemsbok | 12 | 8.6 | 3.8 | 1.395348837 | 2.263157895 | 3.157894737 |
| Gemsbok | 16.3 | 8.5 | 4.5 | 1.917647059 | 1.888888889 | 3.622222222 |
| Gemsbok | 16 | 7.9 | 5.5 | 2.025316456 | 1.436363636 | 2.909090909 |
| Gemsbok | 11.5 | 8.5 | 3.2 | 1.352941176 | 2.65625 | 3.59375 |
| Gemsbok | 13 | 9 | 7 | 1.444444444 | 1.285714286 | 1.857142857 |
| Gemsbok | 15 | 10.7 | 6 | 1.401869159 | 1.783333333 | 2.5 |
| Gemsbok | 15 | 9.1 | 7 | 1.648351648 | 1.3 | 2.142857143 |
| Gemsbok | 12.2 | 8.5 | 4.2 | 1.435294118 | 2.023809524 | 2.904761905 |
| Gemsbok | 15.5 | 9.6 | 4.8 | 1.614583333 | 2 | 3.229166667 |
| Gemsbok | 17.7 | 8.9 | 5.2 | 1.988764045 | 1.711538462 | 3.403846154 |
| Gemsbok | 13 | 8.8 | 4.4 | 1.477272727 | 2 | 2.954545455 |
| Gemsbok | 21 | 8.9 | 8 | 2.359550562 | 1.1125 | 2.625 |
| Gemsbok | 10.5 | 6.4 | 3.2 | 1.640625 | 2 | 3.28125 |
| Gemsbok | 18.7 | 8.7 | 6 | 2.149425287 | 1.45 | 3.116666667 |
| Gemsbok | 15.5 | 10.9 | 4 | 1.422018349 | 2.725 | 3.875 |
| Gemsbok | 11 | 6.9 | 3.1 | 1.594202899 | 2.225806452 | 3.548387097 |
| Gemsbok | 16.5 | 9.7 | 4 | 1.701030928 | 2.425 | 4.125 |
| Gemsbok | 12 | 7.2 | 5 | 1.666666667 | 1.44 | 2.4 |
| Gemsbok | 12.6 | 8.5 | 5.5 | 1.482352941 | 1.545454545 | 2.290909091 |
| Gemsbok | 10.2 | 6.2 | 6 | 1.64516129 | 1.033333333 | 1.7 |
| Gemsbok | 14 | 6.8 | 6.8 | 2.058823529 | 1 | 2.058823529 |
| Gemsbok | 14 | 7.5 | 5.5 | 1.866666667 | 1.363636364 | 2.545454545 |
| Gemsbok | 17.3 | 8.9 | 5.3 | 1.943820225 | 1.679245283 | 3.264150943 |
| Gemsbok | 14.3 | 9 | 6 | 1.588888889 | 1.5 | 2.383333333 |
| Gemsbok | 15.5 | 9.5 | 5.8 | 1.631578947 | 1.637931034 | 2.672413793 |
| Penhill Farm | 8.3 | 7.7 | 4 | 1.077922078 | 1.925 | 2.075 |
| Penhill Farm | 13.2 | 9.3 | 5 | 1.419354839 | 1.86 | 2.64 |
| Penhill Farm | 10.6 | 7.7 | 3.5 | 1.376623377 | 2.2 | 3.028571429 |
| Penhill Farm | 7.4 | 4.4 | 2.6 | 1.681818182 | 1.692307692 | 2.846153846 |
| Penhill Farm | 5.2 | 3.5 | 2.2 | 1.485714286 | 1.590909091 | 2.363636364 |
| Penhill Farm | 9.7 | 6.5 | 2.2 | 1.492307692 | 2.954545455 | 4.409090909 |
| Penhill Farm | 8.2 | 5.5 | 2.2 | 1.490909091 | 2.5 | 3.727272727 |
| Penhill Farm | 10 | 6.9 | 3 | 1.449275362 | 2.3 | 3.333333333 |
| Penhill Farm | 13 | 8 | 4.4 | 1.625 | 1.818181818 | 2.954545455 |
| Penhill Farm | 9.8 | 7.2 | 4 | 1.361111111 | 1.8 | 2.45 |
| Penhill Farm | 10.8 | 5.8 | 4 | 1.862068966 | 1.45 | 2.7 |
| Penhill Farm | 11 | 6.8 | 3 | 1.617647059 | 2.266666667 | 3.666666667 |
| Penhill Farm | 9 | 6 | 2 | 1.5 | 3 | 4.5 |
| Penhill Farm | 10 | 4.2 | 3 | 2.380952381 | 1.4 | 3.333333333 |
| Penhill Farm | 8.2 | 4.9 | 2.4 | 1.673469388 | 2.041666667 | 3.416666667 |
| Penhill Farm | 11.4 | 6.9 | 4 | 1.652173913 | 1.725 | 2.85 |
| Penhill Farm | 13 | 8.5 | 4.3 | 1.529411765 | 1.976744186 | 3.023255814 |
| Penhill Farm | 19 | 10.4 | 4.2 | 1.826923077 | 2.476190476 | 4.523809524 |
| Penhill Farm | 11.1 | 7.6 | 5.4 | 1.460526316 | 1.407407407 | 2.055555556 |
| Penhill Farm | 14 | 8 | 4 | 1.75 | 2 | 3.5 |
| Penhill Farm | 14.5 | 8.7 | 4.5 | 1.666666667 | 1.933333333 | 3.222222222 |
| Penhill Farm | 11.5 | 6.9 | 2.5 | 1.666666667 | 2.76 | 4.6 |
| Penhill Farm | 13.7 | 8.8 | 5.5 | 1.556818182 | 1.6 | 2.490909091 |
| Penhill Farm | 9.5 | 8.2 | 3.5 | 1.158536585 | 2.342857143 | 2.714285714 |
| Penhill Farm | 6.2 | 8 | 3 | 0.775 | 2.666666667 | 2.066666667 |
| Penhill Farm | 11.3 | 7.9 | 4 | 1.430379747 | 1.975 | 2.825 |
| Penhill Farm | 7.5 | 5 | 2.5 | 1.5 | 2 | 3 |
| Penhill Farm | 8.5 | 6 | 2.2 | 1.416666667 | 2.727272727 | 3.863636364 |
| Penhill Farm | 12.1 | 4.7 | 3.6 | 2.574468085 | 1.305555556 | 3.361111111 |
| Penhill Farm | 13 | 7.8 | 4.6 | 1.666666667 | 1.695652174 | 2.826086957 |
| Penhill Farm | 13 | 7.9 | 5 | 1.64556962 | 1.58 | 2.6 |
| Penhill Farm | 7.9 | 5 | 3.1 | 1.58 | 1.612903226 | 2.548387097 |
| Penhill Farm | 11.6 | 7 | 4 | 1.657142857 | 1.75 | 2.9 |
| Penhill Farm | 7.3 | 5 | 3 | 1.46 | 1.666666667 | 2.433333333 |
| Penhill Farm | 8 | 4.9 | 2.4 | 1.632653061 | 2.041666667 | 3.333333333 |
| Penhill Farm | 10.5 | 6 | 4.5 | 1.75 | 1.333333333 | 2.333333333 |
| Penhill Farm | 7.9 | 5 | 2.5 | 1.58 | 2 | 3.16 |
| Penhill Farm | 4.9 | 4 | 2 | 1.225 | 2 | 2.45 |
| Penhill Farm | 6.5 | 5 | 2.2 | 1.3 | 2.272727273 | 2.954545455 |
| Penhill Farm | 13 | 5.9 | 5.7 | 2.203389831 | 1.035087719 | 2.280701754 |
| Penhill Farm | 8.2 | 5.2 | 2.2 | 1.576923077 | 2.363636364 | 3.727272727 |
| Penhill Farm | 11 | 8.7 | 6.5 | 1.264367816 | 1.338461538 | 1.692307692 |
| Namib IV | 15 | 8.6 | 5.2 | 1.744186047 | 1.653846154 | 2.884615385 |
| Namib IV | 13.1 | 7.6 | 4.5 | 1.723684211 | 1.688888889 | 2.911111111 |
| Namib IV | 10.3 | 5.7 | 3.6 | 1.807017544 | 1.583333333 | 2.861111111 |
| Namib IV | 10.9 | 5.7 | 2.7 | 1.912280702 | 2.111111111 | 4.037037037 |
| Namib IV | 10 | 6 | 3.5 | 1.666666667 | 1.714285714 | 2.857142857 |
| Namib IV | 10.3 | 6.1 | 4.4 | 1.68852459 | 1.386363636 | 2.340909091 |
| Namib IV | 12 | 6.9 | 4.2 | 1.739130435 | 1.642857143 | 2.857142857 |
| Namib IV | 10 | 4.7 | 4.8 | 2.127659574 | 0.979166667 | 2.083333333 |
| Namib IV | 7.2 | 6.3 | 2.5 | 1.142857143 | 2.52 | 2.88 |
| Cape Hangklip | 17.203 | 9.71 | 3.94 | 1.771678682 | 2.464467005 | 4.366243655 |
| Cape Hangklip | 14.024 | 8.74 | 3.73 | 1.604576659 | 2.343163539 | 3.759785523 |
| Cape Hangklip | 15.226 | 9.76 | 5.04 | 1.560040984 | 1.936507937 | 3.021031746 |
| Cape Hangklip | 13.689 | 9.35 | 4.26 | 1.464064171 | 2.194835681 | 3.213380282 |
| Cape Hangklip | 19.167 | 15.33 | 7 | 1.250293542 | 2.19 | 2.738142857 |
| Cape Hangklip | 10.805 | 6.41 | 3.41 | 1.685647426 | 1.879765396 | 3.168621701 |
| Cape Hangklip | 14.039 | 7.62 | 4.08 | 1.842388451 | 1.867647059 | 3.440931373 |
| Cape Hangklip | 13.071 | 9.09 | 4.31 | 1.437953795 | 2.109048724 | 3.032714617 |
| Cape Hangklip | 10.925 | 6.09 | 3.42 | 1.793924466 | 1.780701754 | 3.194444444 |
| Cape Hangklip | 14.228 | 9.4 | 4.78 | 1.513617021 | 1.966527197 | 2.976569038 |
| Cape Hangklip | 14.317 | 8.25 | 4.51 | 1.735393939 | 1.829268293 | 3.174501109 |
| Cape Hangklip | 17.027 | 10.65 | 4.85 | 1.598779343 | 2.195876289 | 3.510721649 |
| Cape Hangklip | 10.027 | 7.53 | 3.46 | 1.331606906 | 2.176300578 | 2.897976879 |
| Cape Hangklip | 15.662 | 9.7 | 4.97 | 1.614639175 | 1.951710262 | 3.151307847 |
| Cape Hangklip | 16.481 | 9.97 | 4.26 | 1.653059178 | 2.340375587 | 3.868779343 |
| Cape Hangklip | 14.709 | 8.66 | 4.38 | 1.698498845 | 1.97716895 | 3.358219178 |
| Cape Hangklip | 13.615 | 8.68 | 5.13 | 1.568548387 | 1.692007797 | 2.653996101 |
| Cape Hangklip | 11.985 | 7.08 | 4.11 | 1.69279661 | 1.722627737 | 2.916058394 |
| Cape Hangklip | 16.608 | 11.16 | 4.78 | 1.488172043 | 2.334728033 | 3.474476987 |
| Cape Hangklip | 22.147 | 13.02 | 5.59 | 1.700998464 | 2.329159213 | 3.961896243 |
| Cape Hangklip | 19.474 | 10.53 | 5.54 | 1.849382716 | 1.900722022 | 3.515162455 |
| Cape Hangklip | 25.736 | 10.33 | 7.44 | 2.491384318 | 1.38844086 | 3.459139785 |
| Cape Hangklip | 14.303 | 11.36 | 6.64 | 1.259066901 | 1.710843373 | 2.154066265 |
| Cape Hangklip | 17.956 | 11.52 | 7.77 | 1.558680556 | 1.482625483 | 2.310939511 |
| Cape Hangklip | 21.436 | 13.29 | 6.69 | 1.612942062 | 1.986547085 | 3.204185351 |
| Cape Hangklip | 16.989 | 12.03 | 7.77 | 1.412219451 | 1.548262548 | 2.186486486 |
| Cape Hangklip | 17.592 | 9.26 | 5.04 | 1.899784017 | 1.837301587 | 3.49047619 |
| Cape Hangklip | 17.13 | 9.52 | 5.54 | 1.799369748 | 1.718411552 | 3.092057762 |
| Cape Hangklip | 18.457 | 9.91 | 4.82 | 1.862462159 | 2.056016598 | 3.829253112 |
| Cape Hangklip | 19.095 | 10.74 | 6.83 | 1.777932961 | 1.572474378 | 2.795754026 |
| Cape Hangklip | 14.935 | 8.99 | 4.86 | 1.661290323 | 1.849794239 | 3.073045267 |
| Cape Hangklip | 16.165 | 9.09 | 5.22 | 1.778327833 | 1.74137931 | 3.096743295 |
| Cape Hangklip | 15.224 | 10.05 | 6.16 | 1.514825871 | 1.631493506 | 2.471428571 |
| Cape Hangklip | 25.91 | 15.68 | 9.42 | 1.652423469 | 1.664543524 | 2.750530786 |
| Cape Hangklip | 17.813 | 10.08 | 5.42 | 1.767162698 | 1.859778598 | 3.286531365 |
| Cape Hangklip | 18.226 | 10.48 | 8.39 | 1.739122137 | 1.249106079 | 2.172348033 |
| Cape Hangklip | 15.736 | 9.21 | 4.97 | 1.708577633 | 1.853118712 | 3.166197183 |
| Cape Hangklip | 10.026 | 8.19 | 3.45 | 1.224175824 | 2.373913043 | 2.906086957 |
| Cape Hangklip | 14.619 | 7.89 | 5.61 | 1.852851711 | 1.406417112 | 2.605882353 |
| Cape Hangklip | 17.001 | 13.64 | 6.11 | 1.246407625 | 2.232405892 | 2.782487725 |
| Cape Hangklip | 16.751 | 7.94 | 4.28 | 2.109697733 | 1.855140187 | 3.913785047 |
| Cape Hangklip | 12.292 | 8.21 | 4.18 | 1.497198538 | 1.964114833 | 2.940669856 |
| Cape Hangklip | 18.54 | 10.69 | 7.15 | 1.734331151 | 1.495104895 | 2.593006993 |
| Cape Hangklip | 14.228 | 8.41 | 3.62 | 1.691795482 | 2.32320442 | 3.93038674 |
| Cape Hangklip | 17.424 | 10.72 | 5.21 | 1.625373134 | 2.057581574 | 3.344337812 |
| Cape Hangklip | 11.16 | 7.14 | 3.1 | 1.56302521 | 2.303225806 | 3.6 |
| Cape Hangklip | 29.783 | 13.57 | 8.47 | 2.19476787 | 1.602125148 | 3.516292798 |
| Cape Hangklip | 16.165 | 9.24 | 4.15 | 1.749458874 | 2.226506024 | 3.895180723 |
| Cape Hangklip | 21.121 | 9.54 | 7.33 | 2.2139413 | 1.301500682 | 2.881446112 |
| Cape Hangklip | 14.234 | 8.46 | 5.13 | 1.68250591 | 1.649122807 | 2.774658869 |
| Cape Hangklip | 16.852 | 8.8 | 5.42 | 1.915 | 1.623616236 | 3.109225092 |
| Cape Hangklip | 11.48 | 7.13 | 3.54 | 1.610098177 | 2.014124294 | 3.242937853 |
| Cape Hangklip | 15.519 | 10.22 | 6.22 | 1.518493151 | 1.643086817 | 2.495016077 |
| Cape Hangklip | 17.424 | 9.41 | 6.15 | 1.851647184 | 1.530081301 | 2.833170732 |
| Cape Hangklip | 23.368 | 10.09 | 5.92 | 2.315956392 | 1.704391892 | 3.947297297 |
| Cape Hangklip | 20.129 | 16.23 | 6.87 | 1.240234134 | 2.362445415 | 2.929985444 |
| Cape Hangklip | 21.788 | 12.39 | 7.13 | 1.758514931 | 1.73772791 | 3.055820477 |
| Cape Hangklip | 9.187 | 7.42 | 3.9 | 1.238140162 | 1.902564103 | 2.355641026 |
| Cape Hangklip | 10.227 | 7.05 | 3.72 | 1.450638298 | 1.89516129 | 2.749193548 |
| Cape Hangklip | 11.342 | 6.44 | 3.48 | 1.761180124 | 1.850574713 | 3.259195402 |
| Cape Hangklip | 12 | 7.02 | 3.05 | 1.709401709 | 2.301639344 | 3.93442623 |
| Cape Hangklip | 13.913 | 7.72 | 4.13 | 1.802202073 | 1.869249395 | 3.368765133 |
| Cape Hangklip | 14.397 | 7.96 | 4.96 | 1.808668342 | 1.60483871 | 2.902620968 |
| Cape Hangklip | 12.608 | 6.84 | 4.01 | 1.843274854 | 1.705735661 | 3.144139651 |
| Cape Hangklip | 13.597 | 8.52 | 3.31 | 1.595892019 | 2.574018127 | 4.107854985 |
| Cape Hangklip | 14.208 | 8.86 | 4.22 | 1.603611738 | 2.099526066 | 3.366824645 |
| Cape Hangklip | 14.502 | 9.47 | 4.55 | 1.531362196 | 2.081318681 | 3.187252747 |
| Cape Hangklip | 12.847 | 11.33 | 4.64 | 1.133892321 | 2.441810345 | 2.76875 |
| Cape Hangklip | 13.223 | 7.53 | 3.39 | 1.756042497 | 2.221238938 | 3.900589971 |
| Cape Hangklip | 18.169 | 9.88 | 5.11 | 1.838967611 | 1.933463796 | 3.555577299 |
| Cape Hangklip | 9.069 | 3.95 | 3.26 | 2.295949367 | 1.211656442 | 2.78190184 |
| Cape Hangklip | 12.301 | 5.81 | 5.92 | 2.117211704 | 0.981418919 | 2.077871622 |
| Cape Hangklip | 10.366 | 5.56 | 3.91 | 1.864388489 | 1.421994885 | 2.651150895 |
| Cape Hangklip | 9.882 | 6.15 | 2.82 | 1.606829268 | 2.180851064 | 3.504255319 |
| Cape Hangklip | 9.271 | 6.46 | 2.98 | 1.435139319 | 2.167785235 | 3.111073826 |
| Cape Hangklip | 19.101 | 13.98 | 6.3 | 1.366309013 | 2.219047619 | 3.031904762 |
| Cape Hangklip | 19.581 | 11.58 | 6.72 | 1.690932642 | 1.723214286 | 2.913839286 |
| Cape Hangklip | 17.151 | 10.8 | 6.27 | 1.588055556 | 1.722488038 | 2.735406699 |
| Cape Hangklip | 16.397 | 9.03 | 5.54 | 1.815836102 | 1.629963899 | 2.959747292 |
| Cape Hangklip | 14.131 | 8.09 | 4.13 | 1.746724351 | 1.958837772 | 3.421549637 |
| Cape Hangklip | 11.078 | 8.6 | 3.97 | 1.288139535 | 2.166246851 | 2.790428212 |
| Cape Hangklip | 10.839 | 7.06 | 3.52 | 1.535269122 | 2.005681818 | 3.079261364 |
| Cape Hangklip | 19.534 | 10.75 | 5.99 | 1.817116279 | 1.794657763 | 3.261101836 |
| Cape Hangklip | 14.397 | 10.3 | 7.07 | 1.39776699 | 1.456859972 | 2.036350778 |
| Cape Hangklip | 13.347 | 8.05 | 6.52 | 1.658012422 | 1.234662577 | 2.04708589 |
| Cape Hangklip | 17.807 | 10.75 | 6.13 | 1.656465116 | 1.753670473 | 2.904893964 |
| Cape Hangklip | 22.187 | 12.99 | 5.86 | 1.708006159 | 2.216723549 | 3.786177474 |
| Cape Hangklip | 23.064 | 11.55 | 8.05 | 1.996883117 | 1.434782609 | 2.865093168 |
| Cape Hangklip | 17.871 | 11.31 | 4.66 | 1.580106101 | 2.427038627 | 3.834978541 |
| Cape Hangklip | 18.829 | 10.13 | 6.36 | 1.858736426 | 1.592767296 | 2.960534591 |
| Cape Hangklip | 19.931 | 10.65 | 6.44 | 1.871455399 | 1.653726708 | 3.094875776 |
| Cape Hangklip | 11.614 | 8.85 | 4.8 | 1.312316384 | 1.84375 | 2.419583333 |
| Cape Hangklip | 13.954 | 8.2 | 4.3 | 1.701707317 | 1.906976744 | 3.245116279 |
| Cape Hangklip | 18.273 | 9.72 | 7.49 | 1.879938272 | 1.297730307 | 2.43965287 |
| Cape Hangklip | 21.276 | 14.34 | 7.29 | 1.483682008 | 1.967078189 | 2.918518519 |
| Cape Hangklip | 22.331 | 10.78 | 7.58 | 2.071521336 | 1.422163588 | 2.946042216 |
| Cape Hangklip | 16.709 | 10.12 | 4.74 | 1.651086957 | 2.135021097 | 3.525105485 |
| Cape Hangklip | 15.761 | 9.68 | 5.33 | 1.628202479 | 1.816135084 | 2.957035647 |
| Cape Hangklip | 17.39 | 11.23 | 6.15 | 1.548530721 | 1.82601626 | 2.827642276 |
| Cape Hangklip | 14.967 | 9.56 | 5.75 | 1.565585774 | 1.662608696 | 2.602956522 |
| Cape Hangklip | 20.278 | 10.65 | 4.31 | 1.904037559 | 2.47099768 | 4.70487239 |
| Cape Hangklip | 21.101 | 12.72 | 6.47 | 1.658883648 | 1.965996909 | 3.261360124 |
| Cape Hangklip | 17.25 | 10.92 | 7.33 | 1.57967033 | 1.489768076 | 2.353342428 |
| Cape Hangklip | 16.53 | 9.68 | 3.86 | 1.707644628 | 2.507772021 | 4.28238342 |
| Cape Hangklip | 16.878 | 9.78 | 6.6 | 1.725766871 | 1.481818182 | 2.557272727 |
| Cape Hangklip | 15.348 | 9.28 | 6.02 | 1.65387931 | 1.541528239 | 2.549501661 |
| Cape Hangklip | 16.895 | 9.18 | 4.71 | 1.840413943 | 1.949044586 | 3.587048832 |
| Cape Hangklip | 18.854 | 11.26 | 5.75 | 1.674422735 | 1.95826087 | 3.278956522 |
| Cape Hangklip | 13.546 | 7.43 | 3.64 | 1.823149394 | 2.041208791 | 3.721428571 |
| Cape Hangklip | 22.165 | 11.95 | 6.99 | 1.854811715 | 1.709585122 | 3.170958512 |
| Cape Hangklip | 16.355 | 12.04 | 5.25 | 1.358388704 | 2.293333333 | 3.115238095 |
| Cape Hangklip | 14.173 | 11.02 | 7.66 | 1.286116152 | 1.438642298 | 1.850261097 |
| Cape Hangklip | 14.533 | 8.8 | 3.58 | 1.651477273 | 2.458100559 | 4.059497207 |
| Cape Hangklip | 14.605 | 10.44 | 7.1 | 1.39894636 | 1.470422535 | 2.057042254 |
| Cape Hangklip | 16.208 | 7.99 | 4.4 | 2.02853567 | 1.815909091 | 3.683636364 |
| Cape Hangklip | 11.412 | 7.13 | 4.77 | 1.60056101 | 1.49475891 | 2.39245283 |
| Cape Hangklip | 15.786 | 9.93 | 6.34 | 1.589728097 | 1.566246057 | 2.489905363 |
| Cape Hangklip | 14.965 | 6.67 | 4.71 | 2.243628186 | 1.416135881 | 3.177282378 |
| Cape Hangklip | 23.173 | 12.85 | 6.9 | 1.803346304 | 1.862318841 | 3.358405797 |
| Cape Hangklip | 22.397 | 13.5 | 7.13 | 1.659037037 | 1.893408135 | 3.141234222 |
| Cape Hangklip | 16.91 | 9.52 | 7.96 | 1.776260504 | 1.195979899 | 2.124371859 |
| Cape Hangklip | 16.565 | 9.14 | 4.69 | 1.812363239 | 1.948827292 | 3.531982942 |
| Cape Hangklip | 16.649 | 10.21 | 5.79 | 1.630656219 | 1.763385147 | 2.875474957 |
| Cape Hangklip | 16.269 | 9.11 | 5.13 | 1.785839737 | 1.77582846 | 3.171345029 |
| Cape Hangklip | 17.474 | 10.3 | 5.42 | 1.696504854 | 1.900369004 | 3.22398524 |
| Cape Hangklip | 15.043 | 8.58 | 4.09 | 1.753263403 | 2.097799511 | 3.67799511 |
| Cape Hangklip | 12.482 | 6.99 | 3.39 | 1.785693848 | 2.061946903 | 3.6820059 |
| Cape Hangklip | 27.294 | 15.89 | 6.59 | 1.717684078 | 2.411229135 | 4.141729894 |
| Cape Hangklip | 13.533 | 7.57 | 3.56 | 1.787714663 | 2.126404494 | 3.801404494 |
| Cape Hangklip | 11.276 | 6.18 | 4.01 | 1.824595469 | 1.541147132 | 2.811970075 |
| Cape Hangklip | 11.328 | 7.37 | 3.7 | 1.537042062 | 1.991891892 | 3.061621622 |
| Cape Hangklip | 15.977 | 12.01 | 4.35 | 1.330308077 | 2.76091954 | 3.672873563 |
| Cape Hangklip | 11.564 | 7.11 | 3.13 | 1.626441632 | 2.271565495 | 3.69456869 |
| Cape Hangklip | 10.232 | 6.81 | 3.02 | 1.502496329 | 2.254966887 | 3.38807947 |
| Cape Hangklip | 11.079 | 8.22 | 4.25 | 1.347810219 | 1.934117647 | 2.606823529 |
| Cape Hangklip | 15.183 | 8.21 | 5.71 | 1.849330085 | 1.437828371 | 2.659019264 |
| Cape Hangklip | 17.971 | 11.56 | 5.18 | 1.554584775 | 2.231660232 | 3.469305019 |
| Cape Hangklip | 24.931 | 12.68 | 8.49 | 1.966167192 | 1.49352179 | 2.936513545 |
| Cape Hangklip | 17.133 | 8.55 | 4.92 | 2.003859649 | 1.737804878 | 3.482317073 |
| Cape Hangklip | 13.808 | 9.96 | 4.16 | 1.386345382 | 2.394230769 | 3.319230769 |
| Cape Hangklip | 14.213 | 8.48 | 4.55 | 1.676061321 | 1.863736264 | 3.123736264 |
| Cape Hangklip | 15.3 | 9.74 | 6.81 | 1.570841889 | 1.430249633 | 2.246696035 |
| Cape Hangklip | 17.498 | 9.53 | 6.88 | 1.836096537 | 1.385174419 | 2.543313953 |
| Cape Hangklip | 9.986 | 6.1 | 3.78 | 1.63704918 | 1.613756614 | 2.641798942 |
| Cape Hangklip | 11.402 | 7.41 | 2.91 | 1.538731444 | 2.546391753 | 3.918213058 |
| Cape Hangklip | 10.923 | 6.49 | 3.78 | 1.683050847 | 1.716931217 | 2.88968254 |
| Cape Hangklip | 9.726 | 7.69 | 3.61 | 1.264759428 | 2.130193906 | 2.694182825 |
| Cape Hangklip | 12.128 | 7.29 | 3.39 | 1.663648834 | 2.150442478 | 3.577581121 |
| Cape Hangklip | 10.141 | 7 | 2.54 | 1.448714286 | 2.755905512 | 3.992519685 |
| Cape Hangklip | 8.165 | 5.13 | 2.6 | 1.591617934 | 1.973076923 | 3.140384615 |
| Cape Hangklip | 8.914 | 5.79 | 3.52 | 1.53955095 | 1.644886364 | 2.532386364 |
| Cape Hangklip | 11.765 | 6.72 | 3.24 | 1.750744048 | 2.074074074 | 3.63117284 |
| Cape Hangklip | 11.385 | 6.15 | 3.42 | 1.851219512 | 1.798245614 | 3.328947368 |
| Cape Hangklip | 10.642 | 6.79 | 4.05 | 1.56730486 | 1.67654321 | 2.627654321 |
| Cape Hangklip | 10.746 | 5.65 | 3.91 | 1.901946903 | 1.445012788 | 2.748337596 |
| Cape Hangklip | 8.676 | 5.51 | 2.69 | 1.574591652 | 2.048327138 | 3.22527881 |
| Cape Hangklip | 15.724 | 9.11 | 4.2 | 1.726015368 | 2.169047619 | 3.743809524 |
| Cape Hangklip | 18.515 | 9.6 | 4.29 | 1.928645833 | 2.237762238 | 4.315850816 |
| Cape Hangklip | 17.415 | 11.16 | 5.34 | 1.560483871 | 2.08988764 | 3.261235955 |
| Cape Hangklip | 21.273 | 10.58 | 6.49 | 2.010680529 | 1.630200308 | 3.277812018 |
| Cape Hangklip | 19.056 | 10.32 | 5.4 | 1.846511628 | 1.911111111 | 3.528888889 |
| Cape Hangklip | 10.624 | 9.43 | 6.66 | 1.126617179 | 1.415915916 | 1.595195195 |
| Cape Hangklip | 13.934 | 10.52 | 4.34 | 1.324524715 | 2.423963134 | 3.210599078 |
| Cape Hangklip | 11.817 | 6.3 | 3.63 | 1.875714286 | 1.73553719 | 3.255371901 |
| Cape Hangklip | 15.348 | 8.48 | 6.08 | 1.80990566 | 1.394736842 | 2.524342105 |
| Cape Hangklip | 15.983 | 10.15 | 6.56 | 1.574679803 | 1.547256098 | 2.436432927 |
| Cape Hangklip | 17.062 | 9.57 | 4.81 | 1.782863114 | 1.98960499 | 3.547193347 |
| Cape Hangklip | 19.157 | 11.99 | 4.95 | 1.597748123 | 2.422222222 | 3.87010101 |
| Cape Hangklip | 16.91 | 9.86 | 6.03 | 1.715010142 | 1.635157546 | 2.804311774 |
| Cape Hangklip | 16.753 | 10.39 | 7.05 | 1.612415784 | 1.473758865 | 2.376312057 |
| Cape Hangklip | 18.911 | 9.57 | 5.99 | 1.976071055 | 1.597662771 | 3.157095159 |
| Cape Hangklip | 18.069 | 11 | 5.47 | 1.642636364 | 2.010968921 | 3.303290676 |
| Cape Hangklip | 18.916 | 13.55 | 5.7 | 1.39601476 | 2.377192982 | 3.318596491 |
| Cape Hangklip | 18.51 | 13.37 | 6.72 | 1.384442782 | 1.989583333 | 2.754464286 |
| Cape Hangklip | 19.583 | 11.55 | 5.19 | 1.695497835 | 2.225433526 | 3.773217726 |
| Cape Hangklip | 21.761 | 12.21 | 6.12 | 1.782227682 | 1.995098039 | 3.555718954 |
| Cape Hangklip | 17.623 | 11.07 | 4.82 | 1.591960253 | 2.296680498 | 3.656224066 |
| Cape Hangklip | 18.804 | 11.89 | 5.09 | 1.581497056 | 2.335952849 | 3.694302554 |
| Cape Hangklip | 15.934 | 9.28 | 4.3 | 1.717025862 | 2.158139535 | 3.705581395 |
| Cape Hangklip | 18.69 | 11.64 | 5.86 | 1.605670103 | 1.986348123 | 3.189419795 |
| Cape Hangklip | 17.92 | 10.73 | 4.26 | 1.670083877 | 2.518779343 | 4.20657277 |
| Cape Hangklip | 16.319 | 11.16 | 5.53 | 1.462275986 | 2.018083183 | 2.950994575 |
| Cape Hangklip | 15.823 | 7.16 | 3.99 | 2.209916201 | 1.794486216 | 3.96566416 |
| Cape Hangklip | 16.784 | 10.37 | 5.34 | 1.618514947 | 1.941947566 | 3.143071161 |
| Cape Hangklip | 19.037 | 10.08 | 6.81 | 1.88859127 | 1.480176211 | 2.795447871 |
| Cape Hangklip | 17.592 | 10.45 | 5.14 | 1.683444976 | 2.03307393 | 3.422568093 |
| Cape Hangklip | 15.977 | 11.69 | 6.25 | 1.366723695 | 1.8704 | 2.55632 |
| Cape Hangklip | 17.87 | 10.9 | 6.08 | 1.639449541 | 1.792763158 | 2.939144737 |
| Cape Hangklip | 14.168 | 9.06 | 4.96 | 1.563796909 | 1.826612903 | 2.856451613 |
| Cape Hangklip | 20.722 | 11.61 | 6.5 | 1.784840655 | 1.786153846 | 3.188 |
| Cape Hangklip | 20.686 | 17.23 | 5.85 | 1.200580383 | 2.945299145 | 3.536068376 |
| Cape Hangklip | 13.987 | 8.13 | 4.78 | 1.720418204 | 1.70083682 | 2.926150628 |
| Cape Hangklip | 11.26 | 7.11 | 3.61 | 1.583684951 | 1.969529086 | 3.119113573 |
| Cape Hangklip | 12.608 | 7.17 | 3.9 | 1.758437936 | 1.838461538 | 3.232820513 |
| Cape Hangklip | 10.884 | 7.55 | 3.39 | 1.441589404 | 2.227138643 | 3.210619469 |
| Cape Hangklip | 17.97 | 10.55 | 5.65 | 1.703317536 | 1.867256637 | 3.180530973 |
| Cape Hangklip | 15.662 | 9.46 | 3.86 | 1.655602537 | 2.450777202 | 4.057512953 |
| Cape Hangklip | 10.906 | 6.41 | 3.07 | 1.701404056 | 2.087947883 | 3.552442997 |
| Cape Hangklip | 15.513 | 8.42 | 4.59 | 1.84239905 | 1.834422658 | 3.379738562 |
| Cape Hangklip | 12.059 | 6.86 | 3.79 | 1.75787172 | 1.810026385 | 3.181794195 |
| Cape Hangklip | 15.86 | 11.19 | 4.64 | 1.417336908 | 2.411637931 | 3.418103448 |
| Cape Hangklip | 15.451 | 9.01 | 4.18 | 1.714872364 | 2.155502392 | 3.696411483 |
| Cape Hangklip | 12.608 | 7.73 | 4.45 | 1.631047865 | 1.737078652 | 2.833258427 |
| Cape Hangklip | 13.76 | 9.28 | 4.22 | 1.482758621 | 2.199052133 | 3.260663507 |
| Cape Hangklip | 14.955 | 9.6 | 4.92 | 1.5578125 | 1.951219512 | 3.039634146 |
| Cape Hangklip | 19.104 | 12.56 | 8.78 | 1.521019108 | 1.430523918 | 2.175854214 |
| Cape Hangklip | 17.424 | 9.08 | 4.74 | 1.918942731 | 1.915611814 | 3.675949367 |
| Cape Hangklip | 26.775 | 11.82 | 6.54 | 2.265228426 | 1.80733945 | 4.094036697 |
| Cape Hangklip | 15.091 | 9.73 | 5.49 | 1.550976362 | 1.772313297 | 2.748816029 |
| Cape Hangklip | 18.442 | 9.43 | 8.04 | 1.955673383 | 1.172885572 | 2.293781095 |
| Cape Hangklip | 11.918 | 7.38 | 2.64 | 1.614905149 | 2.795454545 | 4.514393939 |
| Cape Hangklip | 10.704 | 6.69 | 4.16 | 1.6 | 1.608173077 | 2.573076923 |
| Cape Hangklip | 9.174 | 5.96 | 3.19 | 1.539261745 | 1.868338558 | 2.875862069 |
| Cape Hangklip | 10.331 | 5.98 | 2.31 | 1.727591973 | 2.588744589 | 4.472294372 |
| Cape Hangklip | 13.696 | 8.97 | 3.49 | 1.526867336 | 2.570200573 | 3.924355301 |
| Cape Hangklip | 10.746 | 7.84 | 2.82 | 1.370663265 | 2.780141844 | 3.810638298 |
| Cape Hangklip | 9.086 | 7.15 | 3.29 | 1.270769231 | 2.17325228 | 2.761702128 |
| Cape Hangklip | 9.381 | 5.89 | 3.46 | 1.592699491 | 1.702312139 | 2.711271676 |
| Cape Hangklip | 16.759 | 9.31 | 5.25 | 1.800107411 | 1.773333333 | 3.192190476 |
| Cape Hangklip | 17.176 | 11.74 | 5.75 | 1.463032368 | 2.04173913 | 2.987130435 |
| Cape Hangklip | 16.122 | 7.49 | 4.62 | 2.15246996 | 1.621212121 | 3.48961039 |
| Cape Hangklip | 9.796 | 6.12 | 3.22 | 1.600653595 | 1.900621118 | 3.042236025 |
| Cape Hangklip | 11.26 | 6.96 | 3.81 | 1.617816092 | 1.826771654 | 2.955380577 |
| Cape Hangklip | 17.314 | 9.69 | 4.51 | 1.786790506 | 2.148558758 | 3.83902439 |
| Cape Hangklip | 12.683 | 8.19 | 4.09 | 1.548595849 | 2.002444988 | 3.100977995 |
| Cape Hangklip | 10.536 | 5.14 | 3.06 | 2.049805447 | 1.679738562 | 3.443137255 |
| Cape Hangklip | 9.156 | 5.99 | 3 | 1.528547579 | 1.996666667 | 3.052 |
| Cape Hangklip | 12.836 | 6.7 | 3.2 | 1.915820896 | 2.09375 | 4.01125 |
| Cape Hangklip | 16.73 | 11.03 | 4.82 | 1.516772439 | 2.288381743 | 3.470954357 |
| Cape Hangklip | 22.716 | 10.69 | 4.3 | 2.124976614 | 2.486046512 | 5.282790698 |
| Cape Hangklip | 21.373 | 10.63 | 5.13 | 2.010630292 | 2.072124756 | 4.166276803 |
| Cape Hangklip | 12.945 | 7.03 | 3.37 | 1.841394026 | 2.086053412 | 3.841246291 |
| Cape Hangklip | 12.708 | 8.38 | 4.11 | 1.51646778 | 2.03892944 | 3.091970803 |
| Cape Hangklip | 15.65 | 10.09 | 4.24 | 1.551040634 | 2.379716981 | 3.691037736 |
| Cape Hangklip | 14.629 | 7.91 | 4.49 | 1.8494311 | 1.76169265 | 3.258129176 |
| Cape Hangklip | 12.903 | 8.29 | 3.49 | 1.556453559 | 2.375358166 | 3.69713467 |
| Cape Hangklip | 18.739 | 10.6 | 4.9 | 1.767830189 | 2.163265306 | 3.824285714 |
| Cape Hangklip | 13.85 | 8.84 | 4.16 | 1.566742081 | 2.125 | 3.329326923 |
| Cape Hangklip | 17.693 | 10.6 | 6.2 | 1.669150943 | 1.709677419 | 2.853709677 |
| Cape Hangklip | 18.374 | 10.65 | 5.34 | 1.725258216 | 1.994382022 | 3.44082397 |
| Cape Hangklip | 18.938 | 11.02 | 5.04 | 1.718511797 | 2.186507937 | 3.757539683 |
| Cape Hangklip | 19.864 | 10.17 | 4.67 | 1.953195674 | 2.177730193 | 4.253533191 |
| Cape Hangklip | 16.332 | 9.85 | 5.47 | 1.658071066 | 1.800731261 | 2.985740402 |
| Cape Hangklip | 11.112 | 6.15 | 4.85 | 1.806829268 | 1.268041237 | 2.291134021 |
| Cape Hangklip | 16.153 | 10.55 | 4.64 | 1.531090047 | 2.273706897 | 3.48125 |
| Cape Hangklip | 17.623 | 10.25 | 6.22 | 1.719317073 | 1.647909968 | 2.833279743 |
| Cape Hangklip | 11.901 | 7.74 | 4.4 | 1.537596899 | 1.759090909 | 2.704772727 |
| Cape Hangklip | 14.102 | 9.47 | 4.42 | 1.489123548 | 2.142533937 | 3.190497738 |
| Cape Hangklip | 12.522 | 8.02 | 4.92 | 1.561346633 | 1.630081301 | 2.545121951 |
| Cape Hangklip | 13.718 | 8.31 | 4.93 | 1.65078219 | 1.685598377 | 2.782555781 |
| Cape Hangklip | 13.008 | 7.72 | 4.79 | 1.684974093 | 1.611691023 | 2.71565762 |
| Cape Hangklip | 13.409 | 8.58 | 5.48 | 1.562820513 | 1.565693431 | 2.44689781 |
| Cape Hangklip | 16.28 | 11.18 | 4.59 | 1.456171735 | 2.435729847 | 3.546840959 |
| Cape Hangklip | 12.103 | 9.81 | 4.2 | 1.233741081 | 2.335714286 | 2.881666667 |
| Cape Hangklip | 14.576 | 7.54 | 3.62 | 1.933156499 | 2.082872928 | 4.026519337 |
| Cape Hangklip | 14.222 | 9.48 | 5.51 | 1.50021097 | 1.720508167 | 2.581125227 |
| Cape Hangklip | 15.548 | 11.84 | 5.18 | 1.313175676 | 2.285714286 | 3.001544402 |
| Cape Hangklip | 18.627 | 11.03 | 4.34 | 1.688757933 | 2.541474654 | 4.291935484 |
| Cape Hangklip | 20.484 | 10.48 | 4.93 | 1.954580153 | 2.125760649 | 4.154969574 |
| Cape Hangklip | 24.457 | 14.62 | 8.15 | 1.672845417 | 1.793865031 | 3.000858896 |
| Cape Hangklip | 15.028 | 9.66 | 5.64 | 1.555693582 | 1.712765957 | 2.664539007 |
| Cape Hangklip | 8.883 | 6.18 | 2.73 | 1.437378641 | 2.263736264 | 3.253846154 |
| Cape Hangklip | 8.794 | 5.25 | 2.85 | 1.675047619 | 1.842105263 | 3.085614035 |
| Cape Hangklip | 11.644 | 7.03 | 3.51 | 1.656330014 | 2.002849003 | 3.317378917 |
| Cape Hangklip | 10.106 | 5.74 | 2.11 | 1.760627178 | 2.720379147 | 4.78957346 |
| Cape Hangklip | 9.17 | 5.5 | 2.88 | 1.667272727 | 1.909722222 | 3.184027778 |
| Cape Hangklip | 10.051 | 5.13 | 2.18 | 1.959259259 | 2.353211009 | 4.610550459 |
| Cape Hangklip | 7.429 | 5.04 | 3.75 | 1.474007937 | 1.344 | 1.981066667 |
| Elandsfontein | 13.615 | 9.57 | 4.61 | 1.422675026 | 2.075921909 | 2.953362256 |
| Elandsfontein | 13.576 | 9.98 | 3.87 | 1.360320641 | 2.57881137 | 3.508010336 |
| Elandsfontein | 14.208 | 9.32 | 3.74 | 1.524463519 | 2.49197861 | 3.798930481 |
| Elandsfontein | 14.255 | 8.33 | 4.26 | 1.711284514 | 1.955399061 | 3.346244131 |
| Elandsfontein | 12.713 | 8.36 | 4.15 | 1.52069378 | 2.014457831 | 3.063373494 |
| Elandsfontein | 14.23 | 9.24 | 4.77 | 1.54004329 | 1.937106918 | 2.983228512 |
| Elandsfontein | 17.573 | 9.66 | 4.92 | 1.819151139 | 1.963414634 | 3.571747967 |
| Elandsfontein | 16.397 | 9.18 | 3.84 | 1.786165577 | 2.390625 | 4.270052083 |
| Elandsfontein | 14.894 | 9.37 | 5.13 | 1.589541089 | 1.826510721 | 2.90331384 |
| Elandsfontein | 15.66 | 8.95 | 4.26 | 1.74972067 | 2.100938967 | 3.676056338 |
| Elandsfontein | 15.281 | 9.37 | 3.82 | 1.630843116 | 2.452879581 | 4.00026178 |
| Elandsfontein | 15.802 | 9.8 | 4.82 | 1.61244898 | 2.033195021 | 3.278423237 |
| Elandsfontein | 10.931 | 7.48 | 3.72 | 1.461363636 | 2.010752688 | 2.93844086 |
| Elandsfontein | 12.089 | 9.43 | 3.82 | 1.281972428 | 2.468586387 | 3.164659686 |
| Elandsfontein | 11.716 | 8.13 | 4.65 | 1.441082411 | 1.748387097 | 2.519569892 |
| Elandsfontein | 10.816 | 7.43 | 3.72 | 1.455720054 | 1.997311828 | 2.907526882 |
| Elandsfontein | 12.584 | 8.87 | 3.27 | 1.418714769 | 2.712538226 | 3.848318043 |
| Elandsfontein | 14.755 | 9.79 | 5.23 | 1.507150153 | 1.871892925 | 2.821223709 |
| Elandsfontein | 17.415 | 10.68 | 4.41 | 1.630617978 | 2.421768707 | 3.948979592 |
| Elandsfontein | 16.483 | 8.93 | 5.15 | 1.845800672 | 1.733980583 | 3.200582524 |
| Elandsfontein | 10.988 | 6.37 | 2.78 | 1.724960754 | 2.291366906 | 3.952517986 |
| Elandsfontein | 8.942 | 7.79 | 4.27 | 1.1478819 | 1.824355972 | 2.094145199 |
| Elandsfontein | 13.2 | 6.85 | 2.95 | 1.927007299 | 2.322033898 | 4.474576271 |
| Elandsfontein | 10.026 | 9.24 | 4.03 | 1.085064935 | 2.29280397 | 2.487841191 |
| Elandsfontein | 10.198 | 6.56 | 3.49 | 1.554573171 | 1.87965616 | 2.922063037 |
| Elandsfontein | 9.339 | 7.55 | 3.67 | 1.236953642 | 2.057220708 | 2.544686649 |
| Elandsfontein | 11.008 | 6.46 | 3.21 | 1.704024768 | 2.012461059 | 3.429283489 |
| Elandsfontein | 9.474 | 6.54 | 2.95 | 1.448623853 | 2.216949153 | 3.211525424 |
| Elandsfontein | 7.777 | 6.23 | 2.73 | 1.248314607 | 2.282051282 | 2.848717949 |
| Elandsfontein | 8.951 | 6.81 | 3.13 | 1.314390602 | 2.17571885 | 2.859744409 |
| Elandsfontein | 8.648 | 6.15 | 3.2 | 1.406178862 | 1.921875 | 2.7025 |
| Elandsfontein | 8.112 | 5.77 | 2.79 | 1.405892548 | 2.068100358 | 2.907526882 |
| Elandsfontein | 6.637 | 5.13 | 1.81 | 1.293762183 | 2.834254144 | 3.666850829 |
| Elandsfontein | 7.359 | 5.74 | 1.82 | 1.282055749 | 3.153846154 | 4.043406593 |
| Elandsfontein | 7.511 | 5.16 | 2.68 | 1.455620155 | 1.925373134 | 2.80261194 |
| Elandsfontein | 7.419 | 5.25 | 2.57 | 1.413142857 | 2.042801556 | 2.886770428 |
| Elandsfontein | 10.335 | 7.8 | 4.07 | 1.325 | 1.916461916 | 2.539312039 |
| Elandsfontein | 13.12 | 8 | 2.11 | 1.64 | 3.791469194 | 6.218009479 |
| Elandsfontein | 13.43 | 8.68 | 4.11 | 1.547235023 | 2.111922141 | 3.267639903 |
| Elandsfontein | 15.997 | 9.09 | 3.93 | 1.759845985 | 2.312977099 | 4.070483461 |
| Elandsfontein | 14.193 | 9.18 | 4.86 | 1.546078431 | 1.888888889 | 2.92037037 |
| Elandsfontein | 16.297 | 8.64 | 5.99 | 1.886226852 | 1.442404007 | 2.720701169 |
| Elandsfontein | 23.932 | 9.81 | 5.17 | 2.439551478 | 1.897485493 | 4.62901354 |
| Elandsfontein | 19.643 | 10.57 | 6.86 | 1.858372753 | 1.540816327 | 2.863411079 |
| Elandsfontein | 20.229 | 11.44 | 5.76 | 1.768269231 | 1.986111111 | 3.511979167 |
| Elandsfontein | 19.459 | 11.74 | 6.04 | 1.657495741 | 1.943708609 | 3.221688742 |
| Elandsfontein | 12.558 | 7.15 | 4.43 | 1.756363636 | 1.613995485 | 2.83476298 |
| Elandsfontein | 11.572 | 7.91 | 4.81 | 1.462958281 | 1.644490644 | 2.405821206 |
| Elandsfontein | 15.323 | 8.42 | 3.55 | 1.819833729 | 2.371830986 | 4.316338028 |
| Elandsfontein | 11.648 | 6.39 | 3.01 | 1.8228482 | 2.122923588 | 3.869767442 |
| Elandsfontein | 13.654 | 7.72 | 5.05 | 1.76865285 | 1.528712871 | 2.703762376 |
| Elandsfontein | 14.208 | 9.64 | 5.02 | 1.473858921 | 1.920318725 | 2.830278884 |
| Elandsfontein | 13.002 | 8.25 | 4.43 | 1.576 | 1.862302483 | 2.934988713 |
| Elandsfontein | 13.87 | 7.36 | 5.4 | 1.88451087 | 1.362962963 | 2.568518519 |
| Elandsfontein | 12.312 | 7.84 | 5.09 | 1.570408163 | 1.540275049 | 2.418860511 |
| Elandsfontein | 17.592 | 8.91 | 3.61 | 1.974410774 | 2.468144044 | 4.873130194 |
| Elandsfontein | 15.314 | 10.23 | 4.45 | 1.496969697 | 2.298876404 | 3.441348315 |
| Elandsfontein | 17.036 | 9.59 | 5.54 | 1.776433785 | 1.731046931 | 3.075090253 |
| Elandsfontein | 19.722 | 10.49 | 5.2 | 1.880076263 | 2.017307692 | 3.792692308 |
| Elandsfontein | 18.02 | 10.52 | 5.37 | 1.712927757 | 1.959031657 | 3.355679702 |
| Elandsfontein | 16.133 | 10.13 | 4.92 | 1.592596249 | 2.058943089 | 3.279065041 |
| Elandsfontein | 15.781 | 6.93 | 4 | 2.277200577 | 1.7325 | 3.94525 |
| Elandsfontein | 14.77 | 8.13 | 3.64 | 1.816728167 | 2.233516484 | 4.057692308 |
| Elandsfontein | 12.295 | 8.07 | 3.89 | 1.52354399 | 2.074550129 | 3.16066838 |
| Elandsfontein | 12.953 | 7.18 | 4.45 | 1.804038997 | 1.613483146 | 2.910786517 |
| Elandsfontein | 12.853 | 6.69 | 3.99 | 1.92122571 | 1.676691729 | 3.221303258 |
| Elandsfontein | 12.845 | 7.74 | 3.63 | 1.659560724 | 2.132231405 | 3.538567493 |
| Elandsfontein | 12.339 | 7.1 | 3.02 | 1.737887324 | 2.350993377 | 4.085761589 |
| Elandsfontein | 9.794 | 6.19 | 3.05 | 1.582229402 | 2.029508197 | 3.211147541 |
| Elandsfontein | 10.59 | 7.43 | 3.56 | 1.425302826 | 2.087078652 | 2.974719101 |
| Elandsfontein | 12.334 | 8.17 | 4.53 | 1.509669523 | 1.803532009 | 2.722737307 |
| Elandsfontein | 19.258 | 8.96 | 5.82 | 2.149330357 | 1.5395189 | 3.308934708 |
| Elandsfontein | 16.67 | 8.84 | 5.31 | 1.885746606 | 1.664783427 | 3.139359699 |
| Elandsfontein | 16.544 | 7.91 | 6.08 | 2.091529709 | 1.300986842 | 2.721052632 |
| Elandsfontein | 9.63 | 4.63 | 2.11 | 2.079913607 | 2.194312796 | 4.563981043 |
| Elandsfontein | 15.44 | 7.94 | 5.88 | 1.944584383 | 1.350340136 | 2.62585034 |
| Elandsfontein | 19.132 | 10.42 | 5.6 | 1.836084453 | 1.860714286 | 3.416428571 |
| Elandsfontein | 14.373 | 9.52 | 5.44 | 1.509768908 | 1.75 | 2.642095588 |
| Elandsfontein | 15.08 | 8.15 | 4.93 | 1.850306748 | 1.653144016 | 3.058823529 |
| Elandsfontein | 16.029 | 9.61 | 4.9 | 1.667950052 | 1.96122449 | 3.27122449 |
| Elandsfontein | 15.339 | 8.47 | 6.12 | 1.810979929 | 1.383986928 | 2.506372549 |
| Elandsfontein | 12.204 | 7.08 | 4.39 | 1.723728814 | 1.612756264 | 2.779954442 |
| Elandsfontein | 10.451 | 7.5 | 2.99 | 1.393466667 | 2.508361204 | 3.495317726 |
| Elandsfontein | 11.035 | 7.58 | 6.06 | 1.455804749 | 1.250825083 | 1.820957096 |
| Elandsfontein | 15.134 | 7.6 | 5.77 | 1.991315789 | 1.317157712 | 2.62287695 |
| Elandsfontein | 10.686 | 7.8 | 4.55 | 1.37 | 1.714285714 | 2.348571429 |
| Elandsfontein | 10.487 | 7.36 | 4.37 | 1.42486413 | 1.684210526 | 2.399771167 |
| Elandsfontein | 14.337 | 7.9 | 5.21 | 1.814810127 | 1.516314779 | 2.751823417 |
| Elandsfontein | 12.053 | 6.22 | 3.2 | 1.93778135 | 1.94375 | 3.7665625 |
| Elandsfontein | 13.019 | 7.31 | 5.21 | 1.780984952 | 1.403071017 | 2.498848369 |
| Elandsfontein | 10.093 | 6.81 | 4.24 | 1.482085169 | 1.606132075 | 2.380424528 |
| Elandsfontein | 9.406 | 7.37 | 4.65 | 1.276255088 | 1.584946237 | 2.022795699 |
| Elandsfontein | 10.906 | 6.15 | 3.42 | 1.773333333 | 1.798245614 | 3.188888889 |
| Elandsfontein | 10.782 | 5.57 | 2.82 | 1.93572711 | 1.975177305 | 3.823404255 |
| Elandsfontein | 10.232 | 7.28 | 4.02 | 1.405494505 | 1.810945274 | 2.545273632 |
| Elandsfontein | 11.885 | 7.59 | 4.37 | 1.565876153 | 1.736842105 | 2.719679634 |
| Elandsfontein | 12.592 | 6.03 | 4.89 | 2.088225539 | 1.233128834 | 2.575051125 |
| Elandsfontein | 17.323 | 8.69 | 4.28 | 1.993440736 | 2.030373832 | 4.047429907 |
| Elandsfontein | 18.32 | 11.11 | 4.88 | 1.648964896 | 2.276639344 | 3.754098361 |
| Elandsfontein | 18.597 | 12.79 | 5.08 | 1.454026583 | 2.517716535 | 3.660826772 |
| Elandsfontein | 16.153 | 9.32 | 5.39 | 1.733154506 | 1.729128015 | 2.996846011 |
| Elandsfontein | 15.176 | 10.21 | 5.21 | 1.486385896 | 1.959692898 | 2.912859885 |
| Elandsfontein | 14.165 | 8.95 | 4.71 | 1.582681564 | 1.900212314 | 3.007430998 |
| Elandsfontein | 14.46 | 9.18 | 4.57 | 1.575163399 | 2.008752735 | 3.164113786 |
| Elandsfontein | 15.811 | 10.58 | 6.91 | 1.49442344 | 1.531114327 | 2.28813314 |
| Elandsfontein | 16.002 | 10.15 | 5.04 | 1.576551724 | 2.013888889 | 3.175 |
| Elandsfontein | 16.117 | 9.85 | 6.6 | 1.636243655 | 1.492424242 | 2.441969697 |
| Elandsfontein | 18.144 | 11.14 | 5.37 | 1.628725314 | 2.074487896 | 3.37877095 |
| Elandsfontein | 9.001 | 5.77 | 3.33 | 1.559965338 | 1.732732733 | 2.703003003 |
| Elandsfontein | 9.364 | 6.7 | 3.35 | 1.39761194 | 2 | 2.795223881 |
| Elandsfontein | 9.17 | 5.71 | 3.06 | 1.605954466 | 1.866013072 | 2.996732026 |
| Elandsfontein | 10.245 | 5.53 | 2.77 | 1.852622061 | 1.996389892 | 3.698555957 |
| Elandsfontein | 10.659 | 6.36 | 3.38 | 1.675943396 | 1.881656805 | 3.153550296 |
| Elandsfontein | 8.57 | 5.09 | 2.99 | 1.683693517 | 1.702341137 | 2.866220736 |
| Elandsfontein | 8.086 | 5.72 | 2.95 | 1.413636364 | 1.938983051 | 2.741016949 |
| Elandsfontein | 7.785 | 6.1 | 2.34 | 1.276229508 | 2.606837607 | 3.326923077 |
| Elandsfontein | 7.207 | 5.14 | 1.71 | 1.402140078 | 3.005847953 | 4.214619883 |
| Elandsfontein | 6.228 | 4.59 | 2.46 | 1.356862745 | 1.865853659 | 2.531707317 |
| Elandsfontein | 8.073 | 4.2 | 3.22 | 1.922142857 | 1.304347826 | 2.507142857 |
| Elandsfontein | 15.534 | 7.8 | 4.22 | 1.991538462 | 1.848341232 | 3.681042654 |
| Elandsfontein | 9.513 | 5.12 | 2.93 | 1.858007813 | 1.747440273 | 3.246757679 |
| Elandsfontein | 15.447 | 10.32 | 4.85 | 1.496802326 | 2.127835052 | 3.184948454 |
| Elandsfontein | 14.755 | 9.2 | 5.17 | 1.603804348 | 1.779497099 | 2.853965184 |
| Elandsfontein | 13.599 | 7.96 | 5.8 | 1.708417085 | 1.372413793 | 2.344655172 |
| Elandsfontein | 12.065 | 8.31 | 5.97 | 1.451865223 | 1.391959799 | 2.020938023 |
| Elandsfontein | 11.738 | 8.27 | 4.53 | 1.419347037 | 1.825607064 | 2.591169978 |
| Elandsfontein | 10.552 | 6.57 | 3.07 | 1.60608828 | 2.140065147 | 3.43713355 |
| Elandsfontein | 17.92 | 10.98 | 5.28 | 1.632058288 | 2.079545455 | 3.393939394 |
| Elandsfontein | 16.357 | 10.62 | 5.33 | 1.540207156 | 1.99249531 | 3.068855535 |
| Elandsfontein | 15.497 | 9.49 | 3.49 | 1.632982086 | 2.719197708 | 4.440401146 |
| Elandsfontein | 15.116 | 10.28 | 4.57 | 1.470428016 | 2.249452954 | 3.307658643 |
| Elandsfontein | 14.297 | 8.91 | 5.02 | 1.604601571 | 1.774900398 | 2.848007968 |
| Elandsfontein | 14.692 | 8.9 | 5.23 | 1.650786517 | 1.701720841 | 2.80917782 |
| Elandsfontein | 13.387 | 7.3 | 5.79 | 1.833835616 | 1.260794473 | 2.31208981 |
| Elandsfontein | 12.39 | 7.47 | 4.33 | 1.658634538 | 1.72517321 | 2.861431871 |
| Elandsfontein | 12.879 | 6.81 | 3.45 | 1.891189427 | 1.973913043 | 3.733043478 |
| Elandsfontein | 11.817 | 7.08 | 3.75 | 1.669067797 | 1.888 | 3.1512 |
| Elandsfontein | 10.215 | 8.06 | 4.28 | 1.267369727 | 1.88317757 | 2.386682243 |
| Elandsfontein | 10.131 | 6.73 | 3.48 | 1.505349183 | 1.933908046 | 2.911206897 |
| Elandsfontein | 10.384 | 7.6 | 2.87 | 1.366315789 | 2.648083624 | 3.618118467 |
| Elandsfontein | 20.279 | 10.01 | 7.11 | 2.025874126 | 1.407876231 | 2.852180028 |
| Elandsfontein | 18.438 | 10.52 | 7.43 | 1.752661597 | 1.415881561 | 2.481561238 |
| Elandsfontein | 9.433 | 5.99 | 3.81 | 1.574791319 | 1.572178478 | 2.475853018 |
| Elandsfontein | 21.223 | 12.84 | 6.69 | 1.65288162 | 1.919282511 | 3.172346786 |
| Elandsfontein | 20.896 | 10.74 | 5.76 | 1.945623836 | 1.864583333 | 3.627777778 |
| Elandsfontein | 16.93 | 9.58 | 6.88 | 1.767223382 | 1.39244186 | 2.460755814 |
| Elandsfontein | 13.863 | 7.67 | 5.02 | 1.807431551 | 1.527888446 | 2.761553785 |
| Elandsfontein | 13.801 | 7.59 | 4.9 | 1.81831357 | 1.548979592 | 2.816530612 |
| Elandsfontein | 11.54 | 7.27 | 5.37 | 1.587345254 | 1.353817505 | 2.148975791 |
| Elandsfontein | 11.532 | 8.29 | 5.02 | 1.391073583 | 1.651394422 | 2.297211155 |
| Elandsfontein | 12.193 | 8.91 | 5.25 | 1.368462402 | 1.697142857 | 2.32247619 |
| Elandsfontein | 7.715 | 5 | 2.74 | 1.543 | 1.824817518 | 2.815693431 |
| Elandsfontein | 8.055 | 5.52 | 3.32 | 1.45923913 | 1.662650602 | 2.426204819 |
| Elandsfontein | 6.907 | 5.16 | 3.09 | 1.338565891 | 1.669902913 | 2.235275081 |
| Elandsfontein | 17.592 | 10.1 | 4.59 | 1.741782178 | 2.20043573 | 3.832679739 |
| Elandsfontein | 13.056 | 9.58 | 4.84 | 1.362839248 | 1.979338843 | 2.697520661 |
| Elandsfontein | 8.169 | 5.22 | 2.85 | 1.564942529 | 1.831578947 | 2.866315789 |
| Elandsfontein | 9.195 | 5.4 | 2.97 | 1.702777778 | 1.818181818 | 3.095959596 |
| Elandsfontein | 9.389 | 5.9 | 2.79 | 1.591355932 | 2.114695341 | 3.365232975 |
| Elandsfontein | 8.296 | 5.51 | 3.18 | 1.505626134 | 1.732704403 | 2.608805031 |
| Elandsfontein | 8.819 | 5.35 | 2.17 | 1.648411215 | 2.465437788 | 4.0640553 |
| Elandsfontein | 8.625 | 4.67 | 2.28 | 1.846895075 | 2.048245614 | 3.782894737 |
| Elandsfontein | 9.904 | 6.38 | 2.86 | 1.552351097 | 2.230769231 | 3.462937063 |
| Elandsfontein | 9.405 | 5.25 | 3.44 | 1.791428571 | 1.526162791 | 2.734011628 |
| Elandsfontein | 7.599 | 5.9 | 2.87 | 1.287966102 | 2.055749129 | 2.647735192 |
| Elandsfontein | 8.79 | 5.95 | 3.63 | 1.477310924 | 1.639118457 | 2.421487603 |
| Elandsfontein | 7.967 | 5.8 | 2.64 | 1.37362069 | 2.196969697 | 3.01780303 |
| Elandsfontein | 7.969 | 5.57 | 2.64 | 1.43070018 | 2.109848485 | 3.018560606 |
| Elandsfontein | 7.157 | 5.05 | 2.44 | 1.417227723 | 2.069672131 | 2.933196721 |
| Elandsfontein | 7.838 | 4.85 | 2.98 | 1.616082474 | 1.627516779 | 2.630201342 |
| Elandsfontein | 18.665 | 11.02 | 5.35 | 1.693738657 | 2.059813084 | 3.488785047 |
| Elandsfontein | 17.201 | 9.88 | 4.12 | 1.740991903 | 2.398058252 | 4.175 |
| Elandsfontein | 13.165 | 6.86 | 3.42 | 1.91909621 | 2.005847953 | 3.849415205 |
| Elandsfontein | 8.461 | 5.66 | 2.3 | 1.494876325 | 2.460869565 | 3.678695652 |
| Elandsfontein | 7.992 | 5.21 | 2.87 | 1.533973129 | 1.81533101 | 2.78466899 |
| Elandsfontein | 15.119 | 10.63 | 5.54 | 1.42229539 | 1.918772563 | 2.729061372 |
| Elandsfontein | 8.361 | 6.34 | 3.29 | 1.318769716 | 1.927051672 | 2.541337386 |
| Elandsfontein | 8.303 | 5.5 | 2.94 | 1.509636364 | 1.870748299 | 2.82414966 |
| Elandsfontein | 19.873 | 12.07 | 5.48 | 1.646478873 | 2.202554745 | 3.626459854 |
| Elandsfontein | 13.183 | 7.46 | 5.12 | 1.767158177 | 1.45703125 | 2.574804688 |
| Elandsfontein | 10.625 | 6.48 | 3.09 | 1.639660494 | 2.097087379 | 3.438511327 |
| Elandsfontein | 7.916 | 5.82 | 3.1 | 1.360137457 | 1.877419355 | 2.553548387 |
| Elandsfontein | 10.519 | 6.88 | 3.11 | 1.528924419 | 2.21221865 | 3.382315113 |
| Elandsfontein | 18.124 | 11.63 | 8.88 | 1.558383491 | 1.309684685 | 2.040990991 |
| Elandsfontein | 11.786 | 8.15 | 3.69 | 1.446134969 | 2.208672087 | 3.19403794 |
| Elandsfontein | 11.128 | 7.63 | 3.72 | 1.458453473 | 2.051075269 | 2.991397849 |
| Elandsfontein | 20.015 | 8.08 | 7.7 | 2.47710396 | 1.049350649 | 2.599350649 |
| Elandsfontein | 14.942 | 8.91 | 5.06 | 1.676992144 | 1.760869565 | 2.952964427 |
| Elandsfontein | 10.964 | 7.13 | 4.31 | 1.53772791 | 1.654292343 | 2.543851508 |
| Elandsfontein | 14.606 | 8.43 | 6.14 | 1.73262159 | 1.372964169 | 2.378827362 |
| Elandsfontein | 13.953 | 9.34 | 5.58 | 1.493897216 | 1.673835125 | 2.500537634 |
| Elandsfontein | 10.94 | 8.18 | 4.83 | 1.337408313 | 1.693581781 | 2.265010352 |
| Elandsfontein | 14.65 | 8.23 | 5.33 | 1.780072904 | 1.544090056 | 2.748592871 |
| Elandsfontein | 17.399 | 9.46 | 4.05 | 1.839217759 | 2.335802469 | 4.296049383 |
| Elandsfontein | 18.938 | 10.13 | 4.52 | 1.869496545 | 2.241150442 | 4.189823009 |
| Elandsfontein | 16.307 | 9.03 | 3.37 | 1.805869324 | 2.679525223 | 4.838872404 |
| Elandsfontein | 16.903 | 9.78 | 3.82 | 1.728323108 | 2.560209424 | 4.42486911 |
| Elandsfontein | 13.601 | 8.71 | 3.96 | 1.561538462 | 2.199494949 | 3.43459596 |
| Elandsfontein | 14.902 | 8.25 | 5.15 | 1.80630303 | 1.601941748 | 2.893592233 |
| Elandsfontein | 13.368 | 9.14 | 3.41 | 1.462582057 | 2.680351906 | 3.920234604 |
| Elandsfontein | 15.176 | 9.2 | 4.44 | 1.649565217 | 2.072072072 | 3.418018018 |
| Elandsfontein | 14.213 | 9.82 | 6.02 | 1.447352342 | 1.631229236 | 2.360963455 |
| Elandsfontein | 16.168 | 9.32 | 5.91 | 1.734763948 | 1.576988156 | 2.7357022 |
| Elandsfontein | 18.411 | 12.41 | 7.51 | 1.483561644 | 1.652463382 | 2.451531292 |
| Elandsfontein | 10.685 | 7.22 | 4.46 | 1.479916898 | 1.618834081 | 2.39573991 |
| Elandsfontein | 12.288 | 8.23 | 4.05 | 1.493074119 | 2.032098765 | 3.034074074 |
| Elandsfontein | 14.955 | 8.81 | 5.33 | 1.697502838 | 1.652908068 | 2.805816135 |
| Elandsfontein | 18.733 | 9.24 | 6.15 | 2.027380952 | 1.502439024 | 3.04601626 |
| Elandsfontein | 14.018 | 8.78 | 4.44 | 1.596583144 | 1.977477477 | 3.157207207 |
| Elandsfontein | 13.408 | 10.02 | 3.93 | 1.338123752 | 2.549618321 | 3.411704835 |
| Elandsfontein | 12.295 | 7.78 | 4.39 | 1.58033419 | 1.772209567 | 2.800683371 |
| Elandsfontein | 11.944 | 7.51 | 3.8 | 1.590412783 | 1.976315789 | 3.143157895 |
| Elandsfontein | 13.134 | 8.48 | 4.55 | 1.548820755 | 1.863736264 | 2.886593407 |
| Elandsfontein | 12.966 | 8.06 | 3.51 | 1.608684864 | 2.296296296 | 3.694017094 |
| Elandsfontein | 13.656 | 7.9 | 3.6 | 1.728607595 | 2.194444444 | 3.793333333 |
| Elandsfontein | 9.081 | 5.14 | 3.88 | 1.766731518 | 1.324742268 | 2.340463918 |
| Elandsfontein | 9.879 | 6.33 | 2.61 | 1.560663507 | 2.425287356 | 3.785057471 |
| Elandsfontein | 7.954 | 5.05 | 3.21 | 1.575049505 | 1.573208723 | 2.47788162 |
| Elandsfontein | 10.049 | 6.29 | 2.58 | 1.597615262 | 2.437984496 | 3.89496124 |
| Elandsfontein | 9.303 | 5.68 | 2.99 | 1.637852113 | 1.899665552 | 3.111371237 |
| Elandsfontein | 9.288 | 6.39 | 2.73 | 1.453521127 | 2.340659341 | 3.402197802 |
| Elandsfontein | 9.277 | 5.05 | 3.39 | 1.837029703 | 1.489675516 | 2.736578171 |
| Elandsfontein | 9.208 | 5.69 | 3.39 | 1.61827768 | 1.678466077 | 2.716224189 |
| Elandsfontein | 9.844 | 6.89 | 3.57 | 1.4287373 | 1.929971989 | 2.757422969 |
| Elandsfontein | 9.76 | 7.2 | 4.13 | 1.355555556 | 1.743341404 | 2.363196126 |
| Elandsfontein | 8.266 | 6.06 | 3.51 | 1.364026403 | 1.726495726 | 2.354985755 |
| Elandsfontein | 9.979 | 6.39 | 3.23 | 1.561658842 | 1.978328173 | 3.089473684 |
| Elandsfontein | 9.486 | 5.97 | 3.11 | 1.588944724 | 1.919614148 | 3.050160772 |
| Elandsfontein | 12.37 | 7.34 | 4.9 | 1.685286104 | 1.497959184 | 2.524489796 |
| Elandsfontein | 10.03 | 8.39 | 4.9 | 1.195470799 | 1.712244898 | 2.046938776 |
| Elandsfontein | 10.366 | 6.91 | 3.05 | 1.500144718 | 2.26557377 | 3.398688525 |
| Elandsfontein | 9.951 | 5.91 | 2.58 | 1.683756345 | 2.290697674 | 3.856976744 |
| Elandsfontein | 10.182 | 8.07 | 3.19 | 1.261710037 | 2.529780564 | 3.19184953 |
| Elandsfontein | 8.61 | 5.47 | 2.4 | 1.574040219 | 2.279166667 | 3.5875 |
| Elandsfontein | 9.145 | 5.99 | 3.41 | 1.526711185 | 1.75659824 | 2.681818182 |
| Elandsfontein | 9.307 | 5.51 | 3.49 | 1.689110708 | 1.578796562 | 2.666762178 |
| Elandsfontein | 7.013 | 4.7 | 2.54 | 1.49212766 | 1.850393701 | 2.761023622 |
| Elandsfontein | 8.017 | 5.28 | 3.73 | 1.518371212 | 1.415549598 | 2.149329759 |
| Elandsfontein | 14.095 | 8.77 | 8.32 | 1.60718358 | 1.054086538 | 1.694110577 |
| Elandsfontein | 15.134 | 7.83 | 6.76 | 1.932822478 | 1.158284024 | 2.238757396 |
| Elandsfontein | 11.48 | 7.75 | 4.17 | 1.481290323 | 1.858513189 | 2.752997602 |
| Elandsfontein | 20.949 | 9.49 | 6.81 | 2.20748156 | 1.393538913 | 3.076211454 |
| Elandsfontein | 15.018 | 10.9 | 4.3 | 1.377798165 | 2.534883721 | 3.49255814 |
| Elandsfontein | 15.671 | 7.34 | 4.42 | 2.135013624 | 1.660633484 | 3.545475113 |
| Elandsfontein | 11.648 | 6.37 | 2.92 | 1.828571429 | 2.181506849 | 3.989041096 |
| Elandsfontein | 13.934 | 8.76 | 4.07 | 1.590639269 | 2.152334152 | 3.423587224 |
| Elandsfontein | 11.938 | 6.29 | 3.61 | 1.897933227 | 1.742382271 | 3.306925208 |
| Elandsfontein | 14.565 | 8.86 | 5.6 | 1.643905192 | 1.582142857 | 2.600892857 |
| Elandsfontein | 12.098 | 7.5 | 4.35 | 1.613066667 | 1.724137931 | 2.781149425 |
| Elandsfontein | 13.84 | 7.1 | 4.34 | 1.949295775 | 1.6359447 | 3.188940092 |
| Elandsfontein | 10.553 | 6.44 | 3.52 | 1.638664596 | 1.829545455 | 2.998011364 |
| Elandsfontein | 15.91 | 9.86 | 4.79 | 1.613590264 | 2.058455115 | 3.321503132 |
| Elandsfontein | 16.961 | 10.15 | 4.14 | 1.671034483 | 2.451690821 | 4.096859903 |
| Elandsfontein | 16.704 | 8.31 | 4.45 | 2.010108303 | 1.86741573 | 3.753707865 |
| Elandsfontein | 14.102 | 8.04 | 3.96 | 1.7539801 | 2.03030303 | 3.561111111 |
| Elandsfontein | 17.06 | 8.17 | 5.96 | 2.088127295 | 1.370805369 | 2.862416107 |
| Elandsfontein | 13.512 | 8.54 | 5.81 | 1.582201405 | 1.469879518 | 2.325645439 |
| Elandsfontein | 15.807 | 10.27 | 4.07 | 1.539143135 | 2.523341523 | 3.883783784 |
| Elandsfontein | 14.292 | 10.08 | 4.32 | 1.417857143 | 2.333333333 | 3.308333333 |
| Elandsfontein | 13.387 | 8.4 | 4.51 | 1.593690476 | 1.862527716 | 2.968292683 |
| Elandsfontein | 13.303 | 9.22 | 4.32 | 1.442841649 | 2.134259259 | 3.079398148 |
| Elandsfontein | 11.316 | 7.12 | 2.88 | 1.589325843 | 2.472222222 | 3.929166667 |
| Elandsfontein | 11.345 | 7.67 | 3.39 | 1.479139505 | 2.262536873 | 3.34660767 |
| Elandsfontein | 12.171 | 7.72 | 4.45 | 1.576554404 | 1.734831461 | 2.73505618 |
| Elandsfontein | 15.828 | 8.54 | 3.85 | 1.853395785 | 2.218181818 | 4.111168831 |
| Elandsfontein | 11.183 | 6.9 | 3.48 | 1.620724638 | 1.982758621 | 3.213505747 |
| Elandsfontein | 10.556 | 5.84 | 3.31 | 1.807534247 | 1.764350453 | 3.189123867 |
| Elandsfontein | 9.44 | 6.68 | 3.26 | 1.413173653 | 2.049079755 | 2.895705521 |
| Elandsfontein | 10.603 | 6.61 | 3.1 | 1.60408472 | 2.132258065 | 3.420322581 |
| Elandsfontein | 10.29 | 6.36 | 3.52 | 1.617924528 | 1.806818182 | 2.923295455 |
| Elandsfontein | 11.884 | 6.61 | 4.68 | 1.797881997 | 1.412393162 | 2.539316239 |
| Elandsfontein | 18.144 | 9.43 | 3.79 | 1.92407211 | 2.488126649 | 4.787335092 |
| Elandsfontein | 15.093 | 10.35 | 3.98 | 1.45826087 | 2.600502513 | 3.792211055 |
| Elandsfontein | 17.276 | 10.27 | 5.82 | 1.68218111 | 1.764604811 | 2.96838488 |
| Elandsfontein | 13.787 | 9.96 | 4.43 | 1.384236948 | 2.248306998 | 3.112189616 |
| Elandsfontein | 16.07 | 8.77 | 4.69 | 1.832383124 | 1.869936034 | 3.426439232 |
| Elandsfontein | 15.576 | 8.17 | 4.09 | 1.906487148 | 1.997555012 | 3.808312958 |
| Elandsfontein | 12.601 | 8.89 | 4.46 | 1.417435321 | 1.993273543 | 2.825336323 |
| Elandsfontein | 10.586 | 5.53 | 3.37 | 1.914285714 | 1.640949555 | 3.141246291 |
| Elandsfontein | 10.076 | 6.79 | 3.72 | 1.483946981 | 1.825268817 | 2.708602151 |
| Elandsfontein | 18.324 | 9.94 | 5.68 | 1.843460765 | 1.75 | 3.226056338 |
| Elandsfontein | 17.569 | 11.18 | 6.95 | 1.571466905 | 1.608633094 | 2.527913669 |
| Elandsfontein | 13.331 | 7.9 | 3.58 | 1.687468354 | 2.206703911 | 3.723743017 |
| Elandsfontein | 12.524 | 8.59 | 4.59 | 1.457974389 | 1.871459695 | 2.728540305 |
| Elandsfontein | 9.76 | 8.07 | 3.23 | 1.209417596 | 2.498452012 | 3.021671827 |
| Elandsfontein | 10.013 | 7.16 | 3.31 | 1.398463687 | 2.163141994 | 3.025075529 |
| Dungo IV | 5.6 | 4.4 | 4.3 | 1.272727273 | 1.023255814 | 1.302325581 |
| Dungo IV | 7.22 | 5.2 | 4.4 | 1.388461538 | 1.181818182 | 1.640909091 |
| Dungo IV | 6.9 | 4.7 | 4.5 | 1.468085106 | 1.044444444 | 1.533333333 |
| Dungo IV | 11.3 | 6.8 | 6.5 | 1.661764706 | 1.046153846 | 1.738461538 |
| Dungo IV | 14 | 6.9 | 6.6 | 2.028985507 | 1.045454545 | 2.121212121 |
| Dungo IV | 16.9 | 8.9 | 8.8 | 1.898876404 | 1.011363636 | 1.920454545 |
| Dungo IV | 10.1 | 7 | 6 | 1.442857143 | 1.166666667 | 1.683333333 |
| Dungo IV | 10.6 | 8.2 | 6.6 | 1.292682927 | 1.242424242 | 1.606060606 |
| Dungo IV | 9.9 | 5.3 | 4.9 | 1.867924528 | 1.081632653 | 2.020408163 |
| Dungo IV | 9.2 | 8.2 | 7.8 | 1.12195122 | 1.051282051 | 1.179487179 |
| Dungo IV | 18.1 | 8.3 | 8.3 | 2.180722892 | 1 | 2.180722892 |
| Dungo IV | 9.7 | 7.9 | 6.6 | 1.227848101 | 1.196969697 | 1.46969697 |
| Dungo IV | 9.1 | 6 | 4.9 | 1.516666667 | 1.224489796 | 1.857142857 |
| Dungo IV | 8.9 | 6.4 | 6.4 | 1.390625 | 1 | 1.390625 |
| Dungo IV | 7.1 | 8.3 | 8.2 | 0.855421687 | 1.012195122 | 0.865853659 |
| Dungo IV | 13 | 7.9 | 7.9 | 1.64556962 | 1 | 1.64556962 |
| Dungo IV | 7.4 | 4.9 | 4.9 | 1.510204082 | 1 | 1.510204082 |
| Dungo IV | 9.7 | 8.6 | 3.2 | 1.127906977 | 2.6875 | 3.03125 |
| Dungo IV | 6.4 | 4.7 | 4.7 | 1.361702128 | 1 | 1.361702128 |
| Dungo IV | 10.2 | 8 | 8 | 1.275 | 1 | 1.275 |
| Dungo IV | 11.4 | 5.4 | 4.6 | 2.111111111 | 1.173913043 | 2.47826087 |
| Dungo IV | 16 | 8 | 8 | 2 | 1 | 2 |
| Dungo IV | 7.2 | 5.5 | 5.5 | 1.309090909 | 1 | 1.309090909 |
| Dungo IV | 8 | 5.8 | 5.8 | 1.379310345 | 1 | 1.379310345 |
