# Supplementary material for: Evidence for Earlier Stone Age ‘coastal use’: The site of Dungo IV, Benguela Province, Angola
Source: PLoS One. 2023 Feb 24;18(2):e0278775. doi: 10.1371/journal.pone.0278775 (PMC9955982; doi:10.1371/journal.pone.0278775)
Supplement: S3 Table — Results are presented for each qualitative variable and for each site. (DOCX) [file pone.0278775.s003.docx]

**Supplementary Information – File 3**

The following Table presents the results of the Tukey HSD Test for each quantitative variable test during the inter-site comparison.

| FINENESS 1 | DIFF | LWR | UPR | P ADJ |
| --- | --- | --- | --- | --- |
| Dungo IV-Cape Hangklip | -1.50691381 | -1.8766585 | -1.137169092 | 0.0000000 |
| Elandsfontein-Cape Hangklip | -0.08262188 | -0.2301545 | 0.064910734 | 0.5987198 |
| Gemsbok-Cape Hangklip | -0.20394323 | -0.3998161 | -0.008070363 | 0.0356893 |
| Namib IV-Cape Hangklip | -0.33749745 | -0.9255753 | 0.250580371 | 0.5723263 |
| Penhill Farm-Cape Hangklip | -0.17585908 | -0.4638457 | 0.112127581 | 0.5024948 |
| Elandsfontein-Dungo IV | 1.42429193 | 1.0555048 | 1.793079073 | 0.0000000 |
| Gemsbok-Dungo IV | 1.30297057 | 0.9123246 | 1.693616536 | 0.0000000 |
| Namib IV-Dungo IV | 1.16941636 | 0.4911291 | 1.847703619 | 0.0000153 |
| Penhill Farm-Dungo IV | 1.33105473 | 0.8870115 | 1.775097972 | 0.0000000 |
| Gemsbok-Elandsfontein | -0.12132135 | -0.3153806 | 0.072737881 | 0.4752547 |
| Namib IV-Elandsfontein | -0.25487557 | -0.8423518 | 0.332600666 | 0.8172060 |
| Penhill Farm-Elandsfontein | -0.09323720 | -0.3799934 | 0.193519006 | 0.9389980 |
| Namib IV-Gemsbok | -0.13355422 | -0.7349930 | 0.467884589 | 0.9884092 |
| Penhill Farm-Gemsbok | 0.02808415 | -0.2862873 | 0.342455638 | 0.9998529 |
| Penhill Farm-Namib IV | 0.16163837 | -0.4757773 | 0.799054066 | 0.9789312 |
|  |  |  |  |  |
| FINENESS 2 | **DIFF** | **LWR** | **UPR** | **P ADJ** |
| Dungo IV-Cape Hangklip | -1.50691381 | -1.8766585 | -1.137169092 | 0.0000000 |
| Elandsfontein-Cape Hangklip | -0.08262188 | -0.2301545 | 0.064910734 | 0.5987198 |
| Gemsbok-Cape Hangklip | -0.20394323 | -0.3998161 | -0.008070363 | 0.0356893 |
| Namib IV-Cape Hangklip | -0.33749745 | -0.9255753 | 0.250580371 | 0.5723263 |
| Penhill Farm-Cape Hangklip | -0.17585908 | -0.4638457 | 0.112127581 | 0.5024948 |
| Elandsfontein-Dungo IV | 1.42429193 | 1.0555048 | 1.793079073 | 0.0000000 |
| Gemsbok-Dungo IV | 1.30297057 | 0.9123246 | 1.693616536 | 0.0000000 |
| Namib IV-Dungo IV | 1.16941636 | 0.4911291 | 1.847703619 | 0.0000153 |
| Penhill Farm-Dungo IV | 1.33105473 | 0.8870115 | 1.775097972 | 0.0000000 |
| Gemsbok-Elandsfontein | -0.12132135 | -0.3153806 | 0.072737881 | 0.4752547 |
| Namib IV-Elandsfontein | -0.25487557 | -0.8423518 | 0.332600666 | 0.8172060 |
| Penhill Farm-Elandsfontein | -0.09323720 | -0.3799934 | 0.193519006 | 0.9389980 |
| Namib IV-Gemsbok | -0.13355422 | -0.7349930 | 0.467884589 | 0.9884092 |
| Penhill Farm-Gemsbok | 0.02808415 | -0.2862873 | 0.342455638 | 0.9998529 |
| Penhill Farm-Namib IV | 0.16163837 | -0.4757773 | 0.799054066 | 0.9789312 |
|  |  |  |  |  |
| LENGTH | **DIFF** | **LWR** | **UPR** | **P ADJ** |
| Dungo IV-Cape Hangklip | -5.30340174 | -7.448200 | -3.158603001 | 0.0000000 |
| Elandsfontein-Cape Hangklip | -2.59373158 | -3.449532 | -1.737930939 | 0.0000000 |
| Gemsbok-Cape Hangklip | -0.99105940 | -2.127270 | 0.145151253 | 0.1276568 |
| Namib IV-Cape Hangklip | -4.48895730 | -7.900253 | -1.077661566 | 0.0025201 |
| Penhill Farm-Cape Hangklip | -5.21673507 | -6.887275 | -3.546194830 | 0.0000000 |
| Elandsfontein-Dungo IV | 2.70967016 | 0.570426 | 4.848914286 | 0.0042585 |
| Gemsbok-Dungo IV | 4.31234234 | 2.046301 | 6.578384118 | 0.0000011 |
| Namib IV-Dungo IV | 0.81444444 | -3.120134 | 4.749023106 | 0.9916040 |
| Penhill Farm-Dungo IV | 0.08666667 | -2.489120 | 2.662453030 | 0.9999989 |
| Gemsbok-Elandsfontein | 1.60267218 | 0.476982 | 2.728362380 | 0.0007446 |
| Namib IV-Elandsfontein | -1.89522572 | -5.303032 | 1.512580371 | 0.6060806 |
| Penhill Farm-Elandsfontein | -2.62300350 | -4.286406 | -0.959600812 | 0.0001121 |
| Namib IV-Gemsbok | -3.49789790 | -6.986697 | -0.009098354 | 0.0489683 |
| Penhill Farm-Gemsbok | -4.22567568 | -6.049268 | -2.402083506 | 0.0000000 |
| Penhill Farm-Namib IV | -0.72777778 | -4.425270 | 2.969714929 | 0.9933525 |
|  |  |  |  |  |
| WIDTH | **DIFF** | **LWR** | **UPR** | **P ADJ** |
| Dungo IV-Cape Hangklip | -2.5862562 | -3.766387088 | -1.4061253 | 0.0000000 |
| Elandsfontein-Cape Hangklip | -1.4035231 | -1.874409610 | -0.9326366 | 0.0000000 |
| Gemsbok-Cape Hangklip | -0.4377202 | -1.062896447 | 0.1874561 | 0.3429270 |
| Namib IV-Cape Hangklip | -2.9070896 | -4.784084032 | -1.0300951 | 0.0001609 |
| Penhill Farm-Cape Hangklip | -2.7023276 | -3.621507650 | -1.7831476 | 0.0000000 |
| Elandsfontein-Dungo IV | 1.1827331 | 0.005658544 | 2.3598077 | 0.0481123 |
| Gemsbok-Dungo IV | 2.1485360 | 0.901693710 | 3.3953784 | 0.0000155 |
| Namib IV-Dungo IV | -0.3208333 | -2.485753356 | 1.8440867 | 0.9982659 |
| Penhill Farm-Dungo IV | -0.1160714 | -1.533344268 | 1.3012014 | 0.9999042 |
| Gemsbok-Elandsfontein | 0.9658029 | 0.346415331 | 1.5851905 | 0.0001407 |
| Namib IV-Elandsfontein | -1.5035664 | -3.378640811 | 0.3715079 | 0.1989025 |
| Penhill Farm-Elandsfontein | -1.2988045 | -2.214057237 | -0.3835518 | 0.0007864 |
| Namib IV-Gemsbok | -2.4693694 | -4.389008710 | -0.5497300 | 0.0034604 |
| Penhill Farm-Gemsbok | -2.2646075 | -3.268001104 | -1.2612138 | 0.0000000 |
| Penhill Farm-Namib IV | 0.2047619 | -1.829706505 | 2.2392303 | 0.9997357 |
|  |  |  |  |  |
| THICKNESS | **DIFF** | **LWR** | **UPR** | **P ADJ** |
| Dungo IV-Cape Hangklip | 1.18088308 | 0.3961817 | 1.96558446 | 0.0002794 |
| Elandsfontein-Cape Hangklip | -0.72760176 | -1.0407071 | -0.41449642 | 0.0000000 |
| Gemsbok-Cape Hangklip | 0.02840561 | -0.3872912 | 0.44410245 | 0.9999609 |
| Namib IV-Cape Hangklip | -1.02745025 | -2.2755153 | 0.22061485 | 0.1748349 |
| Penhill Farm-Cape Hangklip | -1.42745025 | -2.0386382 | -0.81626226 | 0.0000000 |
| Elandsfontein-Dungo IV | -1.90848485 | -2.6911540 | -1.12581570 | 0.0000000 |
| Gemsbok-Dungo IV | -1.15247748 | -1.9815371 | -0.32341783 | 0.0011003 |
| Namib IV-Dungo IV | -2.20833333 | -3.6478480 | -0.76881865 | 0.0001939 |
| Penhill Farm-Dungo IV | -2.60833333 | -3.5507169 | -1.66594976 | 0.0000000 |
| Gemsbok-Elandsfontein | 0.75600737 | 0.3441596 | 1.16785517 | 0.0000030 |
| Namib IV-Elandsfontein | -0.29984848 | -1.5466369 | 0.94693988 | 0.9833680 |
| Penhill Farm-Elandsfontein | -0.69984848 | -1.3084251 | -0.09127186 | 0.0135577 |
| Namib IV-Gemsbok | -1.05585586 | -2.3322767 | 0.22056498 | 0.1706057 |
| Penhill Farm-Gemsbok | -1.45585586 | -2.1230398 | -0.78867191 | 0.0000000 |
| Penhill Farm-Namib IV | -0.40000000 | -1.7527738 | 0.95277383 | 0.9589450 |
